# Retrospective analysis of reference intervals for dried blood spot based ms/ms newborn screening programs in Chinese preterm neonates: a nationwide study

**DOI:** 10.1186/s12887-024-04865-1

**Published:** 2024-07-02

**Authors:** Falin He, Tiancheng Xie, Xinwen Huang, Jinming Zhang, Tian Tang

**Affiliations:** 1 National Center for Clinical Laboratories, Institute of Geriatric Medicine, Chinese Academy of Medical Sciences, Beijing Hospital, National Center of Gerontology, Beijing, P. R. China; 2 Department of Laboratory Sciences, West China School of Public Health and West China No.4 Hospital, Sichuan University, Chengdu, Sichuan P. R. China; 3 Department of Genetics and Metabolism, Children’s Hospital of Zhejiang University School of Medicine, National Clinical Research Center for Child Health, Hangzhou, P. R. China

**Keywords:** Preterm infants, Newborn screening, Inborn errors of metabolism, Mass spectrometry, Reference intervals

## Abstract

**Objectives:**

Although recent discoveries regarding the biomarkers of newborn screening (NBS) programs by tandem mass spectrometry (MS/MS) highlight the critical need to establish reference intervals (RIs) specifically for preterm infants, no such RIs has been formally published yet. This study addressed the gap by offering a comprehensive set of reference intervals (RIs) for preterm neonates, and illustrating the dynamic changes of each biomarker with age.

**Design and methods:**

The NBS data of 199,693 preterm newborns (< 37 weeks of gestation) who met the inclusion and exclusion criteria from the NNSCP database were included in study analysis. The birth weight stratified dynamic trend of each biomarker were captured by their concentrations over age. Reference partitions were determined by the method of Harris and Boyd. RIs, corresponding to the 2.5th and 97.5th percentiles, as well as the 0.5th, 25th, 50th, 75th and 99.5th percentiles were calculated using a non-parametric rank approach.

**Results:**

Increasing birth weight is associated with an elevation in the levels of arginine, citrulline, glycine, leucine and isobarics, methionine, ornithine, phenylalanine, and valine, whereas the levels of alanine, proline and tyrosine decrease. Additionally, two short-chain acylcarnitines (butyrylcarnitine + isobutyrylcarnitine and isovalerylcarnitine + methylbutyrylcarnitine) and a median-chain acylcarnitine (octenoylcarnitine) decrease, while four long-chain acylcarnitines (tetradecanoylcarnitine, palmitoylcarnitine, palmitoleylcarnitine and oleoylcarnitine) increase with increasing birth weight. Age impacts the levels of all MS/MS NBS biomarkers, while sex only affects the level of malonylcarnitine + 3-hydroxybutyrylcarnitine (C3-DC + C4-OH) in very low birth weight preterm neonates.

**Conclusion:**

The current study developed reference intervals (RIs) specific to birth weight, age, and/or sex for 35 MS/MS biomarkers, which can help in the timely evaluation of the health and disease of preterm neonates.

**Supplementary Information:**

The online version contains supplementary material available at 10.1186/s12887-024-04865-1.

## Introduction

Congenital Inherited Metabolic Disorders (IMDs) refer to a group of genetic conditions that impair the body’s ability to break down specific nutrients or chemicals, leading to the accumulation of harmful substances in the bloodstream or tissues [1, 2]. Newborn screening (NBS) programs that utilize tandem mass spectrometry (MS/MS) are specifically designed to detect certain IMDs in infants immediately after birth [3, 4]. The timely identification of these disorders is crucial as many of them require prompt treatment to prevent severe health problems and complications.

For the MS/MS NBS programs, reference intervals (RIs) play a key role in helping healthcare providers interpret the results of screening tests and identify infants who may have an underlying medical condition that requires further evaluation and treatment [5, 6]. Given the importance of RIs in MS/MS NBS programs, the Chinese National Center for Clinical Laboratories (NCCL) launched a national newborn healthcare initiative in 2014, designated the Nationwide Newborn Screening Cooperative Program (NNSCP) [7]. In their pilot study, NNSCP published reference standards for 35 MS/MS NBS biomarkers [7]. However, these RIs only targeted full-term neonates.

Several studies have shown that preterm neonates may require distinct reference intervals (RIs) to precisely evaluate their health status, since the regular RIs, which are typically determined from full-term infants, often lead to a high rate of false-positive results in preterm infants [8, 9]. To address this issue, we analyzed data from the preterm newborns in the NNSCP database and developed age and/or sex-specific RIs for these infants. This will help healthcare providers more accurately identify the IMDs in preterm infants and ultimately improve their health outcomes.

## Methods

### Study population

Preterm neonates (< 37 weeks of gestation) from birth to 14 days of age from the NNSCP database with birth weight of ≤ 4.0 kg were selected as the study population [7]. Subjects were divided into 3 groups based on their birth weight: (1) very low birth weight group (VLBW) (1000 –1499 g); (2) low birth weight group (LBW) (1500 –2499 g); and (3) normal birth weight group (NBW) (2500–3999 g).

### Inclusion and exclusion criteria

Individuals were excluded in the case of acute/chronic illness, family history of hearing loss, use of medications or receiving full parenteral nutrition (FPN).

### Data source

Due to variations in instruments and test reagents, the data used for study analysis were exclusively sourced from NNSCP participating laboratories/centers, where dried blood samples were tested using the NeoBaseTM Non-derivatized MS/MS kit (PerkinElmer, MA, United States) on platforms including Waters Acquity TQD, Xevo TQD, or Quattro micro API-triple quadrupole mass spectrometry, as well as AB Sciex QTRAP 3200, 4,000, or 4500-hybrid triple quadrupole/linear ion trap mass spectrometry. The limits of detection (LoD), limits of quantification (LoQ), linear dynamic range and the total imprecision (TI) of analytes on platforms were described previously [7]. The allowable bias for each analyte obtained from analytical platforms was ≤ 10%, which is in accordance with the criteria of clinical laboratory standard institute (CLSI) [10].

### Statistical analysis

The data analysis was conducted in accordance with the CLSI EP28-A3c guidelines [11]. The statistical analysis was performed using Python and R programming languages. The data were visually inspected using boxplots, and any extreme outlying observations were identified by applying Box-Cox transformation in conjunction with Tukey’s fences (BCT). The Shapiro-Wilk test or the Kolmogorov-Smirnov test was utilized to assess the distribution of each analyte. The differences between any two birth weight groups of a given analyte were determined by the Kruskal-Wallis test. The resultant *p* value was adjusted to a *q* value using the false discovery rate (FDR) algorithm [12]. *q* < 0.05 indicated statistically significant. The reference intervals (RIs) of the analytes were determined using the method of Harris and Boyd [11]. The central 95% interval, which corresponds to the range between the 2.5th and 97.5th percentiles, was defined as the RI for each analyte. The 90% confidence intervals were calculated for the end points of each RI using the bootstrapping resampling method. The nonparametric rank method was employed to compute the 0.5st, 2.5th, 25th, 50th, 75th, 97.5th and 99.5th percentiles of each reference partition.

### Performance validation

The effectiveness of the RIs was validated using data from the NNSCP database. This involved comparing the levels of target biomarkers in true-positive, false-positive, and false-negative cases against the established RIs.

## Results

**Fig. 1:**
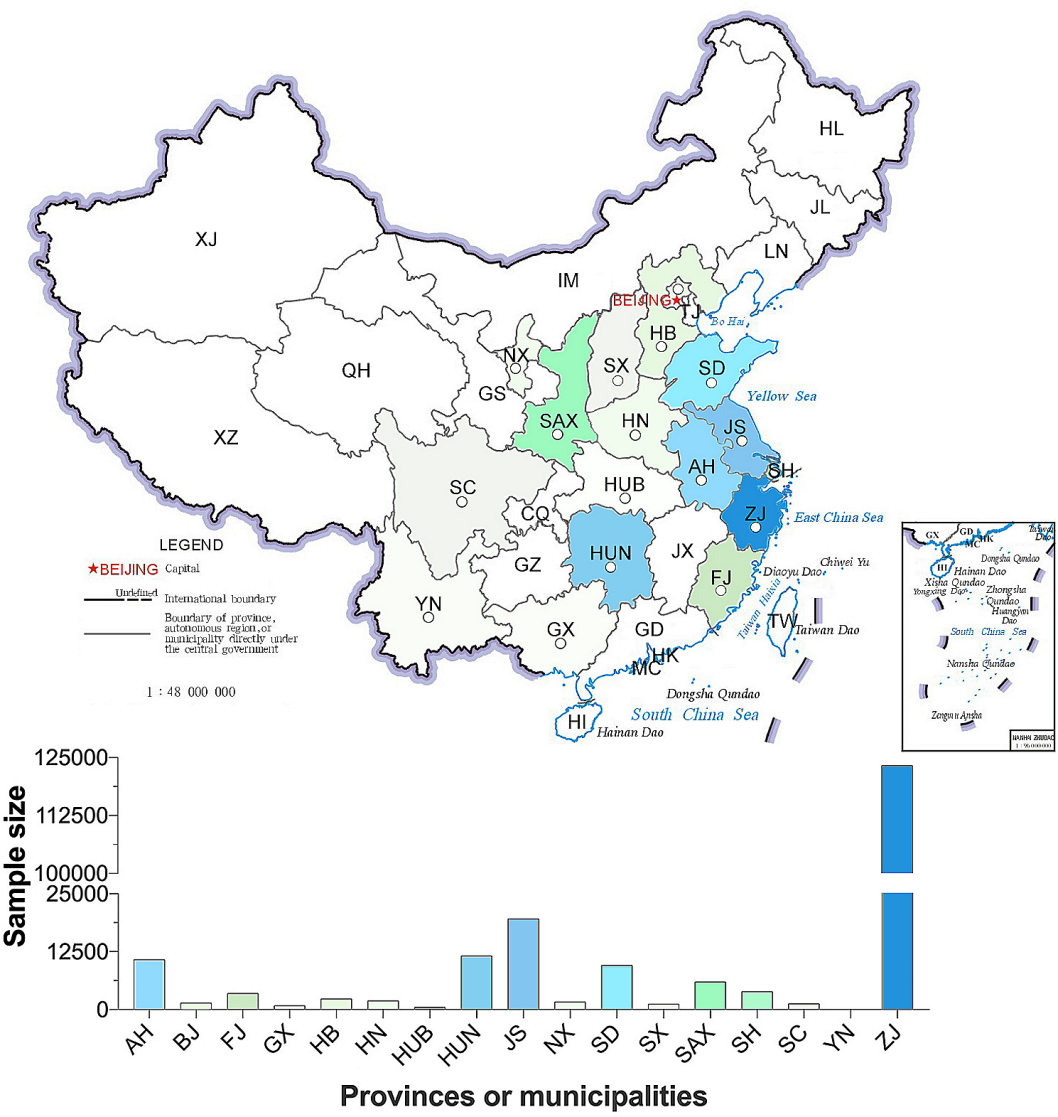
The distribution and sample size of study subjects at each province/municipality. HL, Heilongjiang; JL, Jilin; LN, Liaoning; XJ, Xinjiang; IM, Inner Mongolia; TJ, Tianjin, HE, Hebei; SX, Shanxi; SN, Shaanxi; NX, Ningxia; GS, Gansu; QH, Qinghai; SD, Shandong; JS, Jiangsu; AH, Anhui; HA, Henan; SH, Shanghai; HB, Hubei; CQ, Chongqing; SC, Sichuan; XZ, Tibet; ZJ, Zhejiang; JX, Jiangxi; HN, Hunan; GZ, Guizhou; YN, Yunnan; FJ, Fujian; TW, Taiwan; GD, Guangdong; GX, Guangxi; HK, Hongkong; MC, Macau. Areas with no data submitted show no color

This study analyzed data from 199,693 newborns who met the inclusion and exclusion criteria. The subjects were distributed across 17 provinces/municipalities in mainland China. However, data from Xinjiang, Qinghai, Tibet, Inner Mongolia, Heilongjiang, Jilin, Liaoning, Chongqing, Guizhou, Gansu, Guangdong, Hainan, Hong Kong, Macao, and Taiwan were not available (Fig. 1).

**Fig. 2:**
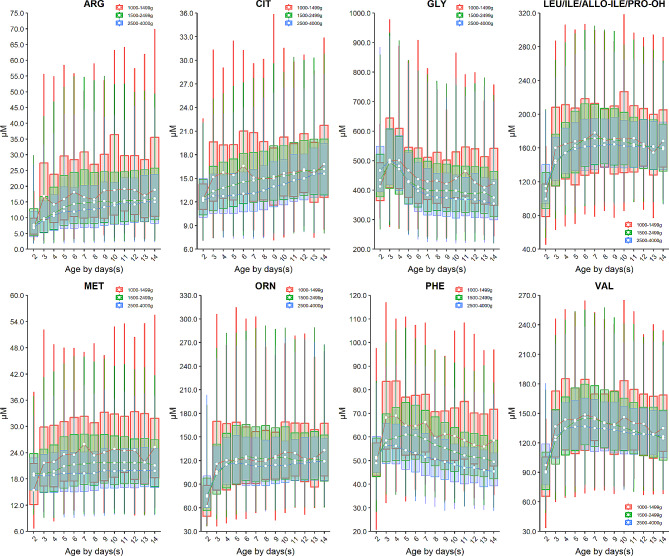
Dynamic change of amino acids whose level decreased with the decreasing birth weight. Data of VLBW, LBW and NBW are shown in red, green and blue boxes with whiskers, respectively. The boxes extend from the 25th to the 75th percentile, with whiskers extending to the 2.5th or 97.5th percentile. Medians are shown as white circles in the body of boxes, and are linked with red (VLBW), green (LBW) or blue (NBW) line to shown the dynamics. Abbreviations are listed in the legend of Table 1

**Fig. 3:**
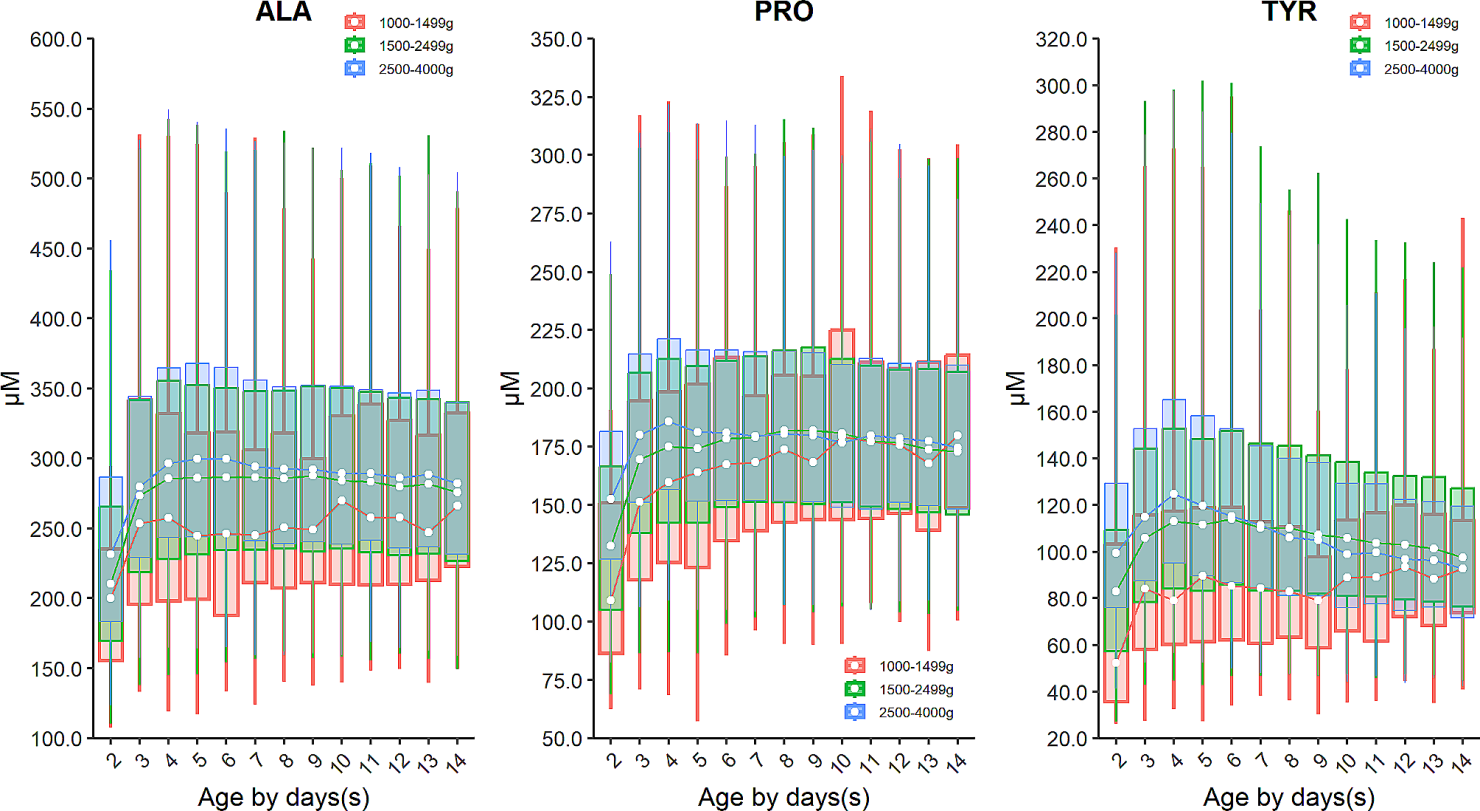
Dynamic change of amino acids whose level increased with the increasing birth weight. Data of VLBW, LBW and NBW are shown in red, green and blue boxes with whiskers, respectively. The boxes extend from the 25th to the 75th percentile, with whiskers extending to the 2.5th or 97.5th percentile. Medians are shown as white circles in the body of boxes, and are linked with red (VLBW), green (LBW) or blue (NBW) line to shown the dynamics. Abbreviations are listed in the legend of Table 1

Birth weight is an important factor affecting the level of amino acids. Specifically, the median of arginine (ARG), citrulline (CIT), glycine (GLY), leucine and isobarics (LEU/ILE/ALLO-ILE/PRO-OH), methionine (MET), ornithine (ORN), phenylalanine (PHE), and valine (VAL) increased with the increasing of birth weight (Fig. 2), while alanine (ALA), proline (PRO), and tyrosine (TYR) decreased over the birth weight (Fig. 3**)**. In addition to the birth weight, age also impacts the profiles of amino acids, as indicated by the levels of alanine (ALA), arginine (ARG), citrulline (CIT), leucine and isobarics (LEU/ILE/ALLO-ILE/PRO-OH), methionine (MET), ornithine (ORN), proline (PRO), and valine (VAL) elevated over age in the first 3 or 5 days after birth, followed by a steady value up to 14 days of age (Figs. 2 and 3). In contrast, the levels of glycine (GLY), phenylalanine (PHE), and tyrosine (TYR) enhanced rapidly in the first 3- or 5 days post-birth, and dropped thereafter (Figs. 2 and 3).

**Table 1:** RIs for 35 MS/MS NBS biomarkers in preterm neonates of 1000–1499 g (M)

**Analytes**	**Age**	**Lower limit (2.5th )** **and 90% CI**		**Upper limit (97.5th ) and 90% CI**		**No. of** **samples**
		Amino acids				
ALA	2 days to ≤ 14 days	135.5 (134.1-138.9)		497.0 (486.6-509.4)		3063
ARG	2 days to ≤ 14 days	3.2 (3.0-3.5)		58.4 (56.4–60.2)		3097
CIT	2 days to ≤ 14 days	8.2 (8.0-8.3)		31.0 (30.1–31.6)		3094
GLY	2 days to ≤ 14 days	262.4 (258.1-267.5)		834.4 (817.8-848.7)		3077
LEU/ILE/ ALLO-ILE/PRO-OH	2 days to ≤ 14 days	75.9 (72.7–78.6)		302.8 (296.7-306.9)		3100
MET	2 days to ≤ 14 days	10.4 (10.0-10.6)		51.1 (49.8–52.0)		3104
ORN	2 days to ≤ 14 days	50.8 (48.8–52.2)		272.5 (262.9-279.5)		3073
PHE	2 days to ≤ 14 days	36.6 (35.5–37.2)		109.2 (107.8-110.4)		3084
PRO	2 days to ≤ 14 days	85.9 (83.3–89.8)		310.2 (304.1-315.1)		3078
TYR	2 days to ≤ 14 days	36.8 (35.7–38.2)		242.5 (230.1-251.3)		3018
VAL	2 days to ≤ 14 days	67.3 (65.5–70.0)		248.7 (245.2-251.8)		3093
		Acylcarnitnes				
C0	2 days to ≤ 14 days	12.93 (12.73–13.20)		62.82 (60.63–66.39)		3084
C2	2 days to ≤ 4 days	9.75 (8.93-10.0)		54.04 (51.31–59.02)		978
	4 days to ≤ 6 days	7.06 (6.63–7.45)		44.03 (39.04–47.91)		511
	6 days to ≤ 14 days	5.18 (4.96–5.35)		27.67 (25.89–29.10)		1596
C3	2 days to ≤ 4 days	0.86 (0.82–0.90)		5.22 (4.98–5.67)		982
	4 days to ≤ 5 days	0.78 (0.71–0.86)		4.01 (3.73–5.36)		271
	5 days to ≤ 6 days	0.65 (0.57–0.68)		3.28 (2.89–3.63)		240
	6 days to ≤ 8 days	0.55 (0.53–0.58)		2.74 (2.43–3.06)		391
	8 days to ≤ 14 days	0.39 (0.37–0.42)		2.04 (1.93–2.14)		1197
C3-DC + C4-OH	2 days to ≤ 14 days	0.04 (0.04–0.05)		0.23 (0.22–0.25)		3046
C4	2 days to ≤ 12 days	0.14 (0.14–0.14)		0.56 (0.55–0.58)		2623
	12 days to ≤ 14 days	0.12 (0.12–0.13)		0.37 (0.34–0.40)		431
C4-DC + C5-OH(Male)	2 days to ≤ 14 days	0.11 (0.11–0.11)		0.37 (0.36–0.38)		1490
C4-DC + C5-OH(Female)	2 days to ≤ 14 days	0.10 (0.10–0.10)		0.32 (0.31–0.33)		1586
C5	2 days to ≤ 14 days	0.09 (0.09–0.10)		0.48 (0.47–0.49)		3048
C5-DC + C6-OH	2 days to ≤ 14 days	0.05 (0.05–0.05)		0.22 (0.22–0.23)		3096
C6	2 days to ≤ 14 days	0.02 (0.02–0.02)		0.12 (0.11–0.12)		3082
C6-DC	2 days to ≤ 14 days	0.03 (0.03–0.04)		0.21 (0.20–0.22)		3109
C8	2 days to ≤ 14 days	0.03 (0.03–0.03)		0.16 (0.15–0.17)		3058
C8:1	2 days to ≤ 14 days	0.05 (0.05–0.06)		0.43 (0.42–0.45)		3060
C10	2 days to ≤ 14 days	0.02 (0.02–0.02)		0.14 (0.14–0.15)		3079
C10:1	2 days to ≤ 14 days	0.03 (0.02–0.03)		0.14 (0.14–0.15)		3070
C12	2 days to ≤ 14 days	0.02 (0.02–0.02)		0.11 (0.10–0.11)		3076
C12:1	2 days to ≤ 14 days	0.01 (0.01–0.01)		0.11 (0.10–0.11)		3056
C14	2 days to ≤ 14 days	0.04 (0.04–0.05)		0.33 (0.31–0.34)		3090
C14:1	2 days to ≤ 5 days	0.03 (0.03–0.03)		0.15 (0.14–0.15)		1231
	5 days to ≤ 14 days	0.02 (0.02–0.02)		0.10 (0.09–0.10)		1811
C16	2 days to ≤ 8 days	0.66 (0.64–0.68)		4.38 (4.19–4.55)		1884
	8 days to ≤ 14 days	0.47 (0.46–0.49)		2.30 (2.22–2.51)		1199
C16:1	2 days to ≤ 5 days	0.04 (0.04–0.05)		0.40 (0.37–0.42)		1240
	5 days to ≤ 14 days	0.03 (0.03–0.03)		0.22 (0.19–0.23)		1807
C16:1-OH	2 days to ≤ 7 days	0.02 (0.02–0.02)		0.06 (0.06–0.06)		1566
	7 days to ≤ 10 days	0.01 (0.01–0.01)		0.05 (0.04–0.05)		541
	10 days to ≤ 12 days	0.02 (0.02–0.02)		0.04 (0.04–0.04)		322
	12 days to ≤ 14 days	0.01 (0.01–0.01)		0.04 (0.04–0.04)		406
C18	2 days to ≤ 11 days	0.34 (0.33–0.35)		1.43 (1.40–1.44)		2424
	11 days to ≤ 14 days	0.27 (0.26–0.28)		0.98 (0.94–1.02)		656
C18:1	2 days to ≤ 13 days	0.50 (0.49–0.52)		2.20 (2.14–2.27)		2835
	13 days to ≤ 14 days	0.43 (0.41–0.44)		1.61 (1.41–1.72)		222
C18:2	2 days to ≤ 14 days	0.15 (0.14–0.16)		1.11 (1.08–1.14)		3061

**Abbreviation**: ALA, alanine; ARG, arginine; CIT, citrulline; GLY, glycine; LEU, leucine; ILE, isoleucine; ALLO-ILE, alloisoleucine; PRO-OH, hydroxyproline; MET, methionine; ORN, ornithine; PHE, phenylalanine; PRO, proline; TYR, Tyrosine; VAL, valine; C0, free carnitine; C2, acetylcarnitine; C3, propionylcarnitine; C3-DC + C4-OH, malonylcarnitine + 3-hydroxybutyrylcarnitine; C4, butyrylcarnitine + isobutyrylcarnitine; C4-DC + C5-OH, methylmalonylcarnitine + 3-hydroxyisovalerylcarnitine; C5, isovalerylcarnitine + methylbutyrylcarnitine; C5-DC + C6-OH, glutarylcarnitine + 3-hydroxyhexanoylcarnitine; C6, hexanoylcarnitine; C6-DC, methylglutarylcarnitine; C8, octanoylcarnitine; C8:1, octenoylcarnitine; C10, decanoylcarnitine; C10:1, decenoylcarnitine; C12, dodecanoylcarnitine; C12:1, dodecenoylcarnitine; C14, tetradecanoylcarnitine; C14:1, tetradecenoylcarnitine; C16, palmitoylcarnitine; C16:1, palmitoleylcarnitine; C16:1-OH, 3-hydroxypalmitoleylcarnitine; C18, stearoylcarnitine; C18:1, oleoylcarnitine; C18:2, linoleoylcarnitine.

**Fig. 4:**
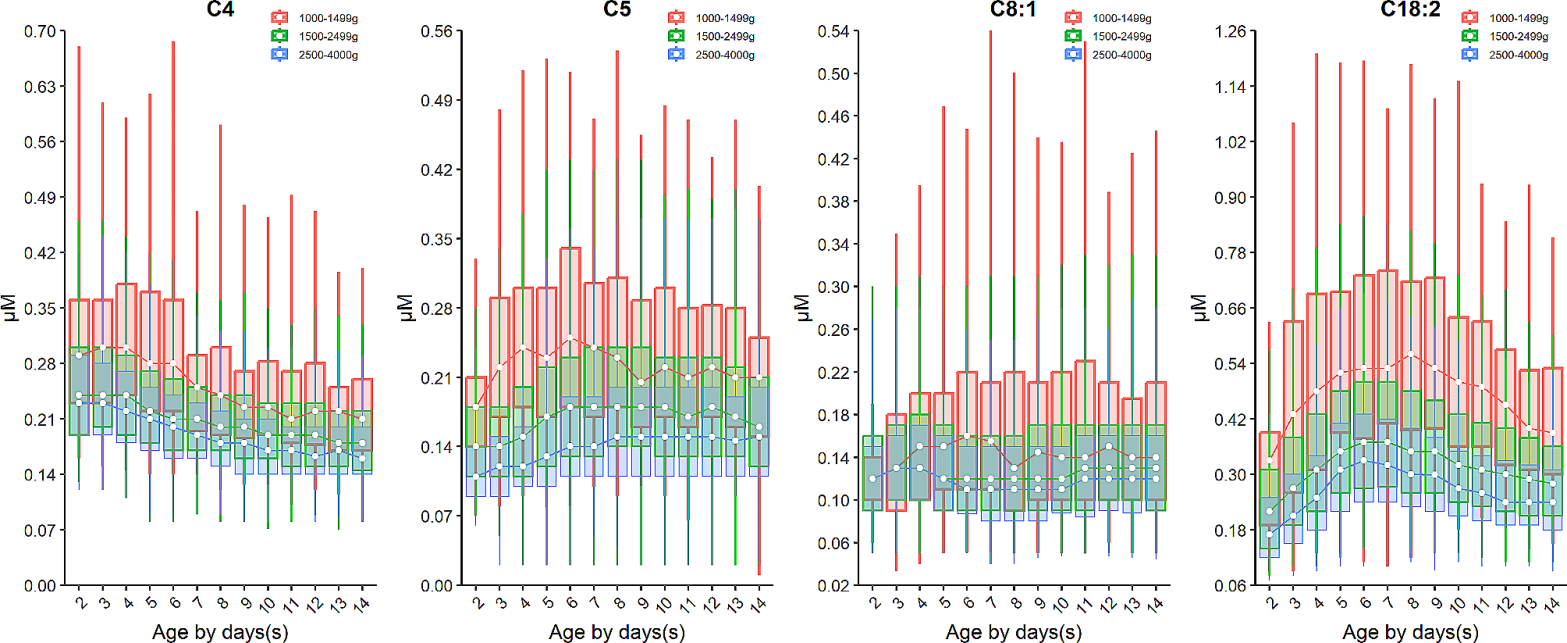
Dynamic change of acylcarnitines whose level decreased with the decreasing birth weight. Data of VLBW, LBW and NBW are shown in red, green and blue boxes with whiskers, respectively. The boxes extend from the 25th to the 75th percentile, with whiskers extending to the 2.5th or 97.5th percentile. Medians are shown as white circles in the body of boxes, and are linked with red (VLBW), green (LBW) or blue (NBW) line to shown the dynamics. Abbreviations are listed in the legend of Table 1

**Fig. 5:**
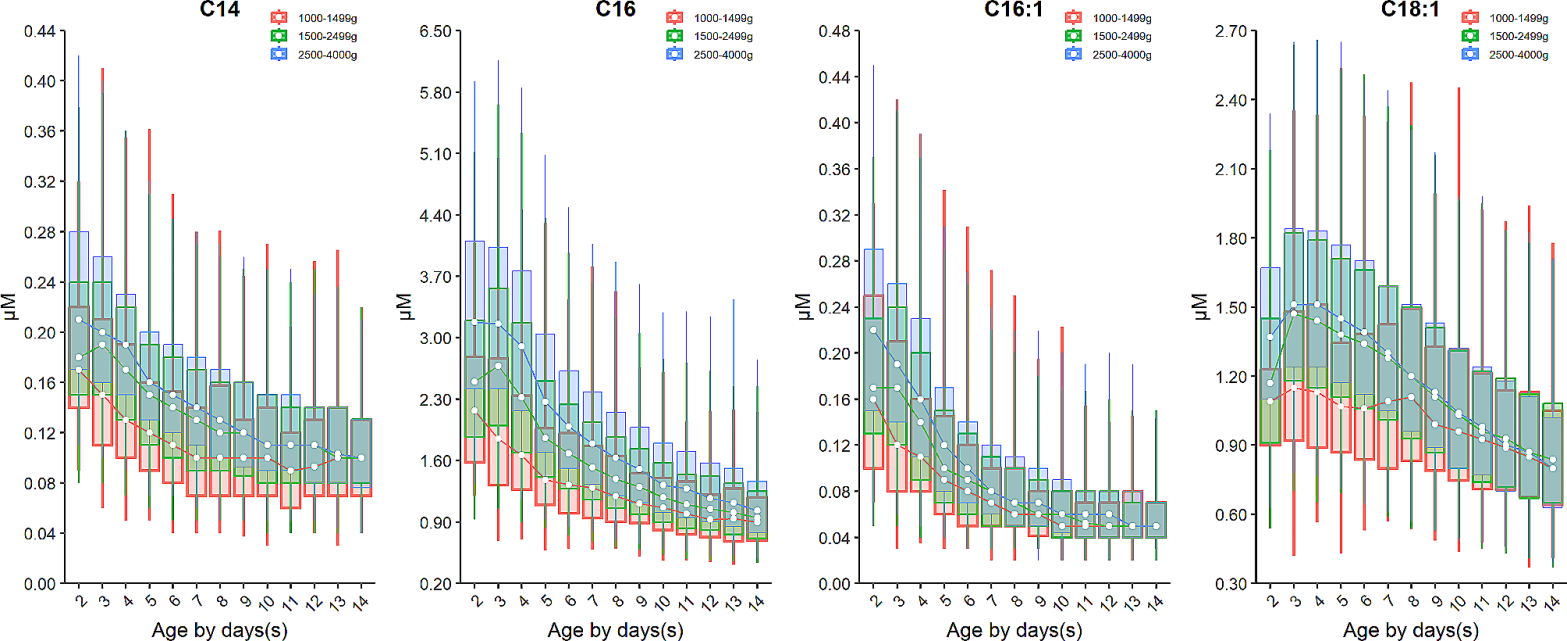
Dynamic change of acylcarnitines whose level increased with the increasing birth weight. Data of VLBW, LBW and NBW are shown in red, green and blue boxes with whiskers, respectively. The boxes extend from the 25th to the 75th percentile, with whiskers extending to the 2.5th or 97.5th percentile. Medians are shown as white circles in the body of boxes, and are linked with red (VLBW), green (LBW) or blue (NBW) line to shown the dynamics. Abbreviations are listed in the legend of Table 1

The turnover of acylcarnitines is also affected by the birth weight. For example, the median of short-chain acylcarnitine: butyrylcarnitine + sobutyrylcarnitine (C4) and isovalerylcarnitine + methylbutyrylcarnitine (C5) as well as a median-chain acylcarnitine octenoylcarnitine (C8:1) decrease over the birth weight (Fig. 4). On the contrary, most long-chain acylcarnitines such as tetradecanoylcarnitine (C14), palmitoylcarnitine (C16), palmitoleylcarnitine (C16:1) and oleoylcarnitine (C18:1) increased over the birth weight with the exception of linoleoylcarnitine (C18:2) whose level dropped over the birth weight (Fig. 5). Dozens of acylcarnitines including acetylcarnitine (C2), propionylcarnitine (C3), malonylcarnitine + 3-hydroxybutyrylcarnitine (C3-DC + C4-OH), butyrylcarnitine + isobutyrylcarnitine (C4), glutarylcarnitine + 3- hydroxyhexanoylcarnitine (C5-DC + C6-OH), hexanoylcarnitine (C6), methylglutarylcarnitine (C6-DC), decanoylcarnitine (C10), dodecanoylcarnitine (C12), dodecenoylcarnitine (C12:1), tetradecanoylcarnitine (C14), tetradecenoylcarnitine (C14:1), palmitoylcarnitine (C16), palmitoleylcarnitine (C16:1), and stearoylcarnitine (C18) presented overall downward trend over age. Whereas, free carnitine (C0), sovalerylcarnitine + methylbutyrylcarnitine (C5) and linoleoylcarnitine (C18:2) exhibited increasing concentrations with age (Figs. 4 and 5 & Supplementary Figure S1). Interestingly, the birth weight does not change the age-specific dynamic trends of any amino acids or acylcarnitines, as the group of VLBW, LBW and NBW showed a similar dynamic curve over age for any given analyte (Figs. 2, 3, 4 and 5, & Supplementary Figure S1). The results of inter-group differential analysis for various analytes are listed in Supplementary Table S1.

Unlike age and birth weight, sex affects only the level of malonylcarnitine + 3-hydroxybutyrylcarnitine (C3-DC + C4-OH). As indicated in Table 1, the Harris and Boyd method merely proposed the partition of C3-DC + C4-OH for the group of VLBW. The reference partition determined by Harris and Boyd method and the RIs for each analyte are listed in Table 1, Supplementary Table S2, & S3. Additional percentiles including the 0.5st, 25th, 50th, 75th, and 99.5th percentiles are reported in Supplementary Table S4, S5, & S6.

The performance of the RIs was evaluated using data from the NNSCP database, where the level of target biomarkers in 26 true-positives (15 phenylketonuria, 6 methylmalonic acidemia, 3 isovaleric acidemia, and 2 maple syrup urine disease), 39 false-positives (21 phenylketonuria, 7 propionic acidemia, 5 citrin deficiency, 4 hypermethioninemia, and 2 very long-chain acyl coenzyme A dehydrogenase deficiency) and 5 false-negatives of glutaric acidemia type I was compared to the reference ranges. The results revealed that the target biomarker level fell outside the RIs in 8 false-positives of phenylketonuria (8/21, 38.1%), 4 false-positives of propionic acidemia (4/7, 57.1%), 2 false-positives of citrin deficiency (2/5, 40.0%), 3 false-positives of hypermethioninemia (3/4, 75.0%), and 2 false positives of very long-chain acyl coenzyme A dehydrogenase deficiency (2/2, 100.0%). In contrast, the level of target biomarkers in all true-positives and false-negatives fell outside the RIs.

## Discussion

Currently, most NBS laboratories/centers in China do not have specific RIs for preterm neonates due to two reasons: first, the number of preterm infants received in a NBS laboratory/center is too few to develop comprehensive RIs; second, some pediatricians believe that there is no need to establish specific RIs for preterm babies because the commonly adopted RIs from full-term newborns have largely explained the test results of preterm neonates. Our results highlight the need to develop specific RIs for preterm neonates. The RIs we proposed from preterm babies are quite different from the RIs derived from full-term infants [7].

A previous report established MS/MS NBS RIs for low birth-weight infants using central 99.5% intervals as the reference standard. However, their investigation did not reveal the dynamic trends of biomarkers [13]. We have filled this gap by providing the time-coursed changes of NBS analytes over age. Additionally, our study underscores the impact of birth weight, age and sex on the level of MS/MS NBS biomarkers and developes robust RIs for three groups of preterm neonates: very low birth weight (VLBW), low birth weight (LBW), and normal birth weight (NBW).

It is noteworthy that the levels of alanine (ALA), proline (PRO), and tyrosine (TYR) decreased with increasing birth weight, while the levels of other tested amino acids tend to increase. Although the reason for this phenomenon is not clear, it is unlikely to be attributed to the gestational period. This conclusion is supported by a similar trend observed in a study that included the full-term babies [13]. Moreover, there was a decrease in the levels of short-chain acylcarnitines C4 and C5, as well as median-chain acylcarnitine C8:1 with increasing birth weight. Conversely, most long-chain acylcarnitines (C14, C16, C16:1, and C18:1) exhibited an increase in levels as birth weight increased. A recent study suggested that the levels of C4 and C5 were barely changed over the birth weight. The differences in outcomes between studies could be attributed to variations in the study population, as we focused on preterm neonates while other studies examined full-term babies [14].

In the case of full-term neonates, sex is an important cofactor affecting the levels of most MS/MS NBS biomarkers [7]. However, in preterm neonates with very low birth weight (1000 –1499 g), sex only impacts the level of C3-DC + C4-OH. The reason for this difference is still unclear, but several reports have highlighted that even preterm infants exhibit sex-specific differences in factors such as fat mass and distribution [15].

It is important to acknowledge the limitations of our study. We excluded infants with an extremely low birth weight (< 1000 g) because they are often given full parenteral nutrition (FPN), which can lead to increased concentrations of branched-chain amino acids in a NBS test [16]. Moreover, we did not provide the RIs for neonates at one day of age due to the scarcity of data in the NNSCP database. Our study did not address whether additional cofactors, such as ethnicity, environmental factors, diet, and altitude, would affect the profiles of MS/MS NBS biomarkers [17, 18]. Also, blood spot samples cannot replace cord blood or serum specimens to confirm the diagnosis of inborn errors, and the concentration of biomarkers outside of the RIs does not necessarily indicate the presence of disease.

## Conclusion

This study has developed robust reference intervals (RIs) for three groups of preterm neonates based on birth weight and has provided time-course changes of NBS analytes over age. Our results emphasize the impact of birth weight, age, and sex on the levels of MS/MS NBS biomarkers. Despite certain limitations, such as exclusion of infants with extremely low birth weight and the absence of consideration for additional cofactors that may affect the profiles of MS/MS NBS biomarkers, this study provides valuable insights into the development of specific RIs for preterm neonates.

### Electronic supplementary material

Below is the link to the electronic supplementary material.

Supplementary Material 1

## Data Availability

The data that support the findings of this study are available from the Ethics Committee of the Chinese PLA General Hospital but restrictions apply to the availability of these data, which were used under license for the current study, and so are not publicly available. Data are however available from the authors upon reasonable request and with permission of the Ethics Committee of the Chinese PLA General Hospital. Please try to contact tangtian12345@scu.edu.cn for further information and assistance regarding data access.
