# Supplementary material for: Retrospective analysis of reference intervals for dried blood spot based ms/ms newborn screening programs in Chinese preterm neonates: a nationwide study
Source: BMC Pediatr. 2024 Jul 2;24:424. doi: 10.1186/s12887-024-04865-1 (PMC11220950; doi:10.1186/s12887-024-04865-1)

Supplementary materials

Table S1. The median of analytes for different birth weight groups (μM)

Analytes	Median			*q* values		
	VLBW	LBW	NBW	VLBW/LBW	VLBW/NBW	LBW/NBW
ALA	254.41	282.02	289.61	< 0.001	< 0.001	< 0.001
ARG	17.10	12.55	10.65	< 0.001	< 0.001	< 0.001
CIT	15.70	14.30	12.98	< 0.001	< 0.001	< 0.001
GLY	455.07	442.34	466.93	< 0.001	< 0.001	< 0.001
LEU/ILE//PRO-OH	166.17	157.92	152.63	< 0.001	< 0.001	< 0.001
MET	23.475	20.49	19.11	< 0.001	< 0.001	< 0.001
ORN	123.315	116.44	111.95	< 0.001	< 0.001	< 0.001
PHE	63.19	57.94	55.18	< 0.001	< 0.001	< 0.001
PRO	168.09	175.84	182.41	< 0.001	< 0.001	< 0.001
TYR	86.75	109.51	117.32	< 0.001	< 0.001	< 0.001
VAL	141.35	133.095	130.55	< 0.001	< 0.001	< 0.001
C0	28.48	26.81	23.481	< 0.001	< 0.001	< 0.001
C2	15.35	16.27	18	< 0.001	< 0.001	< 0.001
C3	1.25	1.55	1.73	< 0.001	< 0.001	< 0.001
C3-DC+C4-OH	0.09	0.09	0.11	< 0.001	< 0.001	< 0.001
C4	0.25	0.22	0.22	< 0.001	< 0.001	< 0.001
C4-DC+C5-OH	0.18	0.17	0.18	< 0.001	> 0.1	< 0.001
C5	0.23	0.16	0.13	< 0.001	< 0.001	< 0.001
C5-DC+C6-OH	0.11	0.11	0.11	> 0.1	< 0.001	< 0.001
C6	0.04	0.04	0.04	< 0.001	< 0.001	< 0.001
C6-DC	0.08	0.09	0.09	< 0.001	< 0.001	< 0.001
C8	0.07	0.06	0.06	< 0.001	< 0.001	< 0.001
C8:1	0.15	0.13	0.13	< 0.001	< 0.001	< 0.001
C10	0.05	0.06	0.07	< 0.001	< 0.001	< 0.001
C10:1	0.06	0.06	0.07	< 0.05	< 0.001	< 0.001
C12	0.04	0.06	0.08	< 0.001	< 0.001	< 0.001
C12:1	0.03	0.04	0.05	< 0.001	< 0.001	< 0.001
C14	0.11	0.15	0.18	< 0.001	< 0.001	< 0.001
C14:1	0.05	0.06	0.08	< 0.001	< 0.001	< 0.001
C16	1.28	1.91	2.73	< 0.001	< 0.001	< 0.001
C16:1	0.08	0.11	0.15	< 0.001	< 0.001	< 0.001
C16:1-OH	0.03	0.03	0.03	< 0.001	< 0.001	< 0.001
C18	0.65	0.69	0.81	< 0.001	< 0.001	< 0.001
C18:1	1.03	1.31	1.45	< 0.001	< 0.001	< 0.001
C18:2	0.48	0.32	0.25	< 0.001	< 0.001	< 0.001

Abbreviation: VLBW, very low birth weight; LBW, low birth weight; NBW, normal birth weight

Table S2. RIs for 35 MS/MS NBS biomarkers in preterm neonates of 1500g-2499g (μM)

Analytes	Age	Lower limit (2.5th) and 90% CI	Upper limit (97.5th) and 90% CI	No. of samples
		Amino acids		
ALA	2 days to ≤ 14 days	152.1 (151.3-152.8)	509.2 (506.4-511.5)	75276
ARG	2 days to ≤ 14 days	2.4 (2.3-2.4)	49.4 (49.0-49.8)	75715
CIT	2 days to ≤ 14 days	7.9 (7.9-8.0)	27.1 (27.0-27.3)	75558
GLY	2 days to ≤ 14 days	255.4 (254.2-256.4)	851.5 (847.7-856.3)	75620
LEU/ILE/ ALLO-ILE/PRO-OH	2 days to ≤ 14 days	86.9 (86.4-87.2)	286.9 (285.4-288.0)	75562
MET	2 days to ≤ 14 days	10.0 (9.9-10.0)	43.3 (43.1-43.5)	75573
ORN	2 days to ≤ 14 days	52.4 (52.1-52.7)	274.8 (273.0-277.2)	75637
PHE	2 days to ≤ 14 days	35.3 (35.2-35.5)	100.4 (100.1-100.9)	75603
PRO	2 days to ≤ 14 days	99.1 (98.8-99.5)	300.3 (298.8-301.3)	75064
TYR	2 days to ≤ 14 days	50.4 (50.1-50.6)	277.0 (275.2-280.0)	74202
VAL	2 days to ≤ 14 days	69.2 (68.7-69.7)	235.0 (234.1-235.6)	75362
		Acylcarnitnes		
C0	2 days to ≤ 14 days	13.80 (13.75-13.85)	53.08 (52.70-53.36)	75411
C2	2 days to ≤ 5 days	9.17 (9.12-9.23)	39.79 (39.50-40.07)	45318
	5 days to ≤ 14 days	5.66 (5.61-5.71)	25.85 (25.59-26.09)	29722
C3	2 days to ≤ 5 days	0.85 (0.84-0.86)	4.43 (4.40-4.47)	45593
	5 days to ≤ 6 days	0.65 (0.64-0.66)	3.26 (3.18-3.34)	5736
	6 days to ≤ 14 days	0.42 (0.42-0.43)	2.32 (2.28-2.35)	24195
C3-DC+C4-OH	2 days to ≤ 14 days	0.04 (0.04-0.04)	0.27 (0.27-0.28)	74994
C4	2 days to ≤ 14 days	0.12 (0.12-0.12)	0.40 (0.40-0.40)	73659
C4-DC+C5-OH	2 days to ≤ 14 days	0.10 (0.10-0.10)	0.31 (0.30-0.31)	75180
C5	2 days to ≤ 14 days	0.07 (0.07-0.07)	0.36 (0.35-0.36)	72714
C5-DC+C6-OH	2 days to ≤ 14 days	0.05 (0.05-0.05)	0.22 (0.22-0.22)	75632
C6	2 days to ≤ 14 days	0.02 (0.02-0.02)	0.09 (0.09-0.09)	74485
C6-DC	2 days to ≤ 14 days	0.04 (0.04-0.04)	0.21 (0.21-0.21)	75788
C8	2 days to ≤ 14 days	0.03 (0.03-0.03)	0.13 (0.13-0.13)	75638
C8:1	2 days to ≤ 14 days	0.06 (0.05-0.06)	0.30 (0.30-0.31)	74918
C10	2 days to ≤ 14 days	0.03 (0.03-0.03)	0.17 (0.17-0.17)	74859
C10:1	2 days to ≤ 14 days	0.03 (0.03-0.03)	015 (0.15-0.15)	75382
C12	2 days to ≤ 4 days	0.03 (0.03-0.03)	0.21 (0.21-0.22)	37810
	4 days to ≤ 14 days	0.02 (0.02-0.02)	0.11 (0.11-0.11)	36394
C12:1	2 days to ≤ 5 days	0.01 (0.01-0.01)	0.17 (0.17-0.17)	45951
	5 days to ≤ 14 days	0.01 (0.01-0.01)	0.09 (0.09-0.10)	30160
C14	2 days to ≤ 4 days	0.08 (0.08-0.08)	0.37 (0.37-0.37)	38146
	4 days to ≤ 14 days	0.05 (0.05-0.05)	0.27 (0.27-0.27)	36926
C14:1	2 days to ≤ 3 days	0.04 (0.04-0.04)	0.23 (0.23-0.23)	18916
	3 days to ≤ 4 days	0.03 (0.03-0.03)	0.19 (0.19-0.19)	19437
	4 days to ≤ 14 days	0.02 (0.02-0.02)	0.10 (0.10-0.10)	36621
C16	2 days to ≤ 4 days	1.03 (1.02-1.04)	5.50 (5.46-5.53)	38486
	4 days to ≤ 8 days	0.76 (0.75-0.77)	3.96 (3.92-4.04)	21202
	8 days to ≤ 14 days	0.52 (0.52-0.53)	2.73 (2.69-2.78)	15773
C16:1	2 days to ≤ 4 days	0.05 (0.05-0.05)	0.39 (0.39-0.40)	38364
	4 days to ≤ 6 days	0.04 (0.04-0.04)	0.28 (0.28-0.29)	12862
	6 days to ≤ 14 days	0.03 (0.03-0.03)	0.18 (0.18-0.18)	24089
C16:1-OH	2 days to ≤ 3 days	0.03 (0.03-0.03)	0.06 (0.06-0.06)	15218
	3 days to ≤ 14 days	0.02 (0.02-0.02)	0.05 (0.05-0.05)	52043
C18	2 days to ≤ 9 days	0.36 (0.36-0.36)	1.45 (1.44-1.45)	62948
	9 days to ≤ 14 days	0.25 (0.24-0.25)	0.99 (0.98-1.01)	12539
C18:1	2 days to ≤ 10 days	0.66 (0.65-0.66)	2.46 (2.45-2.47)	65445
	10 days to ≤ 14 days	0.44 (0.43-0.44)	1.77 (1.74-1.79)	9500
C18:2	2 days to ≤ 14 days	0.12 (0.12-0.12)	0.76 (0.76-0.77)	75136

Abbreviation: ALA, alanine; ARG, arginine; CIT, citrulline; GLY, glycine; LEU, leucine; ILE, isoleucine; ALLO-ILE, alloisoleucine; PRO-OH, hydroxyproline; MET, methionine; ORN, ornithine; PHE, phenylalanine; PRO, proline; TYR, Tyrosine; VAL, valine; C0, free carnitine; C2, acetylcarnitine; C3, propionylcarnitine; C3-DC+C4-OH, malonylcarnitine+3-hydroxybutyrylcarnitine; C4, butyrylcarnitine+isobutyrylcarnitine; C4-DC+C5-OH, methylmalonylcarnitine+3-hydroxyisovalerylcarnitine; C5, isovalerylcarnitine+methylbutyrylcarnitine; C5-DC+C6-OH, glutarylcarnitine+3-hydroxyhexanoylcarnitine; C6, hexanoylcarnitine; C6-DC, methylglutarylcarnitine; C8, octanoylcarnitine; C8:1, octenoylcarnitine; C10, decanoylcarnitine; C10:1, decenoylcarnitine; C12, dodecanoylcarnitine; C12:1, dodecenoylcarnitine; C14, tetradecanoylcarnitine; C14:1, tetradecenoylcarnitine; C16, palmitoylcarnitine; C16:1, palmitoleylcarnitine; C16:1-OH, 3-hydroxypalmitoleylcarnitine; C18, stearoylcarnitine; C18:1, oleoylcarnitine; C18:2, linoleoylcarnitine.

Table S3. RIs for 35 MS/MS NBS biomarkers in neonates of 2500g-3999g (μM)				
**Analytes**	**Age**	**Lower limit (2.5th) and 90% CI**	**Upper limit (97.5th) and 90% CI**	**No. of samples**
		Amino acids		
ALA	2 days to ≤ 14 days	164.8(164.3-165.3)	521.4(519.5-523.6)	116585
ARG	2 days to ≤ 14 days	1.94(1.93-1.96)	37.9(37.6-38.1)	117074
CIT	2 days to ≤ 14 days	7.6(7.6-7.7)	23.4(23.3-23.5)	116592
GLY	2 days to ≤ 14 days	266.6(265.8-267.5)	865.6(862.7-869.0)	116584
LEU/ILE/ ALLO-ILE/PRO-OH	2 days to ≤ 14 days	91.9(91.6-92.1)	256.0(255.1-256.9)	116616
MET	2 days to ≤ 14 days	9.9(9.8-9.9)	36.6(36.4-36.8)	116685
ORN	2 days to ≤ 14 days	56.6(56.3-56.8)	256.0(254.4-257.4)	116636
PHE	2 days to ≤ 14 days	35.8(35.7-35.9)	88.7(88.4-89.0)	116568
PRO	2 days to ≤ 14 days	112.5(112.2-112.8)	308.3(307.3-309.5)	116138
TYR	2 days to ≤ 14 days	56.1(55.8-56.3)	280.6(279.0-282.2)	115358
VAL	2 days to ≤ 14 days	74.4(74.0-74.7)	213.5(213.0-213.9)	115260
		Acylcarnitnes		
C0	2 days to ≤ 14 days	12.53(12.48-12.57)	46.34(46.14-46.55)	116551
C2	2 days to ≤ 6 days	9.20(9.16-9.25)	37.37(37.27-37.55)	98794
	6 days to ≤ 14 days	5.52(5.45-5.59)	24.56(24.18-24.86)	16998
C3	2 days to ≤ 6 days	0.85(0.85-0.85)	4.13(4.12-4.16)	99429
	6 days to ≤ 14 days	0.41(0.41-0.42)	2.42(2.37-2.46)	17209
C3-DC+C4-OH	2 days to ≤ 14 days	0.04(0.04-0.04)	0.33(0.33-0.33)	117054
C4	2 days to ≤ 14 days	0.12(0.12-0.12)	0.40(0.40-0.40)	114793
C4-DC+C5-OH	2 days to ≤ 14 days	0.10(0.10-0.10)	0.32(0.32-0.32)	115925
C5	2 days to ≤ 14 days	0.07(0.06-0.07)	0.26(0.26-0.26)	111432
C5-DC+C6-OH	2 days to ≤ 14 days	0.06(0.06-0.06)	0.23(0.23-0.23)	116937
C6	2 days to ≤ 6 days	0.02(0.02-0.02)	0.08(0.08-0.09)	97087
	6 days to ≤ 7 days	0.03(0.03-0.03)	0.06(0.06-0.06)	2963
	7 days to ≤ 14 days	0.02(0.02-0.02)	0.06(0.06-0.06)	12278
C6-DC	2 days to ≤ 14 days	0.04(0.04-0.04)	0.23(0.22-0.23)	116828
C8	2 days to ≤ 14 days	0.03(0.03-0.03)	0.12(0.12-0.12)	115893
C8:1	2 days to ≤ 14 days	0.06(0.06-0.06)	0.27(0.27-0.27)	115224
C10	2 days to ≤ 14 days	0.03(0.03-0.03)	0.19(0.19-0.19)	115695
C10:1	2 days to ≤ 14 days	0.03(0.03-0.03)	0.15(0.15-0.15)	116114
C12	2 days to ≤ 4 days	0.04(0.04-0.04)	0.24(0.24-0.24)	85426
	4 days to ≤ 14 days	0.02(0.02-0.02)	0.12(0.12-0.12)	29250
C12:1	2 days to ≤ 14 days	0.02(0.02-0.02)	0.18(0.18-0.18)	116030
C14	2 days to ≤ 14 days	0.08(0.08-0.08)	0.36(0.36-0.37)	116069
C14:1	2 days to ≤ 4 days	0.04(0.04-0.04)	0.23(0.23-0.23)	86114
	4 days to ≤ 6 days	0.03(0.03-0.03)	0.13(0.12-0.13)	12687
	6 days to ≤ 14 days	0.03(0.03-0.03)	0.09(0.09-0.09)	16422
C16	2 days to ≤ 5 days	1.28(1.27-1.28)	5.95(5.92-5.97)	94286
	5 days to ≤ 9 days	0.81(0.79-0.82)	4.15(4.09-4.21)	14407
	9 days to ≤ 14 days	0.57(0.56-0.58)	3.22(3.12-3.29)	7940
C16:1	2 days to ≤ 4 days	0.06(0.06-0.06)	0.41(0.40-0.41)	86248
	4 days to ≤ 6 days	0.04(0.04-0.04)	0.30(0.29--0.30)	12856
	6 days to ≤ 14 days	0.03(0.03-0.03)	0.21(0.20-0.21)	17021
C16:1-OH	2 days to ≤ 3 days	0.03(0.03-0.03)	0.06(0.06-0.06)	37332
	3 days to ≤ 14 days	0.02(0.02-0.02)	0.06(0.05-0.06)	67916
C18	2 days to ≤ 14 days	0.37(0.37-0.37)	1.56(1.56-1.57)	116615
C18:1	2 days to ≤ 13 days	0.71(0.71-0.72)	2.46(2.45-2.47)	113547
	13 days to ≤ 14 days	0.41(0.39-0.43)	1.55(1.53-1.61)	1264
C18:2	2 days to ≤ 14 days	0.09(0.09-0.09)	0.57(0.57-0.58)	116076

Abbreviation: ALA, alanine; ARG, arginine; CIT, citrulline; GLY, glycine; LEU, leucine; ILE, isoleucine; ALLO-ILE, alloisoleucine; PRO-OH, hydroxyproline; MET, methionine; ORN, ornithine; PHE, phenylalanine; PRO, proline; TYR, Tyrosine; VAL, valine; C0, free carnitine; C2, acetylcarnitine; C3, propionylcarnitine; C3-DC+C4-OH, malonylcarnitine+3-hydroxybutyrylcarnitine; C4, butyrylcarnitine+isobutyrylcarnitine; C4-DC+C5-OH, methylmalonylcarnitine+3-hydroxyisovalerylcarnitine; C5, isovalerylcarnitine+methylbutyrylcarnitine; C5-DC+C6-OH, glutarylcarnitine+3-hydroxyhexanoylcarnitine; C6, hexanoylcarnitine; C6-DC, methylglutarylcarnitine; C8, octanoylcarnitine; C8:1, octenoylcarnitine; C10, decanoylcarnitine; C10:1, decenoylcarnitine; C12, dodecanoylcarnitine; C12:1, dodecenoylcarnitine; C14, tetradecanoylcarnitine; C14:1, tetradecenoylcarnitine; C16, palmitoylcarnitine; C16:1, palmitoleylcarnitine; C16:1-OH, 3-hydroxypalmitoleylcarnitine; C18, stearoylcarnitine; C18:1, oleoylcarnitine; C18:2, linoleoylcarnitine.

Table S4. The 0.5th, 2.5th, 25th, 50th, 75th ,97.5thand 99.5th percentiles calculated by age and sex for 35 MS/MS NBS biomarkers for the preterm neonates of 1000g-1499g (μM)

		Amino acids													
		Male							Female						
**Analytes**	**Age**	**0.5th**	**2.5th**	**25th**	**50th**	**75th**	**97.5th**	**99.5th**	**0.5th**	**2.5th**	**25th**	**50th**	**75th**	**97.5th**	**99.5th**
ALA	2 days to ≤ 3 days	118.7	134.0	193.8	248.9	339.8	513.1	570.0	132.0	141.1	201.4	257.4	343.5	528.3	644.6
	3 days to ≤ 4 days	102.0	118.7	198.0	252.0	315.6	460.6	532.4	118.0	127.5	199.4	264.7	347.3	545.5	597.8
	4 days to ≤ 5 days	106.0	117.9	198.9	232.8	287.3	409.4	435.9	131.9	136.6	206.8	255.0	337.1	513.1	582.7
	5 days to ≤ 6 days	124.6	131.2	176.3	235.6	331.2	493.9	548.9	136.2	157.2	196.3	256.7	315.4	476.5	534.7
	6 days to ≤ 7 days	144.8	151.8	213.7	257.4	311.7	538.3	559.7	147.1	158.0	212.5	243.0	287.7	397.3	455.7
	7 days to ≤ 8 days	164.6	173.0	220.8	262.5	318.3	471.0	621.5	129.8	141.3	202.3	247.0	321.7	475.9	552.6
	8 days to ≤ 9 days	90.2	117.1	200.9	237.5	279.0	374.1	395.7	159.0	167.7	227.0	257.5	306.1	467.4	523.9
	9 days to ≤ 10 days	141.0	143.7	216.8	270.7	326.2	473.0	638.4	133.9	142.2	201.2	262.1	348.8	501.4	584.0
	10 days to ≤ 11 days	137.8	147.9	197.7	241.1	294.3	589.6	657.6	126.0	155.0	233.3	281.2	360.9	493.2	509.7
	11 days to ≤ 12 days	143.0	149.8	199.1	243.4	308.8	458.8	599.1	158.7	167.7	223.5	279.1	333.0	434.2	494.4
	12 days to ≤ 13 days	147.7	160.4	222.3	253.2	329.5	498.3	557.6	115.2	134.5	207.6	246.4	300.8	407.0	456.6
	13 days to ≤ 14 days	157.2	164.4	230.0	266.0	329.4	459.6	511.4	135.9	149.1	207.6	271.0	336.9	490.5	544.5
ARG	2 days to ≤ 3 days	2.4	3.1	9.5	17.9	30.4	57.4	67.5	1.8	2.9	9.3	16.3	25.6	52.6	71.9
	3 days to ≤ 4 days	2.3	3.0	8.2	14.2	25.7	53.2	61.1	3.0	3.5	8.6	14.3	22.9	55.2	63.0
	4 days to ≤ 5 days	3.1	3.6	9.5	16.1	30.8	60.2	66.4	2.5	2.7	9.3	15.4	27.2	53.5	64.1
	5 days to ≤ 6 days	3.2	3.5	11.1	19.9	31.5	58.6	66.5	2.9	3.2	9.6	17.9	26.5	45.6	50.0
	6 days to ≤ 7 days	1.9	2.5	8.4	16.4	30.8	51.6	64.5	2.1	2.5	10.5	16.7	30.9	49.7	55.5
	7 days to ≤ 8 days	2.7	3.5	10.4	16.7	29.6	60.0	72.4	3.0	3.4	10.4	15.7	26.2	53.8	60.2
	8 days to ≤ 9 days	2.6	4.8	9.8	20.1	32.6	61.3	70.8	4.0	4.5	10.6	18.0	27.1	47.8	60.8
	9 days to ≤ 10 days	4.0	4.4	13.3	19.7	36.3	62.5	67.9	2.9	3.9	9.2	18.9	37.0	62.1	82.5
	10 days to ≤ 11 days	4.5	5.2	11.4	18.9	27.0	60.5	85.9	4.7	5.1	12.5	21.6	33.3	65.8	72.6
	11 days to ≤ 12 days	3.3	3.7	12.0	18.9	31.4	54.9	78.4	2.6	3.9	10.2	17.8	28.4	59.5	66.7
	12 days to ≤ 13 days	4.0	4.8	12.5	17.9	28.4	54.0	73.0	1.7	2.6	9.1	16.9	28.9	63.0	69.1
	13 days to ≤ 14 days	2.5	3.5	11.5	22.1	38.4	72.9	78.4	2.7	3.1	9.7	16.3	26.0	46.4	73.4
CIT	2 days to ≤ 3 days	7.3	8.2	12.1	15.0	20.5	30.3	33.0	7.0	7.4	12.2	15.3	18.9	31.5	34.9
	3 days to ≤ 4 days	8.1	8.3	12.3	16.1	19.2	28.2	32.4	8.2	9.4	12.7	15.7	19.4	29.8	40.2
	4 days to ≤ 5 days	7.6	8.3	12.5	15.0	19.0	31.0	33.9	7.1	7.9	12.3	15.6	20.2	32.0	38.4
	5 days to ≤ 6 days	6.7	7.0	12.4	17.0	20.5	31.2	37.1	8.5	9.3	13.4	16.2	21.4	30.8	41.6
	6 days to ≤ 7 days	7.8	8.7	12.5	15.2	20.9	28.2	34.0	7.9	8.5	12.0	15.0	20.8	29.7	38.1
	7 days to ≤ 8 days	7.7	8.8	12.6	15.0	19.2	29.4	32.3	7.3	7.8	12.2	15.4	19.9	35.3	43.3
	8 days to ≤ 9 days	6.8	7.4	12.2	15.5	20.5	36.6	42.2	6.5	7.1	11.5	13.7	18.8	32.3	39.5
	9 days to ≤ 10 days	7.9	8.2	13.1	15.7	18.9	30.2	32.3	6.6	7.7	12.8	15.7	21.0	30.5	33.5
	10 days to ≤ 11 days	7.5	8.5	12.4	15.5	18.1	27.6	29.5	6.8	8.3	13.1	16.3	21.1	29.8	33.0
	11 days to ≤ 12 days	7.0	8.3	12.5	15.8	19.8	30.5	31.9	8.9	9.4	13.5	16.9	21.0	31.8	41.4
	12 days to ≤ 13 days	6.6	7.3	11.6	16.1	19.5	28.6	30.9	6.7	8.2	12.6	14.4	18.8	27.7	29.6
	13 days to ≤ 14 days	7.7	8.6	12.4	17.0	21.7	31.8	34.2	8.3	9.1	13.1	16.3	21.9	33.1	37.1
GLY	2 days to ≤ 3 days	241.8	271.1	408.6	509.5	660.2	898.5	1002.5	270.2	291.9	410.3	479.3	619.7	928.1	1087.5
	3 days to ≤ 4 days	259.7	276.6	401.7	492.6	590.0	888.2	961.2	256.8	273.3	416.0	516.3	635.7	885.9	1038.4
	4 days to ≤ 5 days	251.6	271.3	375.2	469.9	553.2	829.2	958.0	288.3	317.3	387.4	466.7	530.2	733.9	814.0

	5 days to ≤ 6 days	240.9	264.0	349.7	422.9	541.2	848.0	950.0	265.1	289.0	359.5	452.0	566.2	922.4	1037.1
	6 days to ≤ 7 days	244.6	280.1	367.8	436.3	525.9	822.1	900.2	291.2	316.8	383.3	428.8	506.6	729.2	810.4
	7 days to ≤ 8 days	197.2	239.5	362.4	429.1	505.9	691.3	728.1	235.2	239.5	335.9	431.7	539.8	725.0	856.1
	8 days to ≤ 9 days	224.6	252.4	342.3	418.9	481.2	697.6	782.5	199.9	246.0	342.7	423.7	507.5	633.4	646.9
	9 days to ≤ 10 days	257.6	283.7	375.6	438.1	497.4	825.0	884.4	228.5	258.3	377.8	444.2	550.0	851.8	916.8
	10 days to ≤ 11 days	220.3	238.3	355.0	461.9	526.2	782.2	823.6	275.9	294.7	402.6	475.9	552.3	764.5	806.7
	11 days to ≤ 12 days	240.8	268.9	349.9	420.1	502.2	772.6	811.8	217.1	250.9	366.0	440.2	547.5	799.7	853.1
	12 days to ≤ 13 days	227.7	250.2	354.9	432.8	544.6	727.0	804.8	242.9	259.0	354.7	397.8	480.5	641.2	676.8
	13 days to ≤ 14 days	247.8	262.4	369.8	440.7	542.3	692.2	796.8	220.2	240.3	336.5	410.4	522.7	787.9	846.9
LEU/ILE/ALLO-ILE/PRO-OH	2 days to ≤ 3 days	50.1	62.0	117.0	163.3	214.6	305.6	372.6	46.9	66.7	110.9	157.6	207.2	279.9	366.6
	3 days to ≤ 4 days	50.7	66.5	121.9	162.7	212.0	301.9	339.7	60.2	76.2	124.5	167.9	211.0	314.0	373.5
	4 days to ≤ 5 days	57.9	69.9	110.9	159.3	207.2	306.8	332.3	64.9	69.8	121.5	176.4	208.0	293.8	317.0
	5 days to ≤ 6 days	67.1	75.6	127.5	175.3	220.4	299.1	359.3	80.1	85.1	127.8	166.1	215.7	310.6	337.4
	6 days to ≤ 7 days	64.5	80.0	129.8	178.1	209.1	283.8	299.1	74.2	81.0	133.3	178.1	220.7	305.3	357.5
	7 days to ≤ 8 days	78.9	90.8	134.8	164.0	201.6	299.1	306.7	49.3	80.7	143.0	169.6	202.9	272.6	278.2
	8 days to ≤ 9 days	75.0	77.4	125.2	162.6	208.8	280.1	348.5	65.5	80.4	140.0	179.7	208.7	280.3	296.1
	9 days to ≤ 10 days	81.9	82.3	133.1	172.4	221.8	305.0	329.8	73.7	91.8	129.4	169.3	228.2	326.7	425.8
	10 days to ≤ 11 days	84.8	99.4	130.4	167.6	209.7	314.5	420.6	89.1	99.3	139.5	178.4	212.4	293.1	321.5
	11 days to ≤ 12 days	96.3	101.7	144.8	164.3	194.7	283.0	309.8	80.7	97.5	139.9	166.8	209.6	289.5	317.2
	12 days to ≤ 13 days	85.0	102.5	129.7	157.0	206.8	283.6	327.3	84.7	87.5	129.5	154.7	193.6	305.2	347.2
	13 days to ≤ 14 days	96.7	101.2	144.1	177.3	213.3	285.8	333.5	90.7	98.0	130.6	165.4	201.0	292.4	319.4
MET	2 days to ≤ 3 days	9.6	11.4	16.4	22.1	30.9	54.3	65.7	9.7	10.6	16.8	21.6	29.1	50.8	56.7
	3 days to ≤ 4 days	6.8	8.6	15.6	22.0	30.1	48.8	53.9	7.6	9.7	17.7	21.9	30.6	48.3	57.8
	4 days to ≤ 5 days	7.2	10.6	16.9	22.1	31.8	48.5	58.5	9.0	9.8	18.6	24.0	30.6	44.4	49.4
	5 days to ≤ 6 days	10.1	10.7	17.5	24.3	32.5	48.9	58.9	10.2	12.0	17.7	23.2	31.6	47.5	56.7
	6 days to ≤ 7 days	8.2	10.0	18.7	25.9	32.9	44.9	47.5	11.1	13.3	20.3	26.3	31.9	51.3	54.8
	7 days to ≤ 8 days	9.8	10.9	17.9	24.4	31.6	49.6	61.3	8.9	10.0	17.1	22.6	30.1	45.3	48.9
	8 days to ≤ 9 days	9.0	9.5	18.4	23.6	32.8	45.5	51.3	9.5	11.1	16.9	24.8	33.3	46.5	47.6
	9 days to ≤ 10 days	10.8	11.4	19.1	25.4	32.6	51.9	58.9	10.8	12.5	18.4	24.6	32.8	53.0	61.2
	10 days to ≤ 11 days	9.8	10.5	19.5	23.9	32.3	59.5	71.0	12.2	12.6	20.5	25.3	32.6	50.4	56.7
	11 days to ≤ 12 days	10.0	11.3	17.6	23.7	30.8	42.7	50.7	8.8	9.7	19.7	25.2	34.9	51.5	66.8
	12 days to ≤ 13 days	8.2	9.5	17.8	23.7	34.4	52.3	60.0	10.4	11.3	16.7	19.9	31.7	54.7	60.9
	13 days to ≤ 14 days	13.8	14.7	19.7	26.2	34.8	59.2	67.1	8.3	9.7	15.8	23.2	30.4	46.7	59.6
ORN	2 days to ≤ 3 days	34.2	48.7	84.6	115.5	181.4	295.8	391.4	31.6	35.7	82.7	116.9	159.7	306.3	328.7
	3 days to ≤ 4 days	28.8	40.9	83.4	125.8	168.3	251.8	297.5	34.4	49.8	83.0	118.9	162.7	287.5	381.7
	4 days to ≤ 5 days	35.8	42.3	83.4	109.8	168.6	260.3	349.2	61.6	63.3	103.2	126.8	166.8	321.9	362.6
	5 days to ≤ 6 days	34.9	37.7	82.1	117.6	160.8	310.6	357.4	56.2	62.2	95.8	125.1	167.4	255.8	340.6
	6 days to ≤ 7 days	53.4	56.1	99.0	124.5	161.4	311.4	330.3	53.7	55.7	90.8	111.2	149.8	267.1	419.6
	7 days to ≤ 8 days	54.0	63.7	96.2	118.4	161.4	252.5	265.6	54.8	58.0	96.3	121.4	146.3	228.0	257.9
	8 days to ≤ 9 days	43.5	50.3	98.8	127.5	155.7	225.5	273.3	52.5	53.2	96.1	123.6	155.1	220.8	236.0
	9 days to ≤ 10 days	56.6	64.8	104.4	124.1	155.7	228.7	243.7	55.2	65.2	96.2	138.8	181.2	270.0	294.8
	10 days to ≤ 11 days	54.4	59.9	91.2	128.0	162.4	277.9	288.9	58.2	61.1	106.2	133.9	167.6	248.6	293.1
	11 days to ≤ 12 days	42.6	51.5	93.4	122.2	164.9	251.5	296.6	51.7	58.2	94.6	121.6	170.4	278.3	318.1
	12 days to ≤ 13 days	57.3	60.4	92.0	131.0	163.4	261.6	322.6	56.4	62.0	85.8	118.5	154.3	223.0	277.6

	13 days to ≤ 14 days	54.8	65.3	105.8	139.2	167.2	224.3	247.9	51.2	58.1	93.0	124.4	154.1	238.8	342.7
PHE	2 days to ≤ 3 days	30.4	36.2	54.0	70.5	86.1	116.8	119.0	35.8	40.4	56.2	67.0	79.6	114.9	122.1
	3 days to ≤ 4 days	32.7	36.8	56.3	69.0	82.6	109.7	119.4	29.6	39.4	53.5	69.2	84.7	109.7	123.5
	4 days to ≤ 5 days	37.3	38.0	55.1	65.7	75.6	108.2	118.1	31.8	32.9	51.9	64.0	78.1	110.5	122.7
	5 days to ≤ 6 days	38.7	41.0	52.0	64.9	78.2	107.9	110.1	35.8	39.7	53.1	64.6	77.3	101.3	115.9
	6 days to ≤ 7 days	39.0	40.9	56.8	68.1	79.8	112.1	117.8	38.5	40.9	53.1	65.0	75.9	99.2	107.0
	7 days to ≤ 8 days	36.5	40.2	52.6	60.4	71.3	97.4	103.6	26.1	38.3	50.1	58.0	70.5	91.2	93.1
	8 days to ≤ 9 days	33.5	34.5	50.0	62.4	70.2	94.0	102.5	31.8	33.3	50.3	61.3	72.3	94.6	99.8
	9 days to ≤ 10 days	31.1	33.3	50.3	61.2	72.8	95.4	108.1	34.8	35.7	50.8	57.5	71.7	105.5	112.0
	10 days to ≤ 11 days	34.7	36.0	50.1	57.2	75.9	103.3	113.4	37.9	40.3	51.1	63.3	74.5	110.7	116.1
	11 days to ≤ 12 days	35.1	37.7	51.0	59.0	72.4	95.9	137.9	32.6	34.2	48.8	56.0	69.4	105.5	117.8
	12 days to ≤ 13 days	34.6	36.1	49.2	58.7	72.9	100.7	119.9	33.1	33.4	44.9	53.0	66.2	95.6	103.9
	13 days to ≤ 14 days	31.2	38.6	49.8	62.4	72.5	99.6	115.9	35.4	37.0	46.3	54.4	69.4	94.4	104.0
PRO	2 days to ≤ 3 days	66.9	70.9	118.0	147.9	199.5	317.4	335.6	69.2	75.5	118.3	154.4	190.1	312.0	378.5
	3 days to ≤ 4 days	63.0	74.0	123.6	156.8	193.1	279.8	308.9	63.5	80.0	128.9	165.3	210.3	349.1	407.3
	4 days to ≤ 5 days	57.2	76.2	123.8	156.6	207.2	328.0	347.5	81.4	93.7	126.1	169.4	200.2	303.2	329.8
	5 days to ≤ 6 days	57.0	66.9	132.0	167.8	222.5	309.2	315.4	84.3	87.9	135.5	165.8	209.8	282.7	297.7
	6 days to ≤ 7 days	95.3	98.6	138.9	161.0	190.9	299.2	317.6	93.5	96.3	141.4	175.7	208.3	293.0	326.5
	7 days to ≤ 8 days	91.2	108.5	145.7	173.7	206.3	289.7	307.7	89.9	98.9	141.9	174.2	202.4	260.2	330.7
	8 days to ≤ 9 days	95.3	102.0	142.3	166.2	201.4	297.0	341.2	78.6	97.0	147.5	175.3	207.6	277.6	302.4
	9 days to ≤ 10 days	87.8	90.7	140.1	176.3	221.8	325.6	380.4	98.1	107.7	149.6	183.1	227.6	384.8	438.5
	10 days to ≤ 11 days	96.9	110.6	138.8	168.6	202.4	283.4	352.0	96.7	104.8	152.0	182.5	219.3	285.0	343.7
	11 days to ≤ 12 days	91.0	102.3	147.6	168.7	198.2	282.9	295.5	106.6	113.8	148.9	183.4	215.4	308.3	349.7
	12 days to ≤ 13 days	98.0	106.8	141.3	168.7	216.0	301.9	316.3	80.2	92.8	142.0	168.1	210.3	282.9	303.5
	13 days to ≤ 14 days	104.8	113.7	158.2	179.9	212.7	285.3	316.0	82.4	97.6	139.4	181.4	217.6	315.1	352.7
TYR	2 days to ≤ 3 days	26.2	29.5	58.0	85.9	114.8	265.2	392.3	29.8	32.5	61.2	84.4	116.3	225.3	282.0
	3 days to ≤ 4 days	31.1	35.5	60.2	77.0	108.7	253.2	296.6	33.5	37.1	62.4	86.4	121.9	294.6	416.3
	4 days to ≤ 5 days	28.6	34.6	61.8	87.5	125.2	265.2	313.4	32.2	39.7	65.7	93.7	119.1	242.4	411.1
	5 days to ≤ 6 days	33.6	37.8	63.8	88.6	119.1	325.3	455.0	34.3	39.5	63.3	85.3	120.1	287.5	503.5
	6 days to ≤ 7 days	36.7	38.2	61.5	84.7	113.1	209.8	267.3	34.8	39.7	61.0	84.3	112.9	191.9	258.6
	7 days to ≤ 8 days	42.0	48.5	66.3	88.9	112.2	239.4	252.9	37.6	42.3	63.0	80.3	108.4	193.8	249.5
	8 days to ≤ 9 days	39.1	40.4	59.0	78.8	101.4	171.5	198.7	41.1	42.9	64.9	84.9	98.2	159.9	307.4
	9 days to ≤ 10 days	33.0	36.6	65.3	88.8	118.8	155.9	165.9	39.6	46.8	68.3	89.4	110.8	179.2	227.6
	10 days to ≤ 11 days	34.5	36.5	61.2	87.7	119.5	224.4	249.3	34.1	43.0	67.1	91.8	115.7	167.1	205.7
	11 days to ≤ 12 days	44.0	47.0	68.3	90.7	118.1	215.1	386.1	42.7	46.4	74.3	97.4	123.6	202.6	287.1
	12 days to ≤ 13 days	46.8	53.7	68.4	88.3	111.2	181.3	288.7	22.4	32.6	69.1	88.9	120.1	184.7	220.2
	13 days to ≤ 14 days	47.5	51.1	75.9	91.2	108.0	157.9	172.2	43.8	49.6	74.2	96.1	124.8	282.9	337.4
VAL	2 days to ≤ 3 days	52.2	61.9	104.5	139.3	172.6	248.2	296.9	55.6	65.1	106.7	133.9	173.0	244.5	302.7
	3 days to ≤ 4 days	48.7	56.3	110.7	146.1	185.0	243.2	273.5	62.7	70.2	114.6	141.7	185.2	260.8	290.8
	4 days to ≤ 5 days	48.9	59.5	105.8	133.8	177.2	247.9	280.3	65.3	68.5	115.3	150.0	172.1	258.5	279.0
	5 days to ≤ 6 days	68.9	79.8	118.5	153.5	183.4	272.5	300.8	57.6	69.7	110.8	145.8	186.4	251.1	283.5
	6 days to ≤ 7 days	73.9	82.5	122.3	145.0	168.2	208.9	214.9	85.7	91.7	128.1	146.4	172.0	248.5	266.0
	7 days to ≤ 8 days	59.2	72.7	114.3	135.8	168.2	238.4	257.9	45.8	67.0	115.3	141.4	169.6	241.9	252.4
	8 days to ≤ 9 days	59.1	75.7	105.8	136.1	173.4	235.2	266.4	62.8	71.1	112.9	138.0	170.8	241.5	259.9
	9 days to ≤ 10 days	64.2	68.7	109.0	146.1	178.8	262.9	293.1	76.3	83.6	115.7	146.3	190.8	264.3	295.0
	10 days to ≤ 11 days	70.7	83.0	109.5	130.3	167.8	249.0	326.6	75.2	87.0	115.0	153.6	182.1	252.4	286.2
	11 days to ≤ 12 days	64.8	78.3	105.5	138.5	161.1	202.4	248.1	63.3	68.0	113.0	141.2	170.5	225.4	236.8

	12 days to ≤ 13 days	59.7	74.2	104.3	130.1	167.5	230.5	255.0	61.8	71.3	104.9	128.9	167.5	245.7	272.9
	13 days to ≤ 14 days	62.6	71.9	116.5	139.2	172.5	231.0	244.6	33.4	67.8	106.2	128.0	158.1	219.2	226.2
		Acylcarnitnes													
C0	2 days to ≤ 3 days	10.95	11.62	22.79	34.39	44.60	75.21	103.34	14.79	16.30	24.80	31.47	38.16	59.32	66.94
	3 days to ≤ 4 days	14.39	16.89	24.87	33.64	45.78	82.59	113.03	12.30	13.50	23.08	27.70	38.08	63.36	79.07
	4 days to ≤ 5 days	12.81	14.33	22.21	27.45	41.58	60.46	75.91	11.93	12.83	20.38	26.09	34.88	66.12	78.45
	5 days to ≤ 6 days	13.30	14.33	22.11	28.87	39.15	75.24	77.87	11.49	13.29	20.87	27.48	35.11	55.43	64.25
	6 days to ≤ 7 days	14.08	14.92	22.26	29.75	41.04	61.62	73.78	11.70	12.94	18.88	23.42	34.05	51.07	61.21
	7 days to ≤ 8 days	11.03	12.81	20.12	25.28	36.38	67.24	74.47	13.40	13.95	19.92	27.82	34.04	57.10	73.57
	8 days to ≤ 9 days	9.99	11.43	20.57	27.36	37.42	56.64	63.86	11.68	12.29	19.13	25.37	31.88	44.74	51.64
	9 days to ≤ 10 days	12.79	13.36	22.88	30.11	38.79	53.65	60.95	10.04	13.68	18.20	25.60	31.40	51.01	54.25
	10 days to ≤ 11 days	10.75	11.59	18.13	26.44	34.93	54.26	74.99	9.70	11.44	18.52	25.68	35.04	52.31	60.42
	11 days to ≤ 12 days	13.75	14.60	20.67	24.92	35.69	60.26	72.92	12.45	12.58	20.56	26.38	33.99	51.01	62.77
	12 days to ≤ 13 days	10.46	13.29	21.92	28.80	38.64	57.33	80.87	8.48	9.21	17.48	26.38	33.37	45.16	52.21
	13 days to ≤ 14 days	9.45	12.24	20.47	29.51	36.15	50.43	62.07	12.12	13.28	20.03	28.16	36.97	53.95	69.42
C2	2 days to ≤ 3 days	8.75	10.10	18.00	23.33	33.87	72.58	85.64	9.33	10.74	17.22	22.16	30.15	50.90	58.78
	3 days to ≤ 4 days	7.98	8.75	16.10	22.21	30.71	56.15	73.50	7.50	9.18	15.22	20.22	25.58	41.97	46.61
	4 days to ≤ 5 days	8.35	8.71	14.34	19.95	25.95	53.09	67.87	5.89	6.60	12.00	15.81	21.86	40.01	44.08
	5 days to ≤ 6 days	6.59	7.12	12.85	16.86	21.91	42.19	49.28	5.83	6.97	10.73	14.45	18.61	33.49	40.48
	6 days to ≤ 7 days	5.57	6.07	10.43	15.01	21.05	35.93	45.08	5.46	5.80	9.02	12.22	15.58	26.91	32.63
	7 days to ≤ 8 days	4.29	5.24	9.56	12.02	16.34	31.07	32.53	5.70	6.09	8.25	11.91	16.96	25.99	35.99
	8 days to ≤ 9 days	3.97	4.49	8.87	12.67	17.39	27.76	34.71	5.24	5.58	8.60	10.82	14.11	19.18	22.90
	9 days to ≤ 10 days	3.28	4.97	9.10	12.32	17.83	27.57	29.95	3.97	4.70	8.18	11.10	14.73	25.55	31.63
	10 days to ≤ 11 days	4.61	5.25	8.73	11.93	16.86	25.77	29.93	4.08	4.98	7.95	10.27	13.91	20.17	22.82
	11 days to ≤ 12 days	4.60	5.03	8.74	12.30	15.71	28.15	32.65	4.15	4.41	8.63	11.47	15.32	23.92	27.28
	12 days to ≤ 13 days	4.81	5.63	9.45	13.11	17.50	28.48	32.92	4.10	5.14	7.92	10.48	13.82	21.84	27.52
	13 days to ≤ 14 days	5.00	5.48	9.93	12.67	16.63	25.82	29.60	5.41	5.60	8.57	11.29	15.38	23.33	27.20
C3	2 days to ≤ 3 days	0.75	0.88	1.65	2.38	3.55	6.11	6.53	0.52	0.64	1.67	2.45	3.31	5.28	5.86
	3 days to ≤ 4 days	0.78	0.85	1.68	2.27	3.33	5.11	6.40	0.82	0.91	1.50	2.09	2.81	4.81	5.44
	4 days to ≤ 5 days	0.67	0.75	1.43	2.03	2.80	3.89	5.51	0.69	0.78	1.19	1.59	2.22	4.02	6.17
	5 days to ≤ 6 days	0.66	0.67	1.16	1.53	2.25	3.06	6.12	0.54	0.58	0.99	1.37	1.90	3.28	3.64
	6 days to ≤ 7 days	0.56	0.63	0.95	1.22	1.65	2.92	3.72	0.53	0.60	0.86	1.11	1.44	2.44	2.83
	7 days to ≤ 8 days	0.55	0.59	0.84	1.04	1.37	3.05	3.35	0.39	0.45	0.73	0.98	1.30	2.19	2.74
	8 days to ≤ 9 days	0.55	0.57	0.79	0.93	1.19	2.11	2.53	0.40	0.44	0.66	0.84	1.07	1.59	2.16
	9 days to ≤ 10 days	0.38	0.43	0.64	0.89	1.22	2.13	2.23	0.41	0.44	0.63	0.80	1.05	1.94	2.33
	10 days to ≤ 11 days	0.39	0.48	0.67	0.86	1.15	2.31	2.45	0.39	0.45	0.61	0.78	1.00	1.74	1.95
	11 days to ≤ 12 days	0.39	0.45	0.66	0.85	1.08	1.87	2.22	0.34	0.36	0.63	0.77	1.08	2.01	2.21
	12 days to ≤ 13 days	0.40	0.44	0.61	0.84	1.25	2.81	3.68	0.32	0.34	0.56	0.71	0.97	1.80	2.03
	13 days to ≤ 14 days	0.37	0.45	0.65	0.86	1.23	2.12	2.68	0.30	0.33	0.55	0.73	0.99	1.36	1.88
C3-DC+ C4-OH	2 days to ≤ 3 days	0.05	0.05	0.09	0.12	0.16	0.25	0.27	0.04	0.05	0.08	0.11	0.14	0.22	0.27
	3 days to ≤ 4 days	0.04	0.05	0.08	0.12	0.16	0.28	0.36	0.05	0.05	0.08	0.11	0.15	0.26	0.36
	4 days to ≤ 5 days	0.05	0.05	0.09	0.12	0.17	0.28	0.34	0.05	0.05	0.08	0.10	0.13	0.20	0.22
	5 days to ≤ 6 days	0.05	0.05	0.09	0.11	0.14	0.27	0.35	0.04	0.04	0.07	0.10	0.13	0.24	0.29
	6 days to ≤ 7 days	0.05	0.05	0.07	0.09	0.11	0.19	0.29	0.04	0.04	0.06	0.08	0.11	0.24	0.40
	7 days to ≤ 8 days	0.04	0.05	0.07	0.09	0.11	0.17	0.19	0.05	0.05	0.08	0.09	0.12	0.19	0.23
	8 days to ≤ 9 days	0.04	0.04	0.07	0.09	0.11	0.16	0.19	0.04	0.04	0.06	0.07	0.09	0.13	0.14

	9 days to ≤ 10 days	0.05	0.06	0.07	0.09	0.11	0.17	0.18	0.04	0.04	0.06	0.08	0.10	0.19	0.27
	10 days to ≤ 11 days	0.04	0.05	0.06	0.08	0.11	0.25	0.37	0.03	0.03	0.06	0.08	0.10	0.17	0.18
	11 days to ≤ 12 days	0.04	0.04	0.06	0.08	0.10	0.19	0.24	0.04	0.04	0.06	0.08	0.10	0.22	0.28
	12 days to ≤ 13 days	0.04	0.04	0.06	0.08	0.11	0.20	0.29	0.03	0.03	0.05	0.07	0.09	0.13	0.27
	13 days to ≤ 14 days	0.05	0.05	0.07	0.09	0.11	0.15	0.19	0.04	0.04	0.06	0.08	0.11	0.22	0.29
C4	2 days to ≤ 3 days	0.15	0.17	0.23	0.30	0.36	0.65	0.70	0.14	0.16	0.25	0.31	0.37	0.55	0.61
	3 days to ≤ 4 days	0.16	0.17	0.25	0.31	0.40	0.60	0.73	0.14	0.17	0.24	0.30	0.37	0.52	0.64
	4 days to ≤ 5 days	0.10	0.10	0.22	0.30	0.39	0.68	0.74	0.15	0.16	0.21	0.27	0.35	0.53	0.71
	5 days to ≤ 6 days	0.14	0.14	0.23	0.29	0.37	0.63	0.79	0.13	0.16	0.21	0.27	0.32	0.58	0.61
	6 days to ≤ 7 days	0.13	0.15	0.20	0.24	0.29	0.43	0.54	0.12	0.13	0.18	0.25	0.29	0.46	0.53
	7 days to ≤ 8 days	0.12	0.13	0.19	0.24	0.29	0.50	0.59	0.12	0.13	0.19	0.25	0.31	0.63	0.71
	8 days to ≤ 9 days	0.14	0.14	0.20	0.23	0.28	0.48	0.51	0.12	0.13	0.18	0.22	0.26	0.36	0.47
	9 days to ≤ 10 days	0.13	0.14	0.20	0.23	0.28	0.47	0.53	0.13	0.13	0.19	0.23	0.29	0.44	0.52
	10 days to ≤ 11 days	0.11	0.13	0.18	0.21	0.27	0.50	0.54	0.11	0.13	0.18	0.22	0.27	0.46	0.48
	11 days to ≤ 12 days	0.11	0.12	0.18	0.22	0.28	0.45	0.57	0.12	0.13	0.17	0.22	0.27	0.49	0.58
	12 days to ≤ 13 days	0.11	0.12	0.17	0.21	0.26	0.42	0.56	0.12	0.12	0.18	0.22	0.25	0.32	0.33
	13 days to ≤ 14 days	0.11	0.13	0.17	0.21	0.26	0.40	0.52	0.10	0.11	0.18	0.22	0.27	0.34	0.39
C4-DC+ C5-OH	2 days to ≤ 3 days	0.10	0.11	0.15	0.19	0.23	0.36	0.38	0.09	0.11	0.14	0.18	0.22	0.32	0.38
	3 days to ≤ 4 days	0.09	0.11	0.15	0.20	0.23	0.34	0.40	0.10	0.11	0.14	0.18	0.21	0.33	0.37
	4 days to ≤ 5 days	0.11	0.11	0.16	0.19	0.25	0.42	0.44	0.10	0.10	0.14	0.17	0.21	0.37	0.42
	5 days to ≤ 6 days	0.12	0.12	0.17	0.19	0.24	0.36	0.44	0.09	0.10	0.14	0.16	0.21	0.33	0.38
	6 days to ≤ 7 days	0.10	0.10	0.15	0.17	0.21	0.35	0.37	0.09	0.10	0.13	0.16	0.19	0.28	0.37
	7 days to ≤ 8 days	0.10	0.10	0.16	0.19	0.22	0.33	0.35	0.09	0.09	0.13	0.16	0.20	0.31	0.43
	8 days to ≤ 9 days	0.10	0.11	0.15	0.19	0.23	0.38	0.43	0.08	0.09	0.14	0.16	0.19	0.24	0.26
	9 days to ≤ 10 days	0.08	0.10	0.15	0.18	0.24	0.35	0.39	0.10	0.11	0.14	0.18	0.21	0.30	0.33
	10 days to ≤ 11 days	0.11	0.12	0.16	0.20	0.23	0.36	0.42	0.08	0.10	0.14	0.17	0.20	0.27	0.31
	11 days to ≤ 12 days	0.12	0.12	0.16	0.20	0.23	0.36	0.38	0.10	0.10	0.13	0.17	0.21	0.33	0.35
	12 days to ≤ 13 days	0.12	0.12	0.16	0.19	0.24	0.43	0.54	0.10	0.11	0.14	0.17	0.20	0.31	0.37
	13 days to ≤ 14 days	0.11	0.12	0.16	0.19	0.25	0.42	0.45	0.07	0.09	0.14	0.17	0.21	0.28	0.30
C5	2 days to ≤ 3 days	0.08	0.09	0.17	0.22	0.29	0.49	0.58	0.08	0.09	0.17	0.23	0.29	0.42	0.49
	3 days to ≤ 4 days	0.09	0.11	0.19	0.25	0.30	0.52	0.60	0.08	0.09	0.18	0.23	0.30	0.47	0.53
	4 days to ≤ 5 days	0.05	0.09	0.16	0.25	0.31	0.52	0.56	0.11	0.12	0.19	0.23	0.30	0.56	0.68
	5 days to ≤ 6 days	0.01	0.06	0.18	0.24	0.31	0.49	0.51	0.08	0.09	0.18	0.25	0.35	0.52	0.61
	6 days to ≤ 7 days	0.08	0.10	0.18	0.24	0.30	0.47	0.52	0.10	0.11	0.17	0.25	0.31	0.49	0.53
	7 days to ≤ 8 days	0.09	0.09	0.17	0.22	0.32	0.51	0.67	0.08	0.10	0.18	0.23	0.30	0.47	0.54
	8 days to ≤ 9 days	0.10	0.11	0.16	0.20	0.28	0.46	0.51	0.05	0.09	0.15	0.21	0.29	0.42	0.45
	9 days to ≤ 10 days	0.09	0.09	0.16	0.22	0.29	0.49	0.51	0.09	0.11	0.18	0.24	0.31	0.48	0.51
	10 days to ≤ 11 days	0.06	0.09	0.15	0.21	0.27	0.47	0.51	0.07	0.08	0.17	0.21	0.28	0.42	0.44
	11 days to ≤ 12 days	0.11	0.11	0.17	0.22	0.28	0.41	0.42	0.09	0.12	0.17	0.22	0.28	0.46	0.48
	12 days to ≤ 13 days	0.09	0.11	0.16	0.22	0.29	0.47	0.52	0.10	0.10	0.16	0.20	0.26	0.40	0.42
	13 days to ≤ 14 days	0.06	0.09	0.16	0.21	0.25	0.36	0.40	0.08	0.09	0.17	0.21	0.25	0.33	0.37
C5-DC+ C6-OH	2 days to ≤ 3 days	0.05	0.06	0.10	0.13	0.18	0.25	0.33	0.05	0.06	0.09	0.12	0.16	0.22	0.25
	3 days to ≤ 4 days	0.05	0.06	0.09	0.12	0.16	0.27	0.34	0.05	0.05	0.09	0.12	0.16	0.23	0.25
	4 days to ≤ 5 days	0.05	0.06	0.10	0.13	0.17	0.25	0.27	0.05	0.06	0.09	0.12	0.15	0.20	0.22
	5 days to ≤ 6 days	0.06	0.06	0.09	0.13	0.15	0.21	0.22	0.05	0.06	0.08	0.11	0.14	0.21	0.25

	6 days to ≤ 7 days	0.05	0.05	0.08	0.10	0.14	0.20	0.26	0.05	0.05	0.07	0.09	0.12	0.21	0.22
	7 days to ≤ 8 days	0.05	0.05	0.08	0.10	0.14	0.21	0.25	0.05	0.05	0.08	0.10	0.12	0.17	0.19
	8 days to ≤ 9 days	0.05	0.06	0.09	0.10	0.12	0.19	0.20	0.05	0.05	0.07	0.08	0.12	0.18	0.20
	9 days to ≤ 10 days	0.05	0.05	0.08	0.10	0.12	0.20	0.21	0.05	0.05	0.07	0.09	0.12	0.20	0.21
	10 days to ≤ 11 days	0.04	0.04	0.07	0.10	0.12	0.24	0.29	0.05	0.05	0.07	0.09	0.12	0.18	0.19
	11 days to ≤ 12 days	0.05	0.05	0.07	0.10	0.13	0.20	0.20	0.05	0.05	0.07	0.09	0.12	0.22	0.27
	12 days to ≤ 13 days	0.05	0.05	0.08	0.10	0.12	0.19	0.24	0.05	0.05	0.07	0.09	0.11	0.16	0.19
	13 days to ≤ 14 days	0.05	0.05	0.07	0.10	0.12	0.19	0.19	0.05	0.05	0.07	0.10	0.12	0.18	0.23
C6	2 days to ≤ 3 days	0.02	0.02	0.04	0.05	0.07	0.12	0.15	0.01	0.01	0.03	0.04	0.06	0.11	0.13
	3 days to ≤ 4 days	0.02	0.02	0.03	0.04	0.07	0.13	0.20	0.02	0.02	0.03	0.04	0.07	0.13	0.17
	4 days to ≤ 5 days	0.02	0.02	0.03	0.05	0.07	0.13	0.14	0.01	0.01	0.03	0.05	0.06	0.09	0.12
	5 days to ≤ 6 days	0.02	0.02	0.03	0.05	0.08	0.15	0.17	0.02	0.02	0.03	0.04	0.06	0.12	0.16
	6 days to ≤ 7 days	0.02	0.02	0.03	0.04	0.06	0.14	0.19	0.02	0.02	0.03	0.03	0.05	0.09	0.12
	7 days to ≤ 8 days	0.01	0.01	0.02	0.04	0.05	0.15	0.18	0.02	0.02	0.03	0.04	0.06	0.09	0.11
	8 days to ≤ 9 days	0.02	0.02	0.03	0.04	0.06	0.12	0.19	0.01	0.01	0.02	0.03	0.05	0.08	0.10
	9 days to ≤ 10 days	0.02	0.02	0.03	0.04	0.06	0.10	0.14	0.02	0.02	0.02	0.04	0.05	0.09	0.11
	10 days to ≤ 11 days	0.02	0.02	0.03	0.04	0.06	0.11	0.17	0.01	0.02	0.02	0.03	0.05	0.13	0.16
	11 days to ≤ 12 days	0.02	0.02	0.03	0.04	0.06	0.11	0.15	0.02	0.02	0.03	0.04	0.06	0.15	0.15
	12 days to ≤ 13 days	0.02	0.02	0.03	0.04	0.06	0.11	0.12	0.02	0.02	0.03	0.04	0.06	0.10	0.12
	13 days to ≤ 14 days	0.02	0.02	0.03	0.04	0.06	0.10	0.12	0.01	0.02	0.03	0.05	0.06	0.10	0.12
C6-DC	2 days to ≤ 3 days	0.03	0.03	0.05	0.08	0.13	0.22	0.24	0.03	0.03	0.05	0.08	0.12	0.20	0.22
	3 days to ≤ 4 days	0.03	0.03	0.05	0.08	0.12	0.22	0.24	0.02	0.03	0.05	0.08	0.13	0.21	0.27
	4 days to ≤ 5 days	0.03	0.04	0.06	0.10	0.14	0.22	0.29	0.03	0.03	0.06	0.09	0.12	0.17	0.19
	5 days to ≤ 6 days	0.03	0.04	0.06	0.08	0.13	0.19	0.21	0.03	0.03	0.06	0.09	0.13	0.20	0.25
	6 days to ≤ 7 days	0.03	0.04	0.05	0.08	0.11	0.19	0.34	0.03	0.04	0.05	0.07	0.11	0.19	0.21
	7 days to ≤ 8 days	0.03	0.04	0.05	0.08	0.10	0.19	0.37	0.03	0.04	0.05	0.07	0.10	0.18	0.19
	8 days to ≤ 9 days	0.03	0.04	0.05	0.07	0.10	0.19	0.28	0.04	0.04	0.06	0.08	0.11	0.19	0.22
	9 days to ≤ 10 days	0.04	0.04	0.05	0.07	0.09	0.20	0.24	0.04	0.04	0.06	0.07	0.11	0.19	0.31
	10 days to ≤ 11 days	0.03	0.04	0.05	0.07	0.10	0.20	0.25	0.04	0.04	0.06	0.08	0.11	0.18	0.20
	11 days to ≤ 12 days	0.04	0.04	0.06	0.08	0.11	0.20	0.25	0.04	0.04	0.06	0.08	0.11	0.27	0.36
	12 days to ≤ 13 days	0.04	0.04	0.06	0.07	0.10	0.20	0.24	0.03	0.03	0.05	0.07	0.11	0.19	0.24
	13 days to ≤ 14 days	0.03	0.04	0.05	0.07	0.12	0.22	0.29	0.04	0.04	0.05	0.08	0.12	0.20	0.29
C8	2 days to ≤ 3 days	0.02	0.03	0.04	0.06	0.08	0.14	0.19	0.02	0.02	0.04	0.05	0.08	0.13	0.19
	3 days to ≤ 4 days	0.03	0.03	0.05	0.06	0.08	0.13	0.17	0.02	0.03	0.04	0.06	0.08	0.12	0.14
	4 days to ≤ 5 days	0.03	0.03	0.05	0.07	0.09	0.14	0.15	0.03	0.03	0.05	0.07	0.08	0.12	0.14
	5 days to ≤ 6 days	0.03	0.03	0.05	0.07	0.09	0.16	0.19	0.03	0.03	0.05	0.06	0.09	0.17	0.18
	6 days to ≤ 7 days	0.03	0.03	0.05	0.07	0.09	0.17	0.22	0.02	0.02	0.04	0.06	0.09	0.16	0.17
	7 days to ≤ 8 days	0.03	0.03	0.05	0.06	0.09	0.15	0.17	0.04	0.04	0.05	0.06	0.08	0.14	0.16
	8 days to ≤ 9 days	0.03	0.03	0.05	0.07	0.10	0.18	0.21	0.02	0.03	0.04	0.06	0.07	0.13	0.16
	9 days to ≤ 10 days	0.04	0.04	0.06	0.07	0.09	0.17	0.19	0.03	0.03	0.05	0.07	0.10	0.19	0.24
	10 days to ≤ 11 days	0.04	0.04	0.06	0.07	0.10	0.16	0.21	0.03	0.03	0.05	0.07	0.09	0.16	0.20
	11 days to ≤ 12 days	0.04	0.04	0.06	0.08	0.10	0.16	0.20	0.03	0.04	0.06	0.08	0.10	0.22	0.26
	12 days to ≤ 13 days	0.04	0.04	0.06	0.07	0.09	0.14	0.14	0.03	0.03	0.05	0.07	0.10	0.19	0.22
	13 days to ≤ 14 days	0.04	0.04	0.06	0.07	0.10	0.18	0.26	0.02	0.03	0.05	0.07	0.11	0.16	0.20
C8:1	2 days to ≤ 3 days	0.04	0.04	0.09	0.13	0.18	0.36	0.43	0.03	0.03	0.09	0.13	0.18	0.33	0.36
	3 days to ≤ 4 days	0.05	0.06	0.11	0.16	0.20	0.38	0.45	0.03	0.04	0.10	0.15	0.20	0.34	0.42
	4 days to ≤ 5 days	0.06	0.06	0.11	0.15	0.20	0.35	0.49	0.05	0.06	0.11	0.15	0.21	0.53	0.59

	5 days to ≤ 6 days	0.06	0.06	0.10	0.15	0.20	0.43	0.53	0.05	0.06	0.12	0.17	0.23	0.39	0.49
	6 days to ≤ 7 days	0.06	0.07	0.11	0.15	0.22	0.57	0.65	0.05	0.06	0.11	0.16	0.23	0.50	0.65
	7 days to ≤ 8 days	0.04	0.05	0.09	0.13	0.22	0.50	0.68	0.05	0.06	0.10	0.13	0.21	0.43	0.57
	8 days to ≤ 9 days	0.05	0.06	0.10	0.16	0.22	0.48	0.61	0.04	0.05	0.09	0.14	0.20	0.38	0.55
	9 days to ≤ 10 days	0.05	0.06	0.11	0.14	0.19	0.39	0.49	0.05	0.05	0.10	0.15	0.24	0.45	0.52
	10 days to ≤ 11 days	0.04	0.04	0.10	0.14	0.22	0.54	0.66	0.05	0.06	0.10	0.15	0.23	0.52	0.58
	11 days to ≤ 12 days	0.05	0.06	0.10	0.15	0.21	0.35	0.54	0.06	0.06	0.10	0.15	0.22	0.43	0.62
	12 days to ≤ 13 days	0.06	0.06	0.11	0.15	0.22	0.47	0.53	0.06	0.06	0.10	0.14	0.18	0.33	0.38
	13 days to ≤ 14 days	0.06	0.06	0.10	0.14	0.20	0.46	0.52	0.05	0.05	0.10	0.15	0.23	0.42	0.57
C10	2 days to ≤ 3 days	0.02	0.02	0.03	0.05	0.07	0.15	0.20	0.01	0.01	0.03	0.05	0.07	0.12	0.14
	3 days to ≤ 4 days	0.03	0.03	0.04	0.06	0.08	0.14	0.21	0.02	0.02	0.04	0.05	0.08	0.14	0.21
	4 days to ≤ 5 days	0.02	0.02	0.04	0.06	0.08	0.11	0.15	0.02	0.03	0.04	0.06	0.08	0.13	0.16
	5 days to ≤ 6 days	0.02	0.02	0.04	0.06	0.08	0.14	0.19	0.02	0.02	0.04	0.06	0.08	0.13	0.18
	6 days to ≤ 7 days	0.02	0.02	0.04	0.05	0.08	0.14	0.19	0.02	0.02	0.03	0.05	0.08	0.12	0.14
	7 days to ≤ 8 days	0.02	0.02	0.03	0.05	0.07	0.15	0.20	0.03	0.03	0.04	0.06	0.07	0.14	0.15
	8 days to ≤ 9 days	0.02	0.02	0.04	0.05	0.08	0.15	0.17	0.02	0.02	0.03	0.05	0.06	0.11	0.14
	9 days to ≤ 10 days	0.02	0.02	0.04	0.05	0.08	0.12	0.16	0.02	0.03	0.04	0.06	0.08	0.17	0.22
	10 days to ≤ 11 days	0.02	0.02	0.04	0.05	0.08	0.15	0.23	0.02	0.02	0.04	0.05	0.08	0.13	0.15
	11 days to ≤ 12 days	0.03	0.03	0.04	0.06	0.07	0.10	0.16	0.03	0.03	0.04	0.06	0.08	0.19	0.21
	12 days to ≤ 13 days	0.02	0.02	0.04	0.06	0.07	0.12	0.14	0.02	0.02	0.04	0.06	0.08	0.16	0.21
	13 days to ≤ 14 days	0.02	0.03	0.04	0.06	0.08	0.14	0.18	0.02	0.03	0.04	0.06	0.08	0.12	0.15
C10:1	2 days to ≤ 3 days	0.02	0.03	0.04	0.06	0.08	0.16	0.20	0.02	0.02	0.04	0.06	0.08	0.13	0.18
	3 days to ≤ 4 days	0.03	0.03	0.05	0.07	0.09	0.16	0.20	0.03	0.03	0.05	0.07	0.08	0.13	0.15
	4 days to ≤ 5 days	0.02	0.03	0.05	0.07	0.09	0.13	0.15	0.02	0.02	0.05	0.06	0.09	0.12	0.13
	5 days to ≤ 6 days	0.03	0.03	0.05	0.07	0.08	0.15	0.16	0.03	0.03	0.05	0.07	0.08	0.12	0.14
	6 days to ≤ 7 days	0.03	0.03	0.05	0.06	0.08	0.17	0.18	0.02	0.03	0.04	0.06	0.09	0.12	0.13
	7 days to ≤ 8 days	0.02	0.02	0.04	0.06	0.08	0.13	0.16	0.02	0.03	0.04	0.06	0.08	0.13	0.14
	8 days to ≤ 9 days	0.02	0.03	0.05	0.06	0.08	0.14	0.16	0.02	0.02	0.04	0.06	0.08	0.12	0.14
	9 days to ≤ 10 days	0.02	0.02	0.05	0.06	0.08	0.13	0.19	0.03	0.03	0.04	0.06	0.09	0.14	0.20
	10 days to ≤ 11 days	0.02	0.03	0.04	0.05	0.08	0.16	0.23	0.02	0.02	0.04	0.06	0.08	0.15	0.17
	11 days to ≤ 12 days	0.02	0.03	0.04	0.06	0.08	0.12	0.16	0.03	0.03	0.05	0.06	0.08	0.14	0.15
	12 days to ≤ 13 days	0.02	0.03	0.04	0.06	0.08	0.15	0.20	0.02	0.02	0.04	0.05	0.08	0.13	0.15
	13 days to ≤ 14 days	0.02	0.02	0.04	0.06	0.08	0.13	0.17	0.03	0.03	0.05	0.06	0.08	0.13	0.15
C12	2 days to ≤ 3 days	0.01	0.02	0.03	0.05	0.08	0.16	0.24	0.02	0.02	0.03	0.04	0.06	0.12	0.18
	3 days to ≤ 4 days	0.02	0.02	0.03	0.05	0.07	0.14	0.16	0.02	0.02	0.03	0.04	0.06	0.14	0.30
	4 days to ≤ 5 days	0.02	0.02	0.03	0.05	0.06	0.11	0.14	0.02	0.02	0.03	0.04	0.06	0.09	0.11
	5 days to ≤ 6 days	0.02	0.02	0.03	0.04	0.06	0.13	0.16	0.02	0.02	0.03	0.04	0.05	0.08	0.09
	6 days to ≤ 7 days	0.02	0.02	0.03	0.04	0.05	0.13	0.14	0.01	0.01	0.02	0.03	0.05	0.10	0.12
	7 days to ≤ 8 days	0.02	0.02	0.03	0.04	0.06	0.10	0.15	0.02	0.02	0.03	0.04	0.06	0.10	0.11
	8 days to ≤ 9 days	0.02	0.02	0.03	0.04	0.05	0.08	0.10	0.02	0.02	0.03	0.04	0.05	0.07	0.08
	9 days to ≤ 10 days	0.02	0.02	0.03	0.04	0.06	0.11	0.18	0.02	0.02	0.03	0.04	0.05	0.09	0.11
	10 days to ≤ 11 days	0.02	0.02	0.03	0.04	0.05	0.07	0.09	0.01	0.01	0.02	0.04	0.05	0.08	0.09
	11 days to ≤ 12 days	0.01	0.02	0.03	0.04	0.06	0.09	0.11	0.02	0.02	0.03	0.04	0.06	0.08	0.09
	12 days to ≤ 13 days	0.02	0.02	0.03	0.05	0.06	0.09	0.10	0.01	0.01	0.03	0.04	0.06	0.08	0.14
	13 days to ≤ 14 days	0.01	0.01	0.03	0.04	0.06	0.09	0.12	0.02	0.02	0.03	0.04	0.06	0.09	0.09
C12:1	2 days to ≤ 3 days	0.02	0.02	0.03	0.04	0.06	0.12	0.16	0.01	0.01	0.02	0.04	0.05	0.13	0.17
	3 days to ≤ 4 days	0.01	0.01	0.02	0.04	0.06	0.12	0.17	0.01	0.01	0.02	0.04	0.06	0.12	0.21

	4 days to ≤ 5 days	0.01	0.01	0.02	0.04	0.06	0.12	0.18	0.01	0.01	0.02	0.04	0.06	0.11	0.14
	5 days to ≤ 6 days	0.01	0.01	0.02	0.03	0.05	0.12	0.16	0.01	0.01	0.02	0.03	0.05	0.10	0.16
	6 days to ≤ 7 days	0.01	0.01	0.02	0.03	0.04	0.13	0.17	0.01	0.01	0.02	0.03	0.04	0.10	0.12
	7 days to ≤ 8 days	0.01	0.01	0.02	0.03	0.05	0.11	0.15	0.01	0.01	0.02	0.03	0.04	0.09	0.12
	8 days to ≤ 9 days	0.01	0.01	0.02	0.03	0.05	0.09	0.10	0.01	0.01	0.02	0.02	0.04	0.08	0.09
	9 days to ≤ 10 days	0.01	0.01	0.02	0.03	0.04	0.08	0.08	0.01	0.01	0.02	0.03	0.04	0.09	0.16
	10 days to ≤ 11 days	0.02	0.02	0.02	0.02	0.03	0.06	0.06	0.01	0.01	0.02	0.03	0.04	0.08	0.10
	11 days to ≤ 12 days	0.01	0.01	0.02	0.03	0.04	0.08	0.11	0.02	0.02	0.02	0.03	0.03	0.05	0.05
	12 days to ≤ 13 days	0.01	0.01	0.02	0.03	0.04	0.08	0.09	0.01	0.01	0.02	0.02	0.04	0.07	0.09
	13 days to ≤ 14 days	0.01	0.01	0.02	0.03	0.04	0.07	0.12	0.01	0.01	0.02	0.03	0.04	0.08	0.09
C14	2 days to ≤ 3 days	0.06	0.07	0.12	0.16	0.23	0.44	0.54	0.04	0.06	0.11	0.15	0.20	0.36	0.49
	3 days to ≤ 4 days	0.04	0.05	0.10	0.14	0.20	0.38	0.45	0.05	0.06	0.10	0.13	0.18	0.33	0.37
	4 days to ≤ 5 days	0.05	0.05	0.10	0.13	0.19	0.40	0.49	0.05	0.05	0.08	0.10	0.14	0.22	0.38
	5 days to ≤ 6 days	0.05	0.05	0.09	0.12	0.16	0.32	0.48	0.04	0.04	0.08	0.10	0.14	0.26	0.32
	6 days to ≤ 7 days	0.04	0.05	0.09	0.12	0.16	0.31	0.45	0.04	0.04	0.06	0.09	0.13	0.22	0.26
	7 days to ≤ 8 days	0.03	0.04	0.08	0.11	0.17	0.31	0.41	0.04	0.04	0.07	0.09	0.14	0.23	0.24
	8 days to ≤ 9 days	0.04	0.05	0.08	0.11	0.14	0.25	0.30	0.04	0.04	0.07	0.09	0.12	0.23	0.32
	9 days to ≤ 10 days	0.03	0.03	0.08	0.11	0.16	0.26	0.38	0.05	0.05	0.07	0.09	0.13	0.27	0.32
	10 days to ≤ 11 days	0.03	0.04	0.07	0.10	0.13	0.21	0.22	0.04	0.04	0.06	0.09	0.11	0.17	0.19
	11 days to ≤ 12 days	0.04	0.04	0.08	0.11	0.15	0.27	0.37	0.04	0.04	0.07	0.09	0.12	0.21	0.25
	12 days to ≤ 13 days	0.05	0.05	0.09	0.11	0.15	0.28	0.37	0.03	0.03	0.06	0.09	0.13	0.24	0.31
	13 days to ≤ 14 days	0.03	0.04	0.07	0.10	0.14	0.22	0.28	0.05	0.06	0.08	0.10	0.13	0.21	0.22
C14:1	2 days to ≤ 3 days	0.02	0.03	0.05	0.07	0.10	0.16	0.20	0.04	0.04	0.06	0.07	0.09	0.14	0.14
	3 days to ≤ 4 days	0.03	0.03	0.05	0.07	0.09	0.16	0.19	0.03	0.03	0.05	0.06	0.08	0.12	0.15
	4 days to ≤ 5 days	0.03	0.03	0.05	0.07	0.08	0.13	0.17	0.01	0.02	0.04	0.06	0.07	0.11	0.12
	5 days to ≤ 6 days	0.03	0.03	0.04	0.05	0.07	0.14	0.15	0.02	0.03	0.04	0.05	0.07	0.09	0.10
	6 days to ≤ 7 days	0.03	0.03	0.04	0.04	0.06	0.13	0.16	0.02	0.02	0.03	0.04	0.06	0.11	0.14
	7 days to ≤ 8 days	0.03	0.03	0.04	0.05	0.06	0.10	0.11	0.02	0.02	0.03	0.04	0.06	0.09	0.12
	8 days to ≤ 9 days	0.02	0.02	0.03	0.04	0.06	0.11	0.12	0.02	0.02	0.03	0.04	0.05	0.08	0.09
	9 days to ≤ 10 days	0.02	0.02	0.03	0.04	0.05	0.10	0.14	0.02	0.02	0.03	0.04	0.06	0.12	0.16
	10 days to ≤ 11 days	0.02	0.02	0.03	0.04	0.05	0.09	0.10	0.02	0.02	0.03	0.04	0.05	0.08	0.09
	11 days to ≤ 12 days	0.02	0.02	0.03	0.04	0.05	0.08	0.11	0.02	0.02	0.03	0.04	0.05	0.07	0.08
	12 days to ≤ 13 days	0.02	0.02	0.03	0.04	0.05	0.08	0.09	0.03	0.03	0.03	0.04	0.04	0.06	0.06
	13 days to ≤ 14 days	0.02	0.02	0.03	0.04	0.05	0.08	0.11	0.02	0.02	0.03	0.04	0.05	0.07	0.08
C16	2 days to ≤ 3 days	0.51	0.71	1.35	2.02	2.95	5.27	5.64	0.68	0.80	1.33	1.83	2.52	4.51	5.51
	3 days to ≤ 4 days	0.54	0.74	1.27	1.74	2.62	4.80	5.72	0.69	0.77	1.28	1.60	2.21	3.92	4.83
	4 days to ≤ 5 days	0.59	0.66	1.17	1.65	2.52	4.62	5.45	0.67	0.77	1.07	1.30	1.65	2.80	3.70
	5 days to ≤ 6 days	0.60	0.67	1.06	1.41	2.14	3.77	5.62	0.51	0.59	0.91	1.26	1.79	3.35	3.44
	6 days to ≤ 7 days	0.61	0.64	1.11	1.40	2.04	4.09	4.56	0.48	0.55	0.84	1.17	1.57	2.84	3.14
	7 days to ≤ 8 days	0.56	0.66	0.95	1.35	1.80	3.84	4.70	0.52	0.59	0.86	1.07	1.58	2.65	3.09
	8 days to ≤ 9 days	0.51	0.61	0.95	1.24	1.57	2.69	3.39	0.48	0.51	0.80	1.04	1.35	2.47	3.32
	9 days to ≤ 10 days	0.42	0.47	0.86	1.14	1.53	2.59	3.32	0.49	0.59	0.78	1.02	1.28	2.89	3.52
	10 days to ≤ 11 days	0.45	0.47	0.83	1.06	1.37	1.86	1.97	0.44	0.46	0.72	0.92	1.35	2.16	2.36
	11 days to ≤ 12 days	0.51	0.53	0.80	0.99	1.24	2.17	2.55	0.35	0.40	0.68	0.89	1.17	1.84	2.45
	12 days to ≤ 13 days	0.40	0.45	0.79	1.02	1.40	2.56	3.25	0.44	0.46	0.66	0.86	1.10	1.97	2.47
	13 days to ≤ 14 days	0.45	0.49	0.73	0.96	1.29	2.17	3.36	0.42	0.46	0.65	0.84	1.10	2.15	2.19
C16:1	2 days to ≤ 3 days	0.04	0.05	0.10	0.14	0.23	0.48	0.52	0.03	0.04	0.08	0.12	0.19	0.38	0.53

	3 days to ≤ 4 days	0.03	0.04	0.08	0.12	0.17	0.42	0.53	0.04	0.05	0.08	0.11	0.16	0.34	0.41
	4 days to ≤ 5 days	0.04	0.04	0.08	0.11	0.17	0.38	0.50	0.03	0.04	0.06	0.08	0.12	0.22	0.29
	5 days to ≤ 6 days	0.03	0.04	0.07	0.10	0.14	0.33	0.47	0.03	0.03	0.05	0.07	0.10	0.25	0.30
	6 days to ≤ 7 days	0.04	0.04	0.06	0.08	0.12	0.38	0.40	0.02	0.02	0.04	0.06	0.09	0.18	0.21
	7 days to ≤ 8 days	0.02	0.03	0.05	0.07	0.11	0.22	0.31	0.02	0.03	0.04	0.06	0.10	0.21	0.27
	8 days to ≤ 9 days	0.03	0.03	0.05	0.06	0.08	0.13	0.17	0.02	0.03	0.04	0.06	0.07	0.14	0.19
	9 days to ≤ 10 days	0.02	0.02	0.04	0.06	0.09	0.21	0.31	0.02	0.03	0.04	0.05	0.07	0.12	0.14
	10 days to ≤ 11 days	0.02	0.02	0.04	0.06	0.08	0.15	0.19	0.03	0.03	0.04	0.05	0.06	0.12	0.14
	11 days to ≤ 12 days	0.03	0.03	0.04	0.05	0.07	0.16	0.20	0.03	0.03	0.04	0.05	0.06	0.12	0.14
	12 days to ≤ 13 days	0.03	0.03	0.05	0.06	0.08	0.14	0.15	0.02	0.02	0.04	0.05	0.07	0.12	0.20
	13 days to ≤ 14 days	0.03	0.03	0.04	0.06	0.08	0.19	0.23	0.02	0.03	0.04	0.05	0.07	0.13	0.14
C16:1-OH	2 days to ≤ 3 days	0.03	0.03	0.03	0.04	0.05	0.06	0.06	0.01	0.01	0.02	0.03	0.04	0.06	0.07
	3 days to ≤ 4 days	0.02	0.02	0.03	0.03	0.04	0.06	0.06	0.02	0.02	0.03	0.03	0.04	0.05	0.06
	4 days to ≤ 5 days	0.01	0.01	0.02	0.03	0.04	0.06	0.07	0.02	0.02	0.02	0.03	0.03	0.05	0.06
	5 days to ≤ 6 days	0.01	0.02	0.02	0.03	0.04	0.05	0.06	0.02	0.02	0.02	0.03	0.03	0.05	0.05
	6 days to ≤ 7 days	0.02	0.02	0.02	0.03	0.03	0.05	0.05	0.02	0.02	0.02	0.03	0.03	0.05	0.06
	7 days to ≤ 8 days	0.01	0.01	0.02	0.03	0.04	0.06	0.07	0.01	0.01	0.02	0.02	0.03	0.04	0.04
	8 days to ≤ 9 days	0.01	0.01	0.02	0.02	0.03	0.05	0.05	0.01	0.01	0.02	0.02	0.03	0.04	0.04
	9 days to ≤ 10 days	0.01	0.02	0.02	0.02	0.03	0.05	0.05	0.02	0.02	0.02	0.02	0.03	0.04	0.05
	10 days to ≤ 11 days	0.02	0.02	0.02	0.02	0.03	0.05	0.05	0.02	0.02	0.02	0.02	0.02	0.03	0.03
	11 days to ≤ 12 days	0.02	0.02	0.02	0.02	0.03	0.04	0.05	0.02	0.02	0.02	0.02	0.02	0.03	0.03
	12 days to ≤ 13 days	0.02	0.02	0.02	0.02	0.03	0.04	0.05	0.01	0.01	0.01	0.02	0.02	0.03	0.03
	13 days to ≤ 14 days	0.01	0.01	0.01	0.02	0.02	0.04	0.06	0.02	0.02	0.02	0.02	0.02	0.03	0.03
C18	2 days to ≤ 3 days	0.27	0.31	0.59	0.80	1.05	1.63	1.83	0.31	0.35	0.64	0.78	0.99	1.41	1.53
	3 days to ≤ 4 days	0.30	0.40	0.61	0.80	1.03	1.56	1.85	0.33	0.42	0.57	0.72	0.92	1.43	1.67
	4 days to ≤ 5 days	0.30	0.32	0.59	0.71	0.97	1.42	1.69	0.36	0.39	0.57	0.67	0.79	1.08	1.17
	5 days to ≤ 6 days	0.37	0.37	0.53	0.66	0.87	1.53	1.69	0.26	0.32	0.51	0.66	0.84	1.27	1.36
	6 days to ≤ 7 days	0.35	0.36	0.59	0.74	0.92	1.24	1.63	0.32	0.33	0.53	0.63	0.79	1.33	1.46
	7 days to ≤ 8 days	0.32	0.39	0.56	0.68	0.92	1.42	1.64	0.32	0.33	0.49	0.63	0.75	1.12	1.23
	8 days to ≤ 9 days	0.30	0.33	0.53	0.70	0.87	1.32	1.46	0.33	0.35	0.49	0.60	0.72	1.08	1.30
	9 days to ≤ 10 days	0.28	0.30	0.49	0.61	0.76	1.33	1.44	0.27	0.31	0.48	0.59	0.75	1.22	1.31
	10 days to ≤ 11 days	0.27	0.31	0.47	0.60	0.74	1.03	1.09	0.23	0.27	0.44	0.61	0.78	0.96	1.10
	11 days to ≤ 12 days	0.26	0.32	0.44	0.56	0.69	1.13	1.34	0.26	0.28	0.41	0.51	0.65	0.95	1.02
	12 days to ≤ 13 days	0.24	0.29	0.41	0.57	0.70	1.11	1.21	0.25	0.26	0.41	0.46	0.61	0.90	0.99
	13 days to ≤ 14 days	0.22	0.25	0.39	0.52	0.65	0.93	1.00	0.23	0.28	0.40	0.49	0.63	0.93	1.13
C18:1	2 days to ≤ 3 days	0.42	0.61	0.93	1.17	1.52	2.29	2.62	0.41	0.50	0.92	1.11	1.40	2.03	2.11
	3 days to ≤ 4 days	0.44	0.57	0.91	1.21	1.56	2.37	2.51	0.53	0.60	0.89	1.09	1.39	2.15	2.35
	4 days to ≤ 5 days	0.59	0.60	0.92	1.15	1.58	2.59	3.27	0.39	0.43	0.83	1.00	1.20	1.80	1.97
	5 days to ≤ 6 days	0.57	0.60	0.90	1.07	1.40	2.11	2.17	0.45	0.58	0.79	1.03	1.33	2.06	2.36
	6 days to ≤ 7 days	0.61	0.63	0.94	1.19	1.61	2.48	2.61	0.44	0.55	0.75	0.99	1.33	1.99	2.15
	7 days to ≤ 8 days	0.49	0.58	0.87	1.22	1.61	2.75	3.43	0.44	0.52	0.79	1.06	1.33	2.16	2.40
	8 days to ≤ 9 days	0.44	0.51	0.87	1.07	1.45	2.02	2.44	0.46	0.51	0.73	0.89	1.12	1.65	2.38
	9 days to ≤ 10 days	0.43	0.47	0.81	1.08	1.45	2.58	2.95	0.46	0.49	0.75	0.91	1.21	2.04	2.67
	10 days to ≤ 11 days	0.42	0.45	0.75	0.97	1.26	1.92	2.04	0.48	0.49	0.68	0.90	1.14	1.90	2.38
	11 days to ≤ 12 days	0.50	0.53	0.74	0.91	1.17	2.02	2.37	0.35	0.42	0.66	0.88	1.08	1.60	2.02
	12 days to ≤ 13 days	0.54	0.56	0.78	1.05	1.22	2.19	2.40	0.39	0.41	0.61	0.78	1.04	1.84	1.92
	13 days to ≤ 14 days	0.40	0.44	0.68	0.82	1.04	1.64	1.80	0.40	0.41	0.61	0.77	1.00	1.59	1.68

C18:2	2 days to ≤ 3 days	0.06	0.09	0.25	0.43	0.64	1.16	1.34	0.09	0.13	0.28	0.43	0.62	0.99	1.11
	3 days to ≤ 4 days	0.11	0.14	0.35	0.55	0.78	1.29	1.46	0.10	0.14	0.29	0.44	0.62	1.08	1.28
	4 days to ≤ 5 days	0.20	0.22	0.44	0.56	0.75	1.19	1.40	0.13	0.16	0.39	0.51	0.67	0.94	1.17
	5 days to ≤ 6 days	0.11	0.13	0.41	0.58	0.75	1.09	1.43	0.08	0.13	0.36	0.52	0.71	1.18	1.32
	6 days to ≤ 7 days	0.08	0.11	0.41	0.61	0.80	1.15	1.23	0.18	0.22	0.41	0.50	0.66	1.00	1.04
	7 days to ≤ 8 days	0.17	0.19	0.48	0.60	0.80	1.25	1.47	0.16	0.21	0.38	0.53	0.68	0.95	1.14
	8 days to ≤ 9 days	0.21	0.24	0.45	0.59	0.79	1.23	1.36	0.20	0.23	0.35	0.49	0.63	0.88	1.00
	9 days to ≤ 10 days	0.23	0.26	0.40	0.54	0.71	1.21	1.45	0.16	0.18	0.35	0.46	0.59	0.81	0.98
	10 days to ≤ 11 days	0.14	0.18	0.38	0.52	0.67	0.99	1.09	0.22	0.24	0.36	0.46	0.60	0.77	0.91
	11 days to ≤ 12 days	0.19	0.23	0.38	0.48	0.62	0.91	1.08	0.16	0.19	0.32	0.43	0.54	0.81	0.92
	12 days to ≤ 13 days	0.20	0.21	0.35	0.45	0.61	1.02	1.31	0.13	0.15	0.29	0.36	0.47	0.75	0.85
	13 days to ≤ 14 days	0.12	0.16	0.31	0.43	0.58	0.91	0.98	0.16	0.17	0.30	0.37	0.47	0.75	0.86

Abbreviation: ALA, alanine; ARG, arginine; CIT, citrulline; GLY, glycine; LEU, leucine; ILE, isoleucine; ALLO-ILE, alloisoleucine; PRO-OH, hydroxyproline; MET, methionine; ORN, ornithine; PHE, phenylalanine; PRO, proline; TYR, Tyrosine; VAL, valine; C0, free carnitine; C2, acetylcarnitine; C3, propionylcarnitine; C3-DC+C4-OH, malonylcarnitine+3-hydroxybutyrylcarnitine; C4, butyrylcarnitine+isobutyrylcarnitine; C4-DC+C5-OH, methylmalonylcarnitine+3-hydroxyisovalerylcarnitine; C5, isovalerylcarnitine+methylbutyrylcarnitine; C5-DC+C6-OH, glutarylcarnitine+3-hydroxyhexanoylcarnitine; C6, hexanoylcarnitine; C6-DC, methylglutarylcarnitine; C8, octanoylcarnitine; C8:1, octenoylcarnitine; C10, decanoylcarnitine; C10:1, decenoylcarnitine; C12, dodecanoylcarnitine; C12:1, dodecenoylcarnitine; C14, tetradecanoylcarnitine; C14:1, tetradecenoylcarnitine; C16, palmitoylcarnitine; C16:1, palmitoleylcarnitine; C16:1-OH, 3-hydroxypalmitoleylcarnitine; C18, stearoylcarnitine; C18:1, oleoylcarnitine; C18:2, linoleoylcarnitine.

Table S5. The 0.5^{th}, 2.5^{th}, 25th, 50th, 75th ,$97.5^{th}$and 99.5^{th} percentiles calculated by age and sex for 35 MS/MS NBS biomarkers for the preterm neonates of 1500g-2499g (μM)

		Amino acids													
		Male							Female						
**Analytes**	**Age**	**0.5^{th}**	**2.5^{th}**	**25th**	**50th**	**75th**	**97.5^{th}**	**99.5^{th}**	**0.5^{th}**	**2.5^{th}**	**25th**	**50th**	**75th**	**97.5^{th}**	**99.5^{th}**
ALA	2 days to ≤ 3 days	124.9	141.5	215.3	270.3	338.5	516.7	650.2	113.6	141.5	223.4	275.3	339.8	483.9	526.0
	3 days to ≤ 4 days	126.8	147.7	224.1	280.3	350.7	535.6	639.4	144.1	158.8	234.5	293.5	361.3	543.5	666.3
	4 days to ≤ 5 days	122.5	147.3	225.9	278.7	342.7	514.2	570.9	120.2	148.1	236.3	291.2	354.7	496.0	542.8
	5 days to ≤ 6 days	131.1	154.9	229.5	280.9	342.4	494.1	565.2	147.3	172.2	243.9	293.4	356.6	520.8	591.3
	6 days to ≤ 7 days	125.4	155.0	228.1	277.9	339.1	477.4	517.5	144.6	166.0	241.3	293.0	352.6	514.5	573.9
	7 days to ≤ 8 days	136.9	159.9	227.0	275.6	332.5	472.2	534.2	143.5	170.5	247.4	295.4	358.2	515.2	570.4
	8 days to ≤ 9 days	144.6	162.0	228.8	281.5	344.4	509.5	621.4	157.5	175.7	242.5	295.4	359.7	540.8	630.3
	9 days to ≤ 10 days	121.1	154.0	229.9	279.7	337.8	454.5	492.6	142.5	167.7	242.8	289.0	358.3	501.9	577.3
	10 days to ≤ 11 days	125.0	150.1	226.7	276.0	338.1	480.2	538.6	144.9	169.8	244.6	290.4	353.0	507.8	559.5
	11 days to ≤ 12 days	133.2	159.9	226.4	272.5	334.2	462.7	517.3	147.3	168.7	235.9	287.4	350.9	515.8	597.3
	12 days to ≤ 13 days	145.1	159.7	227.3	272.1	333.3	504.4	609.9	140.7	163.4	239.5	291.1	347.8	493.6	539.8
	13 days to ≤ 14 days	141.0	155.9	227.7	271.8	333.9	483.1	553.2	127.4	156.3	228.1	278.5	342.8	459.1	509.9
ARG	2 days to ≤ 3 days	1.5	2.1	5.7	10.3	17.4	43.7	59.4	1.4	1.9	5.2	9.4	16.2	40.7	57.3
	3 days to ≤ 4 days	1.7	2.3	6.5	11.9	20.3	47.7	62.8	1.6	2.1	6.1	11.0	19.2	45.8	62.3
	4 days to ≤ 5 days	2.0	2.8	7.9	13.7	24.1	53.8	68.5	1.8	2.3	7.4	13.3	23.1	50.3	64.2
	5 days to ≤ 6 days	2.0	2.8	8.6	14.6	24.8	55.1	69.2	2.1	2.6	8.2	14.6	24.2	54.9	67.1
	6 days to ≤ 7 days	2.0	2.6	8.5	15.3	26.4	55.8	68.6	2.0	2.6	8.1	14.3	24.2	54.0	66.2
	7 days to ≤ 8 days	2.0	2.7	8.1	15.3	24.7	54.7	68.5	2.3	3.0	9.0	14.9	24.3	52.4	67.3
	8 days to ≤ 9 days	2.3	3.0	8.9	15.9	25.1	56.1	68.6	2.2	2.8	8.5	15.1	24.4	52.9	68.9
	9 days to ≤ 10 days	2.3	3.0	8.6	15.6	25.1	52.5	65.2	1.8	2.4	7.6	14.2	23.3	51.3	64.3
	10 days to ≤ 11 days	2.2	3.2	9.1	15.8	25.3	52.9	68.4	2.1	2.8	8.7	15.4	24.1	51.8	66.6
	11 days to ≤ 12 days	2.6	3.3	9.3	15.6	24.7	50.6	68.3	2.2	2.9	8.8	15.7	25.1	49.5	61.8
	12 days to ≤ 13 days	2.0	2.8	9.1	15.7	25.4	50.5	69.0	2.3	2.9	8.6	15.7	25.0	49.9	65.9
	13 days to ≤ 14 days	2.5	3.5	9.8	16.8	26.9	51.2	65.1	2.1	2.7	9.2	15.8	24.8	48.3	63.2
CIT	2 days to ≤ 3 days	6.9	7.6	10.9	13.3	16.4	24.9	31.5	7.0	7.8	11.0	13.5	16.6	24.9	30.8
	3 days to ≤ 4 days	6.9	7.7	11.1	13.6	16.9	25.5	31.8	7.3	8.0	11.5	14.0	17.3	25.8	31.4
	4 days to ≤ 5 days	6.9	7.7	11.3	14.0	17.3	26.1	31.6	7.4	8.2	11.6	14.3	17.7	27.0	32.4
	5 days to ≤ 6 days	7.0	8.0	11.7	14.4	17.9	26.5	31.8	7.4	8.1	12.1	14.9	18.5	28.2	32.9
	6 days to ≤ 7 days	7.0	7.8	11.9	14.6	18.1	26.7	32.9	7.3	8.3	12.2	15.0	18.6	28.2	34.8
	7 days to ≤ 8 days	7.2	8.1	12.0	14.7	18.3	27.7	32.0	7.4	8.6	12.4	15.4	18.7	27.7	31.7
	8 days to ≤ 9 days	7.3	8.6	12.3	15.2	19.2	28.8	34.0	7.1	8.4	12.3	15.4	19.1	30.1	34.7
	9 days to ≤ 10 days	7.2	8.2	12.3	15.3	19.2	28.3	34.2	7.5	8.5	12.5	15.4	19.0	28.5	34.7
	10 days to ≤ 11 days	7.2	8.3	12.2	15.3	19.4	29.8	36.8	7.9	8.7	12.8	16.0	19.9	31.1	36.3
	11 days to ≤ 12 days	7.5	8.7	12.8	16.0	19.9	29.8	36.1	7.7	9.0	13.1	16.0	19.8	29.9	35.1
	12 days to ≤ 13 days	7.7	8.7	12.8	15.9	19.7	29.7	36.7	7.8	8.9	13.2	16.4	20.3	32.1	36.9
	13 days to ≤ 14 days	7.3	8.3	12.7	16.0	20.1	31.2	40.4	7.9	9.0	13.3	16.5	19.9	30.6	35.2
GLY	2 days to ≤ 3 days	250.4	281.2	396.4	483.6	595.9	918.4	1113.4	261.6	296.6	415.5	502.8	615.3	907.1	1069.5
	3 days to ≤ 4 days	235.7	270.4	378.9	464.9	576.0	886.7	1082.7	251.0	282.9	395.2	478.7	585.9	897.0	1075.0
	4 days to ≤ 5 days	222.7	255.3	347.1	419.5	520.7	810.2	968.0	232.8	263.1	365.1	442.5	546.1	840.8	1039.1

	5 days to ≤ 6 days	215.2	242.5	336.3	404.5	498.8	788.7	936.2	223.8	256.6	353.8	424.0	517.3	790.1	934.6
	6 days to ≤ 7 days	204.7	230.3	321.8	391.9	488.3	773.2	970.6	217.9	248.4	338.9	406.8	505.8	788.5	927.6
	7 days to ≤ 8 days	209.0	229.5	321.1	389.9	478.4	713.3	812.7	226.7	251.2	343.1	410.2	500.0	779.3	922.6
	8 days to ≤ 9 days	200.5	233.0	320.7	389.7	481.1	727.7	843.9	228.4	249.1	340.1	407.6	491.9	757.8	949.3
	9 days to ≤ 10 days	209.9	232.9	318.9	390.2	474.9	722.0	880.3	227.7	246.1	338.3	406.4	486.6	753.6	879.8
	10 days to ≤ 11 days	203.4	229.8	322.7	384.6	471.2	724.9	828.3	220.3	245.0	330.7	395.7	492.4	761.0	964.2
	11 days to ≤ 12 days	207.6	232.3	321.4	381.4	472.3	711.6	831.5	218.2	239.9	334.6	399.3	485.2	731.4	852.5
	12 days to ≤ 13 days	206.8	230.0	317.6	386.2	467.3	725.9	876.4	203.9	237.9	325.0	397.0	486.5	778.2	942.0
	13 days to ≤ 14 days	195.3	219.1	304.2	370.0	457.1	686.7	853.4	208.4	225.9	317.5	380.5	468.1	725.7	900.9
LEU/ILE/ALLO-ILE/PRO-OH	2 days to ≤ 3 days	69.9	78.7	115.0	142.2	175.3	265.1	310.9	69.7	79.5	116.4	143.5	177.7	261.5	308.4
	3 days to ≤ 4 days	73.9	83.5	124.0	153.8	189.8	282.9	324.3	77.7	87.9	127.3	155.7	191.2	286.1	333.2
	4 days to ≤ 5 days	74.2	84.4	129.0	162.0	202.0	292.3	341.3	78.6	88.7	134.0	164.7	206.9	298.9	338.4
	5 days to ≤ 6 days	83.5	95.6	137.4	169.6	210.2	298.8	345.9	82.0	97.6	140.3	172.4	215.7	302.8	354.8
	6 days to ≤ 7 days	85.5	96.1	139.9	170.6	209.0	302.6	342.7	84.2	97.2	142.8	173.6	214.1	308.0	343.4
	7 days to ≤ 8 days	91.2	99.9	139.5	168.1	203.7	292.7	336.6	90.8	103.3	145.2	176.2	213.6	312.4	361.9
	8 days to ≤ 9 days	89.1	100.4	139.9	169.0	204.4	298.1	334.0	88.3	100.7	142.1	171.7	210.1	299.0	338.8
	9 days to ≤ 10 days	85.1	98.4	138.5	165.6	199.9	281.6	326.0	88.6	100.6	143.4	171.1	205.5	279.5	317.3
	10 days to ≤ 11 days	85.4	97.8	136.4	162.9	193.4	281.0	318.5	90.7	99.9	138.7	166.6	199.1	288.4	346.7
	11 days to ≤ 12 days	87.5	98.6	135.0	160.4	190.3	270.5	307.6	89.7	101.4	138.5	164.5	196.6	279.0	316.6
	12 days to ≤ 13 days	88.4	96.3	132.8	159.8	189.4	261.1	300.0	91.0	99.6	138.8	165.3	196.8	283.1	334.5
	13 days to ≤ 14 days	86.2	97.0	132.2	158.5	189.8	263.0	314.8	89.4	98.7	134.3	159.7	187.7	261.4	294.0
MET	2 days to ≤ 3 days	8.0	9.3	14.7	19.0	24.8	40.9	51.1	8.7	10.0	15.3	19.4	25.0	41.2	50.6
	3 days to ≤ 4 days	8.1	9.5	15.2	19.7	25.8	42.5	50.8	8.4	9.9	15.4	20.0	25.9	42.4	52.4
	4 days to ≤ 5 days	8.5	10.0	15.8	20.8	27.6	45.6	52.8	8.8	10.2	16.2	21.1	27.8	44.2	52.4
	5 days to ≤ 6 days	8.9	10.2	16.3	21.2	28.0	45.6	54.4	9.1	10.5	16.9	21.6	28.5	46.5	55.6
	6 days to ≤ 7 days	8.8	10.1	16.7	21.4	28.2	46.5	58.1	9.1	10.8	16.9	21.6	28.5	45.3	54.9
	7 days to ≤ 8 days	8.7	10.1	16.4	21.6	28.0	45.0	56.7	9.2	10.8	16.9	22.1	28.3	44.7	57.7
	8 days to ≤ 9 days	9.3	10.8	17.1	21.7	28.3	46.3	56.4	9.3	11.1	17.3	22.1	28.2	44.9	56.1
	9 days to ≤ 10 days	8.8	10.6	16.5	21.9	27.9	43.9	51.3	9.2	10.7	17.1	21.8	27.9	43.2	50.4
	10 days to ≤ 11 days	9.0	10.6	16.9	21.8	27.9	44.0	53.4	9.3	10.8	17.1	21.9	27.6	44.1	50.7
	11 days to ≤ 12 days	9.5	10.9	17.4	21.7	26.8	42.9	50.0	9.3	10.5	17.0	21.9	27.7	42.2	52.1
	12 days to ≤ 13 days	8.0	9.7	16.5	21.6	26.4	40.8	46.0	9.2	10.8	17.2	22.0	28.0	45.5	53.4
	13 days to ≤ 14 days	8.7	10.1	16.1	21.3	26.9	42.1	51.7	9.4	10.6	16.7	21.1	27.2	41.5	50.9
ORN	2 days to ≤ 3 days	39.8	46.5	77.6	103.9	142.2	268.2	358.9	41.1	48.3	78.1	103.7	140.2	252.7	331.8
	3 days to ≤ 4 days	40.6	48.8	84.5	114.3	154.9	279.0	358.1	43.8	51.9	87.3	116.0	154.3	277.2	352.5
	4 days to ≤ 5 days	43.1	52.1	89.5	121.7	162.9	280.2	358.6	46.2	56.4	92.5	123.8	166.5	280.1	379.0
	5 days to ≤ 6 days	49.1	58.7	95.8	124.5	166.5	287.9	365.4	49.7	58.9	95.2	126.7	165.3	278.7	350.2
	6 days to ≤ 7 days	47.3	56.5	92.6	125.1	164.0	288.1	366.0	48.6	57.6	94.6	123.0	160.4	279.6	342.8
	7 days to ≤ 8 days	49.0	58.5	92.8	120.9	162.4	277.8	379.8	52.4	61.9	96.3	123.9	166.8	299.3	388.4
	8 days to ≤ 9 days	47.8	59.5	94.5	123.6	161.9	279.9	346.1	53.1	62.3	97.3	123.4	159.7	268.8	331.4
	9 days to ≤ 10 days	53.0	60.6	94.8	121.3	162.4	283.9	373.6	54.4	61.4	99.5	127.4	162.8	285.8	358.9
	10 days to ≤ 11 days	55.6	63.9	95.1	119.7	156.5	267.3	336.0	56.9	64.3	96.0	121.0	153.3	269.7	339.6
	11 days to ≤ 12 days	54.7	62.4	94.5	119.3	154.8	259.3	312.8	56.5	63.1	97.7	121.7	154.7	274.6	330.3
	12 days to ≤ 13 days	57.1	66.1	96.3	122.6	154.9	278.3	342.1	56.4	63.7	97.1	122.7	158.0	285.2	348.2

	13 days to ≤ 14 days	56.5	64.3	93.2	119.0	152.7	267.1	332.7	55.0	60.2	96.3	120.8	151.8	259.3	306.9
PHE	2 days to ≤ 3 days	33.5	37.7	50.1	59.2	70.7	100.9	115.7	33.6	37.0	49.6	58.3	69.2	98.7	113.5
	3 days to ≤ 4 days	33.2	37.1	50.6	60.2	73.2	106.3	120.0	32.7	36.6	50.1	59.5	72.0	104.7	117.3
	4 days to ≤ 5 days	31.8	36.3	50.6	61.7	75.4	105.9	118.7	31.3	36.0	50.0	60.8	73.9	106.3	117.0
	5 days to ≤ 6 days	31.1	35.0	51.1	61.2	74.2	102.3	113.4	33.0	36.4	50.6	60.5	73.2	100.4	114.5
	6 days to ≤ 7 days	31.4	36.0	49.6	59.4	71.4	98.9	109.3	31.6	35.4	49.0	58.9	71.1	97.8	109.3
	7 days to ≤ 8 days	32.0	35.7	48.0	56.6	68.1	96.2	112.7	31.4	35.2	47.8	57.1	69.0	97.1	111.8
	8 days to ≤ 9 days	30.4	33.3	46.6	55.6	66.1	94.1	109.3	30.5	34.5	46.2	55.2	66.4	93.0	110.5
	9 days to ≤ 10 days	29.1	32.2	44.9	53.1	63.3	91.2	105.3	31.0	34.4	46.4	54.6	64.4	88.3	99.2
	10 days to ≤ 11 days	28.5	31.6	44.2	52.2	61.7	86.8	98.9	30.2	32.7	44.3	52.6	62.2	93.4	108.2
	11 days to ≤ 12 days	30.0	32.3	43.6	51.7	61.3	87.1	99.5	29.6	32.1	43.7	51.5	61.5	86.5	96.8
	12 days to ≤ 13 days	28.4	32.0	42.9	50.8	60.2	87.9	101.6	29.6	32.7	43.9	51.4	60.9	86.2	100.3
	13 days to ≤ 14 days	29.4	31.9	42.7	50.0	58.7	82.5	98.2	28.5	30.2	42.4	49.1	58.3	80.7	94.8
PRO	2 days to ≤ 3 days	80.5	91.8	136.6	167.6	204.2	293.8	343.9	85.6	96.4	141.9	173.2	209.7	304.9	359.4
	3 days to ≤ 4 days	80.9	91.2	140.2	172.9	210.5	305.5	358.0	87.0	99.0	146.9	178.8	215.3	308.7	358.0
	4 days to ≤ 5 days	81.0	92.2	140.4	172.9	206.8	294.0	342.6	86.5	102.4	147.3	178.3	212.5	294.2	338.9
	5 days to ≤ 6 days	94.4	103.5	148.3	177.0	210.2	294.5	355.6	95.9	107.4	152.6	181.6	214.0	290.3	331.1
	6 days to ≤ 7 days	94.3	106.9	150.2	177.4	211.6	297.2	345.4	103.5	112.9	154.8	183.0	216.5	291.2	349.5
	7 days to ≤ 8 days	96.9	107.4	147.6	179.1	213.4	311.8	367.8	105.4	117.7	156.5	185.9	219.3	308.3	360.7
	8 days to ≤ 9 days	98.1	107.0	149.9	180.2	215.9	303.1	348.5	98.9	109.8	153.0	184.9	219.2	309.0	361.6
	9 days to ≤ 10 days	97.0	107.8	150.6	179.7	211.9	293.6	340.5	104.9	117.7	153.9	183.0	214.6	290.4	336.4
	10 days to ≤ 11 days	98.6	109.1	148.0	175.1	206.7	300.5	342.5	102.5	112.7	152.2	181.8	212.5	302.8	338.1
	11 days to ≤ 12 days	99.0	106.7	146.9	175.2	206.2	286.7	321.8	102.6	112.6	153.7	179.2	211.5	290.7	325.0
	12 days to ≤ 13 days	98.0	107.7	146.8	172.1	204.1	293.3	342.0	96.5	108.5	148.9	177.0	210.5	297.6	348.6
	13 days to ≤ 14 days	97.8	109.1	146.7	172.7	207.4	292.5	336.5	98.0	107.0	145.8	173.7	206.1	296.3	343.8
TYR	2 days to ≤ 3 days	40.5	47.6	78.9	105.5	143.8	295.8	399.3	41.8	49.0	80.7	107.9	146.0	291.3	394.1
	3 days to ≤ 4 days	42.0	49.0	83.2	109.6	147.2	283.3	373.3	44.7	53.0	88.6	118.5	159.0	303.3	414.8
	4 days to ≤ 5 days	40.9	49.3	82.8	110.8	148.7	293.6	405.4	44.1	51.7	87.5	114.0	148.7	274.7	361.6
	5 days to ≤ 6 days	45.8	52.4	85.2	112.1	147.7	301.9	422.9	46.5	54.1	89.5	119.0	156.5	291.8	388.5
	6 days to ≤ 7 days	44.3	51.2	83.4	108.8	142.5	264.9	360.5	44.9	51.4	85.8	112.4	149.1	263.2	355.9
	7 days to ≤ 8 days	44.6	50.7	82.6	107.1	140.9	248.4	368.0	45.6	52.4	87.4	114.8	149.6	243.9	335.9
	8 days to ≤ 9 days	44.3	49.4	80.7	105.4	138.4	261.9	334.2	46.0	52.1	85.2	110.6	144.1	247.0	336.0
	9 days to ≤ 10 days	44.9	49.8	80.5	103.8	134.2	222.6	303.5	45.9	52.7	84.2	110.3	143.6	253.3	306.2
	10 days to ≤ 11 days	43.1	49.4	78.6	102.6	130.7	228.0	298.7	46.4	53.7	84.7	106.4	137.1	222.3	287.7
	11 days to ≤ 12 days	43.8	51.1	77.7	100.3	129.0	226.7	318.0	46.9	52.7	83.6	107.6	136.1	216.4	279.2
	12 days to ≤ 13 days	43.4	48.6	77.0	97.8	128.7	215.0	280.1	48.7	53.4	82.7	107.1	135.9	224.1	287.6
	13 days to ≤ 14 days	42.9	50.7	75.4	95.6	123.5	210.7	278.2	43.9	49.9	79.3	100.7	128.5	218.3	239.7
VAL	2 days to ≤ 3 days	51.5	62.9	97.9	122.0	152.5	224.4	254.1	50.6	63.8	99.2	123.5	153.7	222.2	251.3
	3 days to ≤ 4 days	53.0	66.8	106.0	132.5	164.3	235.2	259.5	53.2	70.2	109.5	135.9	166.4	235.5	258.0
	4 days to ≤ 5 days	56.2	68.5	109.7	140.6	173.8	249.9	283.1	60.2	74.9	114.8	143.5	177.4	250.9	287.3
	5 days to ≤ 6 days	65.8	78.1	116.5	143.9	175.7	250.9	287.4	67.3	79.5	120.1	147.6	181.6	254.5	290.0
	6 days to ≤ 7 days	58.2	74.7	113.7	142.7	174.2	245.9	274.7	66.1	76.7	119.3	146.5	181.8	252.7	274.6
	7 days to ≤ 8 days	63.2	76.2	112.5	136.3	166.3	231.0	258.3	64.3	78.3	118.4	146.2	179.2	256.6	276.9
	8 days to ≤ 9 days	61.0	76.2	111.6	138.0	168.4	227.4	253.0	63.8	79.8	114.1	139.3	175.2	247.5	280.2
	9 days to ≤ 10 days	57.8	73.2	108.9	131.1	160.1	223.8	241.6	64.9	76.8	115.2	139.5	167.5	231.5	254.6
	10 days to ≤ 11 days	57.2	68.3	106.8	129.8	157.3	214.7	237.1	66.9	77.8	111.6	133.5	162.4	224.6	246.2
	11 days to ≤ 12 days	56.2	71.7	105.9	126.8	152.2	216.5	230.3	64.3	75.6	108.7	131.0	158.4	214.7	236.2

	12 days to ≤ 13 days	58.3	71.2	103.3	124.6	150.8	209.8	233.0	63.6	74.7	108.7	131.8	159.0	226.4	244.5
	13 days to ≤ 14 days	61.4	73.7	100.4	121.6	150.8	213.9	230.2	61.9	75.2	106.3	126.7	152.9	208.7	226.8
								Acylcarnitnes							
C0	2 days to ≤ 3 days	12.38	14.33	21.93	27.91	35.38	56.00	68.44	11.79	13.39	20.00	24.99	31.37	48.58	59.18
	3 days to ≤ 4 days	12.70	14.84	22.36	28.60	36.08	57.94	70.50	12.04	13.67	20.61	25.72	32.06	48.93	61.11
	4 days to ≤ 5 days	13.17	14.87	22.52	28.23	36.10	57.52	69.59	12.36	14.07	20.74	25.82	32.30	49.78	62.57
	5 days to ≤ 6 days	12.57	14.66	22.38	28.38	35.68	55.56	66.94	11.98	14.08	21.02	26.40	32.91	50.26	60.93
	6 days to ≤ 7 days	12.32	14.54	22.53	28.69	35.88	54.81	67.51	12.26	13.85	21.04	26.32	32.81	48.59	59.25
	7 days to ≤ 8 days	12.51	13.65	21.75	27.60	35.27	54.74	69.92	11.57	13.30	20.73	26.79	33.72	51.57	61.29
	8 days to ≤ 9 days	12.55	14.54	22.29	28.48	35.53	54.59	63.95	11.65	13.14	20.44	26.37	32.82	49.39	58.90
	9 days to ≤ 10 days	11.45	13.39	21.41	27.70	35.31	56.27	68.53	11.50	13.15	20.34	25.65	32.32	47.51	59.52
	10 days to ≤ 11 days	11.32	13.10	20.79	27.00	33.94	51.97	61.08	11.06	12.66	20.22	25.91	33.38	50.07	55.95
	11 days to ≤ 12 days	12.26	13.71	21.56	27.45	35.04	56.59	67.50	10.85	12.25	19.91	25.89	31.87	45.95	55.91
	12 days to ≤ 13 days	11.45	12.93	20.74	26.72	33.82	55.85	71.61	11.01	12.61	19.98	25.21	32.05	48.56	58.45
	13 days to ≤ 14 days	11.47	12.44	20.63	26.53	34.00	51.39	57.96	10.79	12.00	20.00	25.15	32.06	47.11	57.16
C2	2 days to ≤ 3 days	9.03	10.52	17.09	21.89	27.93	44.10	53.94	8.14	9.68	15.56	19.74	24.98	38.56	45.52
	3 days to ≤ 4 days	7.74	9.33	15.67	20.24	25.94	41.39	50.00	7.54	8.91	14.41	18.20	23.00	36.09	42.60
	4 days to ≤ 5 days	7.07	8.27	13.54	17.23	21.94	35.99	41.99	6.58	7.75	12.37	15.76	19.90	31.07	38.01
	5 days to ≤ 6 days	6.22	7.18	11.77	15.07	19.23	30.01	36.92	6.37	7.24	11.29	14.04	17.77	28.74	34.11
	6 days to ≤ 7 days	5.48	6.56	10.50	13.57	17.36	27.93	34.04	5.51	6.24	10.06	12.67	16.12	26.27	33.20
	7 days to ≤ 8 days	5.18	5.93	9.57	12.28	15.78	25.88	32.55	4.81	5.68	9.12	11.74	14.98	23.38	26.91
	8 days to ≤ 9 days	4.73	5.58	9.20	11.83	15.49	24.60	31.28	4.73	5.43	8.63	11.21	14.29	22.76	28.29
	9 days to ≤ 10 days	4.63	5.54	8.80	11.46	14.72	23.78	29.76	4.70	5.32	8.34	10.62	13.56	21.31	26.55
	10 days to ≤ 11 days	4.66	5.17	8.93	11.36	14.50	23.10	28.94	4.32	5.00	8.41	10.88	13.77	21.80	27.53
	11 days to ≤ 12 days	4.79	5.55	8.93	11.58	15.12	25.05	32.80	4.27	4.89	8.47	10.84	13.84	21.72	28.31
	12 days to ≤ 13 days	4.46	5.24	8.79	11.42	14.64	24.27	30.25	4.21	4.89	8.13	10.73	13.71	22.45	26.88
	13 days to ≤ 14 days	4.56	5.28	8.79	11.38	14.53	24.29	30.51	4.24	5.05	8.06	10.53	13.34	22.86	29.04
C3	2 days to ≤ 3 days	0.76	0.93	1.59	2.14	2.90	4.70	5.81	0.76	0.90	1.50	1.99	2.63	4.35	5.31
	3 days to ≤ 4 days	0.70	0.85	1.51	2.03	2.74	4.63	5.59	0.70	0.83	1.40	1.87	2.50	4.17	5.05
	4 days to ≤ 5 days	0.69	0.80	1.33	1.77	2.37	4.16	5.22	0.63	0.75	1.23	1.63	2.18	3.63	4.58
	5 days to ≤ 6 days	0.55	0.65	1.11	1.46	1.99	3.38	4.27	0.56	0.65	1.04	1.37	1.84	3.14	4.05
	6 days to ≤ 7 days	0.51	0.60	0.92	1.24	1.61	2.83	3.75	0.49	0.58	0.89	1.17	1.55	2.59	3.33
	7 days to ≤ 8 days	0.41	0.48	0.79	1.03	1.39	2.30	3.10	0.42	0.48	0.78	1.01	1.34	2.25	2.90
	8 days to ≤ 9 days	0.40	0.46	0.74	0.97	1.31	2.23	2.88	0.37	0.42	0.70	0.94	1.24	2.17	2.95
	9 days to ≤ 10 days	0.35	0.41	0.68	0.90	1.21	2.15	2.75	0.36	0.41	0.65	0.86	1.14	2.11	2.74
	10 days to ≤ 11 days	0.36	0.41	0.66	0.85	1.14	2.11	2.82	0.37	0.40	0.63	0.83	1.10	1.90	2.47
	11 days to ≤ 12 days	0.38	0.42	0.66	0.86	1.16	2.11	3.21	0.34	0.42	0.61	0.82	1.09	1.92	2.54
	12 days to ≤ 13 days	0.33	0.39	0.62	0.81	1.10	2.25	3.01	0.34	0.39	0.62	0.81	1.09	2.00	2.68
	13 days to ≤ 14 days	0.31	0.36	0.60	0.80	1.09	2.14	2.74	0.29	0.34	0.56	0.74	1.02	1.80	2.62
C3-DC+ C4-OH	2 days to ≤ 3 days	0.04	0.05	0.08	0.11	0.16	0.32	0.42	0.04	0.05	0.08	0.11	0.16	0.31	0.41
	3 days to ≤ 4 days	0.04	0.04	0.07	0.10	0.14	0.30	0.40	0.04	0.04	0.07	0.10	0.14	0.30	0.41
	4 days to ≤ 5 days	0.04	0.04	0.07	0.09	0.12	0.24	0.32	0.03	0.04	0.06	0.09	0.12	0.26	0.32
	5 days to ≤ 6 days	0.04	0.04	0.06	0.08	0.11	0.23	0.30	0.04	0.04	0.06	0.08	0.11	0.25	0.32
	6 days to ≤ 7 days	0.04	0.04	0.06	0.08	0.10	0.21	0.26	0.04	0.04	0.06	0.08	0.10	0.21	0.27
	7 days to ≤ 8 days	0.04	0.04	0.06	0.08	0.10	0.20	0.25	0.04	0.04	0.06	0.08	0.10	0.22	0.28
	8 days to ≤ 9 days	0.04	0.04	0.06	0.08	0.10	0.20	0.26	0.04	0.04	0.06	0.08	0.10	0.22	0.29

	9 days to ≤ 10 days	0.04	0.04	0.06	0.07	0.10	0.20	0.24	0.04	0.04	0.06	0.07	0.10	0.21	0.26
	10 days to ≤ 11 days	0.04	0.04	0.06	0.07	0.09	0.16	0.19	0.03	0.03	0.05	0.07	0.09	0.21	0.27
	11 days to ≤ 12 days	0.04	0.04	0.06	0.07	0.09	0.18	0.21	0.04	0.04	0.06	0.07	0.09	0.18	0.21
	12 days to ≤ 13 days	0.04	0.04	0.06	0.07	0.10	0.19	0.23	0.04	0.04	0.06	0.07	0.10	0.22	0.27
	13 days to ≤ 14 days	0.03	0.03	0.05	0.07	0.09	0.22	0.29	0.03	0.03	0.05	0.07	0.09	0.21	0.28
C4	2 days to ≤ 3 days	0.11	0.13	0.19	0.24	0.29	0.43	0.51	0.11	0.13	0.20	0.25	0.30	0.44	0.51
	3 days to ≤ 4 days	0.11	0.13	0.19	0.23	0.28	0.41	0.46	0.12	0.14	0.20	0.24	0.29	0.42	0.47
	4 days to ≤ 5 days	0.09	0.11	0.18	0.22	0.27	0.40	0.48	0.09	0.12	0.19	0.22	0.27	0.40	0.45
	5 days to ≤ 6 days	0.10	0.12	0.18	0.21	0.26	0.39	0.46	0.10	0.12	0.18	0.22	0.26	0.37	0.40
	6 days to ≤ 7 days	0.10	0.12	0.17	0.21	0.24	0.34	0.39	0.10	0.12	0.17	0.21	0.25	0.35	0.42
	7 days to ≤ 8 days	0.08	0.10	0.16	0.20	0.24	0.34	0.39	0.09	0.11	0.17	0.21	0.25	0.34	0.38
	8 days to ≤ 9 days	0.09	0.11	0.16	0.20	0.23	0.33	0.38	0.09	0.11	0.17	0.20	0.24	0.34	0.39
	9 days to ≤ 10 days	0.08	0.10	0.16	0.19	0.23	0.35	0.40	0.09	0.11	0.16	0.19	0.23	0.32	0.36
	10 days to ≤ 11 days	0.07	0.09	0.15	0.18	0.23	0.32	0.36	0.08	0.10	0.16	0.19	0.23	0.33	0.39
	11 days to ≤ 12 days	0.08	0.10	0.15	0.19	0.23	0.33	0.38	0.10	0.11	0.16	0.19	0.23	0.32	0.36
	12 days to ≤ 13 days	0.09	0.10	0.15	0.18	0.22	0.32	0.36	0.07	0.08	0.15	0.19	0.23	0.33	0.36
	13 days to ≤ 14 days	0.08	0.10	0.15	0.18	0.22	0.33	0.38	0.09	0.10	0.15	0.18	0.22	0.31	0.35
C4-DC+ C5-OH	2 days to ≤ 3 days	0.10	0.11	0.15	0.18	0.22	0.32	0.39	0.09	0.10	0.14	0.17	0.20	0.29	0.35
	3 days to ≤ 4 days	0.10	0.11	0.15	0.18	0.22	0.32	0.39	0.09	0.10	0.14	0.17	0.20	0.29	0.35
	4 days to ≤ 5 days	0.10	0.11	0.15	0.18	0.21	0.31	0.36	0.08	0.09	0.14	0.16	0.20	0.29	0.37
	5 days to ≤ 6 days	0.09	0.10	0.14	0.17	0.21	0.31	0.39	0.08	0.10	0.14	0.16	0.20	0.30	0.36
	6 days to ≤ 7 days	0.09	0.10	0.14	0.17	0.21	0.32	0.40	0.08	0.10	0.13	0.16	0.20	0.29	0.40
	7 days to ≤ 8 days	0.09	0.10	0.14	0.17	0.21	0.31	0.38	0.08	0.09	0.13	0.16	0.20	0.31	0.37
	8 days to ≤ 9 days	0.09	0.10	0.14	0.17	0.21	0.31	0.37	0.09	0.10	0.13	0.16	0.19	0.28	0.34
	9 days to ≤ 10 days	0.09	0.10	0.14	0.17	0.21	0.30	0.36	0.09	0.10	0.13	0.16	0.19	0.29	0.35
	10 days to ≤ 11 days	0.10	0.11	0.14	0.17	0.20	0.31	0.37	0.09	0.09	0.13	0.16	0.19	0.27	0.31
	11 days to ≤ 12 days	0.10	0.10	0.14	0.17	0.20	0.31	0.35	0.09	0.10	0.13	0.16	0.19	0.28	0.33
	12 days to ≤ 13 days	0.10	0.11	0.14	0.17	0.21	0.31	0.38	0.09	0.10	0.13	0.16	0.19	0.28	0.36
	13 days to ≤ 14 days	0.09	0.10	0.14	0.17	0.21	0.32	0.41	0.09	0.09	0.13	0.16	0.19	0.27	0.33
C5	2 days to ≤ 3 days	0.05	0.07	0.10	0.13	0.18	0.31	0.37	0.06	0.07	0.11	0.14	0.19	0.31	0.36
	3 days to ≤ 4 days	0.05	0.07	0.11	0.15	0.20	0.34	0.41	0.06	0.08	0.12	0.16	0.20	0.34	0.41
	4 days to ≤ 5 days	0.06	0.07	0.13	0.17	0.22	0.36	0.43	0.06	0.08	0.13	0.17	0.23	0.40	0.48
	5 days to ≤ 6 days	0.06	0.08	0.13	0.17	0.23	0.38	0.45	0.07	0.08	0.14	0.18	0.24	0.38	0.46
	6 days to ≤ 7 days	0.06	0.08	0.14	0.18	0.23	0.39	0.45	0.07	0.08	0.14	0.19	0.24	0.40	0.44
	7 days to ≤ 8 days	0.07	0.08	0.14	0.18	0.24	0.39	0.46	0.06	0.08	0.15	0.19	0.25	0.38	0.45
	8 days to ≤ 9 days	0.06	0.08	0.14	0.18	0.24	0.41	0.47	0.07	0.08	0.14	0.19	0.24	0.37	0.42
	9 days to ≤ 10 days	0.06	0.08	0.13	0.17	0.23	0.37	0.41	0.06	0.08	0.14	0.18	0.24	0.38	0.44
	10 days to ≤ 11 days	0.06	0.08	0.14	0.17	0.23	0.37	0.43	0.07	0.09	0.14	0.18	0.23	0.35	0.38
	11 days to ≤ 12 days	0.07	0.08	0.13	0.17	0.23	0.35	0.40	0.06	0.07	0.14	0.18	0.24	0.38	0.44
	12 days to ≤ 13 days	0.06	0.08	0.13	0.17	0.22	0.36	0.42	0.03	0.08	0.14	0.18	0.23	0.34	0.40
	13 days to ≤ 14 days	0.05	0.07	0.13	0.17	0.21	0.34	0.38	0.07	0.08	0.13	0.17	0.21	0.31	0.36
C5-DC+ C6-OH	2 days to ≤ 3 days	0.06	0.06	0.10	0.12	0.16	0.24	0.28	0.06	0.06	0.10	0.12	0.15	0.23	0.27
	3 days to ≤ 4 days	0.05	0.06	0.09	0.12	0.15	0.22	0.27	0.05	0.06	0.09	0.11	0.15	0.22	0.26
	4 days to ≤ 5 days	0.05	0.06	0.08	0.11	0.14	0.22	0.29	0.05	0.05	0.08	0.10	0.13	0.21	0.24
	5 days to ≤ 6 days	0.05	0.05	0.08	0.10	0.13	0.21	0.28	0.05	0.05	0.08	0.10	0.13	0.20	0.26

	6 days to ≤ 7 days	0.04	0.05	0.07	0.09	0.12	0.20	0.26	0.04	0.05	0.07	0.09	0.12	0.19	0.24
	7 days to ≤ 8 days	0.04	0.05	0.07	0.09	0.12	0.20	0.23	0.04	0.05	0.07	0.09	0.12	0.19	0.24
	8 days to ≤ 9 days	0.04	0.05	0.07	0.09	0.12	0.20	0.26	0.04	0.05	0.07	0.09	0.12	0.19	0.22
	9 days to ≤ 10 days	0.04	0.05	0.07	0.09	0.12	0.20	0.24	0.04	0.04	0.07	0.09	0.12	0.19	0.26
	10 days to ≤ 11 days	0.04	0.04	0.07	0.09	0.12	0.19	0.24	0.04	0.05	0.07	0.09	0.12	0.19	0.25
	11 days to ≤ 12 days	0.04	0.04	0.07	0.09	0.12	0.19	0.26	0.05	0.05	0.07	0.09	0.11	0.18	0.23
	12 days to ≤ 13 days	0.05	0.05	0.07	0.09	0.11	0.20	0.24	0.05	0.05	0.07	0.09	0.11	0.19	0.23
	13 days to ≤ 14 days	0.05	0.05	0.07	0.09	0.11	0.19	0.23	0.04	0.04	0.06	0.08	0.11	0.19	0.23
C6	2 days to ≤ 3 days	0.03	0.03	0.04	0.05	0.06	0.09	0.11	0.02	0.02	0.03	0.04	0.06	0.09	0.12
	3 days to ≤ 4 days	0.02	0.02	0.03	0.04	0.06	0.09	0.12	0.02	0.02	0.03	0.04	0.05	0.09	0.10
	4 days to ≤ 5 days	0.02	0.02	0.03	0.04	0.05	0.09	0.11	0.02	0.02	0.03	0.04	0.05	0.08	0.11
	5 days to ≤ 6 days	0.02	0.02	0.03	0.04	0.05	0.09	0.11	0.02	0.02	0.03	0.04	0.05	0.08	0.10
	6 days to ≤ 7 days	0.02	0.02	0.03	0.04	0.05	0.08	0.11	0.02	0.02	0.03	0.04	0.05	0.08	0.10
	7 days to ≤ 8 days	0.02	0.02	0.03	0.04	0.05	0.09	0.11	0.02	0.02	0.03	0.04	0.05	0.08	0.10
	8 days to ≤ 9 days	0.02	0.02	0.03	0.04	0.05	0.09	0.11	0.02	0.02	0.03	0.04	0.05	0.09	0.11
	9 days to ≤ 10 days	0.02	0.02	0.03	0.04	0.05	0.10	0.11	0.02	0.02	0.03	0.04	0.05	0.09	0.11
	10 days to ≤ 11 days	0.02	0.02	0.03	0.04	0.05	0.09	0.11	0.02	0.02	0.03	0.04	0.05	0.09	0.11
	11 days to ≤ 12 days	0.02	0.02	0.03	0.04	0.05	0.10	0.12	0.02	0.02	0.03	0.04	0.05	0.09	0.11
	12 days to ≤ 13 days	0.02	0.02	0.03	0.04	0.05	0.09	0.11	0.02	0.02	0.03	0.04	0.05	0.09	0.11
	13 days to ≤ 14 days	0.02	0.02	0.03	0.04	0.05	0.10	0.11	0.02	0.02	0.03	0.04	0.05	0.09	0.11
C6-DC	2 days to ≤ 3 days	0.04	0.04	0.07	0.10	0.13	0.21	0.26	0.04	0.04	0.07	0.10	0.14	0.22	0.28
	3 days to ≤ 4 days	0.03	0.04	0.06	0.09	0.12	0.20	0.25	0.03	0.04	0.06	0.09	0.13	0.21	0.28
	4 days to ≤ 5 days	0.03	0.03	0.06	0.08	0.12	0.20	0.26	0.03	0.03	0.06	0.08	0.12	0.21	0.30
	5 days to ≤ 6 days	0.03	0.03	0.05	0.07	0.11	0.20	0.26	0.03	0.03	0.05	0.08	0.11	0.20	0.25
	6 days to ≤ 7 days	0.03	0.03	0.05	0.07	0.11	0.21	0.25	0.03	0.03	0.05	0.08	0.11	0.21	0.27
	7 days to ≤ 8 days	0.03	0.03	0.05	0.08	0.11	0.20	0.26	0.04	0.04	0.06	0.08	0.12	0.20	0.26
	8 days to ≤ 9 days	0.04	0.04	0.06	0.08	0.11	0.20	0.25	0.03	0.03	0.06	0.08	0.12	0.21	0.28
	9 days to ≤ 10 days	0.03	0.04	0.06	0.08	0.12	0.20	0.27	0.03	0.04	0.06	0.08	0.12	0.22	0.29
	10 days to ≤ 11 days	0.03	0.03	0.06	0.08	0.11	0.20	0.27	0.03	0.04	0.06	0.08	0.12	0.22	0.30
	11 days to ≤ 12 days	0.03	0.03	0.06	0.08	0.12	0.20	0.25	0.03	0.04	0.06	0.08	0.12	0.22	0.31
	12 days to ≤ 13 days	0.04	0.04	0.06	0.08	0.11	0.21	0.27	0.03	0.04	0.06	0.08	0.11	0.23	0.30
	13 days to ≤ 14 days	0.03	0.04	0.06	0.08	0.12	0.20	0.28	0.04	0.04	0.06	0.08	0.12	0.22	0.29
C8	2 days to ≤ 3 days	0.03	0.03	0.05	0.06	0.08	0.13	0.16	0.03	0.03	0.05	0.06	0.08	0.13	0.18
	3 days to ≤ 4 days	0.02	0.03	0.04	0.06	0.08	0.14	0.18	0.02	0.02	0.04	0.06	0.08	0.13	0.17
	4 days to ≤ 5 days	0.02	0.03	0.04	0.06	0.07	0.12	0.16	0.02	0.02	0.04	0.05	0.07	0.12	0.15
	5 days to ≤ 6 days	0.02	0.02	0.04	0.05	0.07	0.12	0.15	0.02	0.02	0.04	0.05	0.07	0.12	0.16
	6 days to ≤ 7 days	0.03	0.03	0.04	0.06	0.07	0.13	0.16	0.02	0.02	0.04	0.05	0.07	0.13	0.16
	7 days to ≤ 8 days	0.02	0.02	0.04	0.06	0.07	0.14	0.17	0.02	0.02	0.04	0.05	0.07	0.14	0.16
	8 days to ≤ 9 days	0.02	0.02	0.04	0.06	0.08	0.15	0.19	0.02	0.02	0.04	0.06	0.08	0.15	0.20
	9 days to ≤ 10 days	0.02	0.02	0.04	0.06	0.08	0.15	0.21	0.02	0.02	0.04	0.06	0.08	0.15	0.21
	10 days to ≤ 11 days	0.02	0.03	0.04	0.06	0.08	0.16	0.21	0.02	0.02	0.04	0.06	0.08	0.15	0.20
	11 days to ≤ 12 days	0.02	0.02	0.04	0.06	0.08	0.16	0.21	0.03	0.03	0.04	0.06	0.07	0.15	0.20
	12 days to ≤ 13 days	0.02	0.02	0.04	0.06	0.08	0.15	0.21	0.03	0.03	0.04	0.06	0.07	0.15	0.19
	13 days to ≤ 14 days	0.02	0.03	0.04	0.06	0.08	0.15	0.20	0.02	0.02	0.04	0.05	0.07	0.14	0.18
C8:1	2 days to ≤ 3 days	0.05	0.06	0.10	0.13	0.17	0.29	0.36	0.05	0.06	0.10	0.13	0.17	0.29	0.37
	3 days to ≤ 4 days	0.05	0.06	0.10	0.13	0.18	0.31	0.39	0.05	0.06	0.10	0.13	0.18	0.31	0.39
	4 days to ≤ 5 days	0.05	0.06	0.09	0.13	0.17	0.30	0.40	0.04	0.05	0.09	0.12	0.17	0.31	0.39

	5 days to ≤ 6 days	0.05	0.05	0.09	0.12	0.16	0.30	0.38	0.05	0.05	0.09	0.12	0.16	0.28	0.39
	6 days to ≤ 7 days	0.05	0.05	0.09	0.12	0.16	0.29	0.39	0.05	0.05	0.09	0.12	0.16	0.31	0.41
	7 days to ≤ 8 days	0.04	0.05	0.08	0.12	0.16	0.31	0.46	0.05	0.05	0.09	0.12	0.17	0.30	0.41
	8 days to ≤ 9 days	0.05	0.05	0.09	0.12	0.17	0.31	0.40	0.05	0.05	0.09	0.12	0.17	0.31	0.41
	9 days to ≤ 10 days	0.05	0.05	0.09	0.13	0.17	0.32	0.45	0.05	0.05	0.09	0.12	0.17	0.31	0.41
	10 days to ≤ 11 days	0.05	0.05	0.09	0.12	0.17	0.31	0.42	0.05	0.05	0.10	0.13	0.17	0.35	0.44
	11 days to ≤ 12 days	0.05	0.06	0.10	0.13	0.17	0.31	0.39	0.05	0.06	0.10	0.13	0.18	0.32	0.41
	12 days to ≤ 13 days	0.05	0.06	0.10	0.13	0.17	0.32	0.41	0.06	0.06	0.10	0.13	0.17	0.33	0.40
	13 days to ≤ 14 days	0.04	0.05	0.09	0.13	0.18	0.33	0.45	0.05	0.06	0.09	0.13	0.17	0.30	0.39
C10	2 days to ≤ 3 days	0.03	0.03	0.05	0.07	0.10	0.20	0.27	0.03	0.03	0.05	0.07	0.10	0.20	0.28
	3 days to ≤ 4 days	0.03	0.03	0.05	0.07	0.09	0.19	0.25	0.03	0.03	0.05	0.07	0.09	0.18	0.24
	4 days to ≤ 5 days	0.03	0.03	0.05	0.06	0.08	0.13	0.16	0.02	0.03	0.04	0.06	0.08	0.14	0.19
	5 days to ≤ 6 days	0.02	0.03	0.04	0.06	0.08	0.14	0.19	0.02	0.02	0.04	0.06	0.08	0.13	0.17
	6 days to ≤ 7 days	0.02	0.03	0.04	0.06	0.08	0.13	0.18	0.03	0.03	0.04	0.06	0.07	0.13	0.17
	7 days to ≤ 8 days	0.02	0.02	0.04	0.06	0.08	0.14	0.19	0.02	0.03	0.04	0.06	0.08	0.13	0.17
	8 days to ≤ 9 days	0.02	0.02	0.04	0.06	0.08	0.14	0.18	0.02	0.02	0.04	0.06	0.08	0.15	0.19
	9 days to ≤ 10 days	0.03	0.03	0.05	0.06	0.08	0.13	0.15	0.03	0.03	0.05	0.06	0.08	0.14	0.17
	10 days to ≤ 11 days	0.03	0.03	0.05	0.06	0.08	0.14	0.17	0.03	0.03	0.05	0.06	0.08	0.14	0.16
	11 days to ≤ 12 days	0.03	0.03	0.05	0.06	0.08	0.14	0.17	0.03	0.03	0.05	0.06	0.08	0.13	0.16
	12 days to ≤ 13 days	0.03	0.03	0.05	0.06	0.08	0.14	0.18	0.03	0.03	0.05	0.06	0.08	0.14	0.16
	13 days to ≤ 14 days	0.03	0.03	0.05	0.06	0.08	0.13	0.17	0.02	0.03	0.04	0.06	0.08	0.14	0.22
C10:1	2 days to ≤ 3 days	0.02	0.03	0.05	0.07	0.10	0.16	0.20	0.03	0.03	0.05	0.07	0.09	0.15	0.19
	3 days to ≤ 4 days	0.03	0.03	0.05	0.07	0.09	0.16	0.20	0.03	0.03	0.05	0.07	0.09	0.16	0.18
	4 days to ≤ 5 days	0.02	0.02	0.04	0.06	0.08	0.14	0.17	0.02	0.02	0.04	0.06	0.08	0.14	0.17
	5 days to ≤ 6 days	0.02	0.02	0.04	0.06	0.08	0.13	0.17	0.02	0.02	0.04	0.05	0.07	0.13	0.16
	6 days to ≤ 7 days	0.02	0.02	0.04	0.05	0.07	0.13	0.16	0.02	0.02	0.04	0.05	0.07	0.13	0.17
	7 days to ≤ 8 days	0.02	0.02	0.04	0.05	0.07	0.13	0.17	0.02	0.02	0.04	0.05	0.07	0.13	0.17
	8 days to ≤ 9 days	0.03	0.03	0.04	0.06	0.07	0.13	0.15	0.02	0.02	0.04	0.05	0.07	0.13	0.16
	9 days to ≤ 10 days	0.02	0.02	0.04	0.06	0.08	0.13	0.16	0.02	0.02	0.04	0.05	0.07	0.13	0.16
	10 days to ≤ 11 days	0.02	0.02	0.04	0.05	0.07	0.13	0.16	0.02	0.02	0.04	0.06	0.08	0.13	0.17
	11 days to ≤ 12 days	0.02	0.02	0.04	0.06	0.08	0.13	0.17	0.02	0.02	0.04	0.06	0.08	0.13	0.17
	12 days to ≤ 13 days	0.02	0.02	0.04	0.06	0.08	0.13	0.17	0.02	0.02	0.04	0.06	0.08	0.14	0.18
	13 days to ≤ 14 days	0.02	0.03	0.04	0.06	0.08	0.13	0.16	0.02	0.02	0.04	0.06	0.08	0.13	0.16
C12	2 days to ≤ 3 days	0.02	0.03	0.05	0.08	0.11	0.25	0.33	0.02	0.03	0.05	0.08	0.11	0.24	0.34
	3 days to ≤ 4 days	0.03	0.03	0.05	0.07	0.09	0.19	0.23	0.03	0.03	0.05	0.07	0.09	0.19	0.23
	4 days to ≤ 5 days	0.02	0.02	0.04	0.05	0.07	0.12	0.15	0.02	0.02	0.04	0.05	0.07	0.12	0.16
	5 days to ≤ 6 days	0.01	0.02	0.03	0.05	0.07	0.11	0.16	0.02	0.02	0.03	0.05	0.06	0.10	0.15
	6 days to ≤ 7 days	0.02	0.02	0.03	0.05	0.06	0.10	0.13	0.02	0.02	0.03	0.05	0.06	0.10	0.13
	7 days to ≤ 8 days	0.02	0.02	0.03	0.05	0.06	0.11	0.14	0.02	0.02	0.03	0.05	0.06	0.10	0.14
	8 days to ≤ 9 days	0.02	0.02	0.03	0.05	0.06	0.10	0.13	0.02	0.02	0.03	0.05	0.06	0.11	0.14
	9 days to ≤ 10 days	0.01	0.02	0.03	0.05	0.06	0.10	0.13	0.02	0.02	0.03	0.05	0.06	0.10	0.14
	10 days to ≤ 11 days	0.01	0.02	0.03	0.05	0.06	0.10	0.14	0.01	0.01	0.03	0.05	0.06	0.10	0.12
	11 days to ≤ 12 days	0.02	0.02	0.03	0.05	0.06	0.11	0.15	0.01	0.01	0.03	0.05	0.06	0.10	0.12
	12 days to ≤ 13 days	0.02	0.02	0.04	0.05	0.06	0.11	0.13	0.03	0.03	0.04	0.05	0.06	0.09	0.10
	13 days to ≤ 14 days	0.03	0.03	0.04	0.05	0.07	0.09	0.10	0.02	0.02	0.04	0.05	0.06	0.10	0.12
C12:1	2 days to ≤ 3 days	0.01	0.02	0.03	0.05	0.09	0.19	0.27	0.01	0.02	0.03	0.05	0.09	0.19	0.28
	3 days to ≤ 4 days	0.01	0.01	0.03	0.04	0.07	0.16	0.21	0.01	0.01	0.03	0.04	0.07	0.16	0.21

	4 days to ≤ 5 days	0.01	0.01	0.02	0.04	0.06	0.12	0.17	0.01	0.01	0.02	0.04	0.05	0.11	0.16
	5 days to ≤ 6 days	0.01	0.01	0.02	0.03	0.05	0.10	0.14	0.01	0.01	0.02	0.03	0.05	0.11	0.16
	6 days to ≤ 7 days	0.01	0.01	0.02	0.03	0.05	0.10	0.14	0.01	0.01	0.02	0.03	0.04	0.09	0.10
	7 days to ≤ 8 days	0.01	0.01	0.02	0.03	0.04	0.09	0.12	0.01	0.01	0.02	0.03	0.04	0.09	0.12
	8 days to ≤ 9 days	0.01	0.01	0.02	0.03	0.04	0.09	0.11	0.01	0.01	0.02	0.03	0.04	0.09	0.12
	9 days to ≤ 10 days	0.01	0.01	0.02	0.03	0.04	0.09	0.12	0.01	0.01	0.02	0.03	0.04	0.09	0.12
	10 days to ≤ 11 days	0.01	0.01	0.02	0.03	0.04	0.09	0.12	0.01	0.01	0.02	0.03	0.04	0.09	0.11
	11 days to ≤ 12 days	0.01	0.01	0.02	0.03	0.04	0.09	0.12	0.01	0.01	0.02	0.03	0.04	0.09	0.12
	12 days to ≤ 13 days	0.01	0.01	0.02	0.03	0.04	0.09	0.12	0.01	0.01	0.02	0.03	0.04	0.09	0.12
	13 days to ≤ 14 days	0.01	0.01	0.02	0.03	0.04	0.08	0.10	0.01	0.01	0.02	0.03	0.04	0.09	0.12
C14	2 days to ≤ 3 days	0.08	0.09	0.16	0.20	0.26	0.40	0.47	0.07	0.09	0.14	0.18	0.23	0.37	0.44
	3 days to ≤ 4 days	0.07	0.08	0.14	0.18	0.24	0.37	0.45	0.07	0.08	0.13	0.17	0.21	0.33	0.39
	4 days to ≤ 5 days	0.06	0.07	0.12	0.16	0.20	0.32	0.40	0.06	0.07	0.11	0.14	0.18	0.27	0.33
	5 days to ≤ 6 days	0.05	0.06	0.11	0.15	0.19	0.31	0.36	0.04	0.05	0.10	0.13	0.17	0.27	0.33
	6 days to ≤ 7 days	0.05	0.06	0.10	0.14	0.18	0.29	0.36	0.05	0.05	0.09	0.12	0.16	0.25	0.31
	7 days to ≤ 8 days	0.04	0.05	0.09	0.13	0.17	0.28	0.34	0.05	0.05	0.09	0.12	0.15	0.23	0.29
	8 days to ≤ 9 days	0.05	0.05	0.09	0.12	0.17	0.27	0.33	0.04	0.05	0.08	0.11	0.15	0.23	0.29
	9 days to ≤ 10 days	0.05	0.05	0.09	0.12	0.16	0.26	0.31	0.04	0.04	0.08	0.11	0.14	0.23	0.27
	10 days to ≤ 11 days	0.04	0.05	0.08	0.11	0.15	0.25	0.29	0.04	0.04	0.08	0.10	0.13	0.23	0.27
	11 days to ≤ 12 days	0.04	0.05	0.08	0.11	0.15	0.26	0.34	0.04	0.04	0.08	0.11	0.14	0.21	0.26
	12 days to ≤ 13 days	0.04	0.05	0.08	0.11	0.14	0.25	0.30	0.03	0.04	0.07	0.10	0.13	0.21	0.25
	13 days to ≤ 14 days	0.04	0.04	0.08	0.11	0.14	0.23	0.29	0.03	0.04	0.07	0.10	0.13	0.21	0.23
C14:1	2 days to ≤ 3 days	0.03	0.04	0.07	0.09	0.12	0.23	0.29	0.03	0.04	0.06	0.09	0.12	0.23	0.29
	3 days to ≤ 4 days	0.02	0.03	0.05	0.07	0.10	0.19	0.25	0.02	0.03	0.05	0.07	0.10	0.19	0.25
	4 days to ≤ 5 days	0.02	0.03	0.04	0.06	0.08	0.14	0.17	0.02	0.03	0.04	0.06	0.07	0.12	0.15
	5 days to ≤ 6 days	0.02	0.02	0.04	0.05	0.07	0.11	0.14	0.03	0.03	0.04	0.05	0.06	0.10	0.11
	6 days to ≤ 7 days	0.03	0.03	0.04	0.05	0.06	0.09	0.10	0.03	0.03	0.04	0.05	0.06	0.10	0.11
	7 days to ≤ 8 days	0.02	0.02	0.03	0.04	0.06	0.10	0.13	0.02	0.02	0.03	0.04	0.06	0.09	0.12
	8 days to ≤ 9 days	0.02	0.02	0.03	0.04	0.06	0.09	0.13	0.02	0.02	0.03	0.04	0.06	0.10	0.15
	9 days to ≤ 10 days	0.02	0.02	0.03	0.04	0.05	0.09	0.11	0.02	0.02	0.03	0.04	0.05	0.09	0.12
	10 days to ≤ 11 days	0.02	0.02	0.03	0.04	0.05	0.09	0.10	0.02	0.02	0.03	0.04	0.05	0.09	0.10
	11 days to ≤ 12 days	0.02	0.02	0.03	0.04	0.05	0.10	0.12	0.02	0.02	0.03	0.04	0.06	0.10	0.13
	12 days to ≤ 13 days	0.02	0.02	0.03	0.04	0.05	0.09	0.10	0.02	0.02	0.03	0.04	0.05	0.09	0.10
	13 days to ≤ 14 days	0.02	0.02	0.03	0.04	0.05	0.09	0.10	0.02	0.02	0.03	0.04	0.05	0.09	0.11
C16	2 days to ≤ 3 days	0.93	1.15	2.09	2.78	3.68	5.76	6.88	0.93	1.11	1.96	2.60	3.46	5.53	6.62
	3 days to ≤ 4 days	0.80	0.99	1.76	2.41	3.29	5.53	6.69	0.78	0.96	1.66	2.26	3.06	5.10	6.20
	4 days to ≤ 5 days	0.79	0.91	1.47	1.96	2.63	4.55	5.62	0.73	0.86	1.38	1.79	2.38	4.22	5.08
	5 days to ≤ 6 days	0.72	0.84	1.33	1.73	2.29	4.07	5.37	0.67	0.78	1.26	1.66	2.22	3.80	4.93
	6 days to ≤ 7 days	0.64	0.76	1.22	1.61	2.11	3.73	4.77	0.62	0.70	1.12	1.48	1.94	3.41	4.54
	7 days to ≤ 8 days	0.58	0.68	1.11	1.47	1.96	3.33	4.53	0.55	0.63	1.03	1.35	1.80	3.15	3.97
	8 days to ≤ 9 days	0.58	0.65	1.05	1.35	1.79	3.14	4.08	0.54	0.61	0.96	1.26	1.66	2.83	3.79
	9 days to ≤ 10 days	0.51	0.59	0.96	1.23	1.62	2.95	3.90	0.48	0.55	0.87	1.13	1.48	2.61	3.28
	10 days to ≤ 11 days	0.47	0.54	0.87	1.14	1.50	2.71	3.40	0.46	0.51	0.81	1.08	1.40	2.63	3.62
	11 days to ≤ 12 days	0.46	0.53	0.85	1.09	1.45	2.73	3.74	0.42	0.49	0.78	1.03	1.40	2.36	3.19
	12 days to ≤ 13 days	0.43	0.49	0.79	1.06	1.41	2.54	3.61	0.44	0.50	0.75	0.97	1.29	2.41	3.44
	13 days to ≤ 14 days	0.42	0.49	0.75	0.99	1.30	2.37	3.02	0.40	0.43	0.69	0.90	1.19	2.47	3.31
C16:1	2 days to ≤ 3 days	0.04	0.06	0.12	0.18	0.25	0.42	0.51	0.04	0.06	0.11	0.16	0.23	0.39	0.48

	3 days to ≤ 4 days	0.04	0.05	0.10	0.14	0.21	0.39	0.49	0.03	0.04	0.09	0.13	0.19	0.35	0.44
	4 days to ≤ 5 days	0.04	0.04	0.08	0.11	0.16	0.31	0.41	0.03	0.04	0.07	0.10	0.14	0.27	0.35
	5 days to ≤ 6 days	0.03	0.04	0.07	0.09	0.13	0.27	0.36	0.03	0.03	0.06	0.08	0.12	0.25	0.35
	6 days to ≤ 7 days	0.03	0.03	0.06	0.08	0.11	0.22	0.28	0.03	0.03	0.05	0.07	0.10	0.21	0.30
	7 days to ≤ 8 days	0.03	0.03	0.05	0.07	0.10	0.21	0.29	0.03	0.03	0.05	0.07	0.09	0.17	0.20
	8 days to ≤ 9 days	0.03	0.03	0.05	0.06	0.09	0.17	0.20	0.02	0.03	0.04	0.06	0.09	0.17	0.24
	9 days to ≤ 10 days	0.02	0.03	0.04	0.06	0.09	0.18	0.24	0.02	0.02	0.04	0.06	0.08	0.16	0.22
	10 days to ≤ 11 days	0.02	0.02	0.04	0.06	0.08	0.17	0.23	0.03	0.03	0.04	0.05	0.07	0.15	0.19
	11 days to ≤ 12 days	0.02	0.02	0.04	0.06	0.08	0.17	0.24	0.02	0.02	0.04	0.05	0.07	0.14	0.17
	12 days to ≤ 13 days	0.02	0.02	0.04	0.06	0.08	0.16	0.24	0.02	0.02	0.04	0.05	0.07	0.13	0.15
	13 days to ≤ 14 days	0.02	0.02	0.04	0.05	0.07	0.13	0.17	0.02	0.02	0.04	0.05	0.07	0.15	0.24
C16:1-OH	2 days to ≤ 3 days	0.03	0.03	0.03	0.04	0.04	0.06	0.06	0.03	0.03	0.03	0.04	0.04	0.06	0.06
	3 days to ≤ 4 days	0.02	0.02	0.03	0.03	0.04	0.06	0.06	0.01	0.02	0.02	0.03	0.04	0.06	0.09
	4 days to ≤ 5 days	0.01	0.01	0.02	0.03	0.04	0.06	0.08	0.02	0.02	0.02	0.03	0.03	0.05	0.05
	5 days to ≤ 6 days	0.02	0.02	0.02	0.03	0.03	0.05	0.05	0.02	0.02	0.02	0.03	0.03	0.05	0.05
	6 days to ≤ 7 days	0.02	0.02	0.02	0.03	0.03	0.05	0.05	0.02	0.02	0.02	0.02	0.03	0.05	0.05
	7 days to ≤ 8 days	0.02	0.02	0.02	0.02	0.03	0.05	0.05	0.02	0.02	0.02	0.02	0.03	0.05	0.05
	8 days to ≤ 9 days	0.02	0.02	0.02	0.02	0.03	0.04	0.05	0.02	0.02	0.02	0.02	0.03	0.04	0.05
	9 days to ≤ 10 days	0.02	0.02	0.02	0.02	0.03	0.05	0.05	0.02	0.02	0.02	0.02	0.03	0.04	0.05
	10 days to ≤ 11 days	0.02	0.02	0.02	0.02	0.03	0.04	0.05	0.02	0.02	0.02	0.02	0.03	0.04	0.05
	11 days to ≤ 12 days	0.02	0.02	0.02	0.02	0.03	0.04	0.05	0.02	0.02	0.02	0.02	0.03	0.04	0.05
	12 days to ≤ 13 days	0.02	0.02	0.02	0.02	0.03	0.04	0.05	0.01	0.02	0.02	0.02	0.03	0.03	0.04
	13 days to ≤ 14 days	0.02	0.02	0.02	0.02	0.03	0.04	0.05	0.01	0.01	0.01	0.02	0.02	0.04	0.04
C18	2 days to ≤ 3 days	0.37	0.44	0.68	0.86	1.06	1.56	1.83	0.37	0.44	0.66	0.83	1.04	1.56	1.85
	3 days to ≤ 4 days	0.33	0.40	0.62	0.79	0.99	1.49	1.83	0.34	0.40	0.61	0.77	0.96	1.44	1.68
	4 days to ≤ 5 days	0.32	0.36	0.56	0.70	0.87	1.33	1.56	0.30	0.35	0.53	0.67	0.85	1.28	1.55
	5 days to ≤ 6 days	0.30	0.35	0.52	0.66	0.83	1.28	1.61	0.28	0.33	0.51	0.65	0.81	1.23	1.43
	6 days to ≤ 7 days	0.29	0.33	0.50	0.63	0.78	1.18	1.39	0.28	0.31	0.48	0.60	0.77	1.19	1.42
	7 days to ≤ 8 days	0.26	0.30	0.46	0.59	0.75	1.13	1.39	0.25	0.29	0.46	0.58	0.74	1.15	1.43
	8 days to ≤ 9 days	0.26	0.29	0.45	0.56	0.71	1.10	1.37	0.26	0.29	0.44	0.56	0.71	1.09	1.29
	9 days to ≤ 10 days	0.23	0.27	0.41	0.53	0.68	1.06	1.34	0.24	0.28	0.42	0.53	0.65	0.96	1.22
	10 days to ≤ 11 days	0.23	0.27	0.40	0.51	0.63	1.05	1.28	0.23	0.26	0.39	0.50	0.63	1.02	1.20
	11 days to ≤ 12 days	0.21	0.24	0.38	0.48	0.62	0.97	1.24	0.22	0.25	0.38	0.49	0.62	0.98	1.21
	12 days to ≤ 13 days	0.20	0.23	0.35	0.46	0.61	0.96	1.19	0.22	0.24	0.36	0.45	0.57	0.98	1.16
	13 days to ≤ 14 days	0.20	0.22	0.35	0.44	0.58	0.99	1.29	0.20	0.22	0.34	0.43	0.56	0.94	1.15
C18:1	2 days to ≤ 3 days	0.58	0.76	1.22	1.52	1.86	2.62	2.88	0.57	0.74	1.15	1.42	1.75	2.45	2.65
	3 days to ≤ 4 days	0.54	0.71	1.18	1.48	1.84	2.61	2.91	0.55	0.70	1.12	1.40	1.72	2.40	2.66
	4 days to ≤ 5 days	0.60	0.73	1.14	1.42	1.76	2.51	2.87	0.56	0.70	1.08	1.33	1.63	2.30	2.58
	5 days to ≤ 6 days	0.56	0.72	1.09	1.37	1.70	2.51	2.78	0.52	0.66	1.04	1.30	1.61	2.30	2.60
	6 days to ≤ 7 days	0.49	0.63	1.05	1.33	1.63	2.31	2.57	0.48	0.61	0.97	1.23	1.51	2.21	2.41
	7 days to ≤ 8 days	0.51	0.60	0.97	1.23	1.53	2.28	2.55	0.45	0.54	0.90	1.15	1.44	2.10	2.34
	8 days to ≤ 9 days	0.45	0.56	0.91	1.15	1.46	2.16	2.44	0.42	0.53	0.84	1.05	1.34	1.94	2.23
	9 days to ≤ 10 days	0.40	0.53	0.85	1.07	1.36	2.02	2.29	0.42	0.50	0.78	0.98	1.24	1.81	2.08
	10 days to ≤ 11 days	0.38	0.48	0.77	0.99	1.26	1.93	2.15	0.37	0.47	0.72	0.92	1.17	1.82	2.14
	11 days to ≤ 12 days	0.39	0.46	0.75	0.97	1.26	1.90	2.20	0.34	0.44	0.70	0.88	1.11	1.62	1.82
	12 days to ≤ 13 days	0.35	0.43	0.71	0.91	1.17	1.78	1.97	0.35	0.42	0.66	0.83	1.06	1.56	1.80
	13 days to ≤ 14 days	0.33	0.41	0.67	0.86	1.09	1.66	1.83	0.31	0.39	0.63	0.79	1.04	1.58	1.82

C18:2	2 days to ≤ 3 days	0.09	0.11	0.21	0.29	0.40	0.73	0.89	0.08	0.10	0.18	0.25	0.35	0.62	0.78
	3 days to ≤ 4 days	0.10	0.12	0.24	0.33	0.46	0.83	1.01	0.09	0.11	0.21	0.29	0.40	0.71	0.88
	4 days to ≤ 5 days	0.12	0.16	0.28	0.38	0.51	0.87	1.04	0.11	0.14	0.25	0.33	0.45	0.76	0.88
	5 days to ≤ 6 days	0.13	0.16	0.29	0.40	0.53	0.90	1.10	0.12	0.15	0.26	0.35	0.46	0.77	0.94
	6 days to ≤ 7 days	0.10	0.16	0.29	0.39	0.53	0.86	1.01	0.12	0.15	0.26	0.35	0.47	0.78	0.98
	7 days to ≤ 8 days	0.12	0.16	0.28	0.38	0.51	0.86	1.02	0.12	0.15	0.25	0.33	0.43	0.71	0.82
	8 days to ≤ 9 days	0.12	0.15	0.28	0.38	0.49	0.80	0.97	0.10	0.13	0.24	0.33	0.43	0.73	0.87
	9 days to ≤ 10 days	0.12	0.15	0.26	0.34	0.46	0.76	0.94	0.11	0.13	0.23	0.31	0.41	0.66	0.81
	10 days to ≤ 11 days	0.10	0.14	0.25	0.33	0.44	0.68	0.87	0.11	0.13	0.22	0.29	0.39	0.65	0.87
	11 days to ≤ 12 days	0.10	0.14	0.24	0.31	0.41	0.71	0.81	0.09	0.12	0.22	0.28	0.37	0.60	0.73
	12 days to ≤ 13 days	0.10	0.12	0.23	0.30	0.39	0.65	0.80	0.09	0.11	0.21	0.27	0.35	0.57	0.65
	13 days to ≤ 14 days	0.10	0.13	0.22	0.29	0.37	0.60	0.68	0.10	0.12	0.20	0.26	0.34	0.56	0.65

Abbreviation: ALA, alanine; ARG, arginine; CIT, citrulline; GLY, glycine; LEU, leucine; ILE, isoleucine; ALLO-ILE, alloisoleucine; PRO-OH, hydroxyproline; MET, methionine; ORN, ornithine; PHE, phenylalanine; PRO, proline; TYR, Tyrosine; VAL, valine; C0, free carnitine; C2, acetylcarnitine; C3, propionylcarnitine; C3-DC+C4-OH, malonylcarnitine+3-hydroxybutyrylcarnitine; C4, butyrylcarnitine+isobutyrylcarnitine; C4-DC+C5-OH, methylmalonylcarnitine+3-hydroxyisovalerylcarnitine; C5, isovalerylcarnitine+methylbutyrylcarnitine; C5-DC+C6-OH, glutarylcarnitine+3-hydroxyhexanoylcarnitine; C6, hexanoylcarnitine; C6-DC, methylglutarylcarnitine; C8, octanoylcarnitine; C8:1, octenoylcarnitine; C10, decanoylcarnitine; C10:1, decenoylcarnitine; C12, dodecanoylcarnitine; C12:1, dodecenoylcarnitine; C14, tetradecanoylcarnitine; C14:1, tetradecenoylcarnitine; C16, palmitoylcarnitine; C16:1, palmitoleylcarnitine; C16:1-OH, 3-hydroxypalmitoleylcarnitine; C18, stearoylcarnitine; C18:1, oleoylcarnitine; C18:2, linoleoylcarnitine.

Table S6. The 0.5th, 2.5th, 25th, 50th, 75th ,97.5thand 99.5th percentiles calculated by age and sex for 35 MS/MS NBS biomarkers for the preterm neonates of 2500g-4000g (μM)

		Amino acids													
		Male							Female						
**Analytes**	**Age**	**0.5th**	**2.5th**	**25th**	**50th**	**75th**	**97.5th**	**99.5th**	**0.5th**	**2.5th**	**25th**	**50th**	**75th**	**97.5th**	**99.5th**
ALA	2 days to ≤ 3 days	142.4	159.6	227.1	276.7	338.9	514.9	614.4	141.4	162.6	233.8	285.1	350.4	517.2	586.0
	3 days to ≤ 4 days	147.7	168.4	241.3	292.9	359.0	534.7	639.9	152.3	174.1	249.6	303.6	371.4	545.6	631.4
	4 days to ≤ 5 days	127.8	160.1	239.7	295.1	361.0	511.3	573.5	153.1	174.1	252.1	307.5	371.0	529.3	615.7
	5 days to ≤ 6 days	134.0	163.4	239.9	292.9	356.5	483.8	534.8	140.7	169.3	252.0	304.3	369.6	510.1	561.6
	6 days to ≤ 7 days	123.5	157.7	236.8	285.7	348.5	480.2	536.0	149.1	171.2	248.9	301.6	359.5	493.3	560.3
	7 days to ≤ 8 days	140.5	165.0	235.5	286.8	347.5	513.9	605.8	158.9	173.7	250.9	300.1	355.2	516.7	606.4
	8 days to ≤ 9 days	149.4	165.7	237.3	289.1	346.4	502.8	587.3	150.5	171.4	249.8	302.9	362.1	522.1	601.1
	9 days to ≤ 10 days	144.6	162.4	235.5	285.4	347.4	506.5	577.0	146.6	166.1	246.2	296.4	354.8	513.3	598.8
	10 days to ≤ 11 days	149.6	166.4	239.3	281.1	339.4	492.5	545.3	151.3	179.1	245.3	297.8	361.5	486.9	561.5
	11 days to ≤ 12 days	151.3	171.9	235.8	281.8	341.6	508.5	616.8	131.8	165.5	237.8	293.8	349.5	469.3	505.9
	12 days to ≤ 13 days	141.3	158.1	233.7	288.4	343.9	482.2	527.7	172.4	189.1	244.4	289.6	352.8	497.9	611.4
	13 days to ≤ 14 days	132.9	158.4	225.1	275.5	329.0	485.3	535.5	162.1	177.9	243.9	293.4	350.2	494.0	585.6
ARG	2 days to ≤ 3 days	1.4	1.8	5.4	9.6	15.7	33.3	46.9	1.3	1.7	5.0	9.0	15.0	33.2	45.9
	3 days to ≤ 4 days	1.6	2.1	6.1	10.9	17.6	37.3	51.1	1.4	1.9	5.8	10.4	17.1	36.1	50.6
	4 days to ≤ 5 days	1.6	2.2	6.9	12.7	20.5	44.7	58.2	1.4	1.8	6.2	11.5	19.0	40.9	53.7
	5 days to ≤ 6 days	1.8	2.3	7.0	12.5	20.0	43.7	61.9	1.6	2.0	6.3	11.2	18.8	40.6	54.4
	6 days to ≤ 7 days	1.6	2.0	7.0	13.0	20.6	45.3	58.1	1.6	2.1	6.9	12.3	19.8	43.5	58.9
	7 days to ≤ 8 days	1.7	2.5	7.0	12.8	20.6	45.1	56.4	1.5	2.1	6.6	12.6	19.8	41.2	56.2
	8 days to ≤ 9 days	1.9	2.5	7.7	13.6	21.3	40.9	55.1	1.6	2.3	7.5	13.4	21.8	43.4	53.8
	9 days to ≤ 10 days	2.0	2.6	8.2	14.1	21.2	45.6	56.2	1.8	2.5	7.9	13.9	21.4	38.5	48.8
	10 days to ≤ 11 days	1.7	2.2	7.8	15.4	23.5	41.4	54.2	2.0	2.9	8.1	14.4	21.6	40.2	55.1
	11 days to ≤ 12 days	2.3	3.1	9.0	14.5	23.3	46.2	60.5	1.8	2.7	8.0	14.1	21.7	43.5	53.5
	12 days to ≤ 13 days	2.1	2.8	9.2	15.7	24.1	47.1	55.8	2.4	3.1	9.2	14.8	22.2	47.7	61.1
	13 days to ≤ 14 days	2.3	2.9	8.4	15.3	23.8	45.4	60.2	1.8	2.3	8.6	15.2	23.9	43.7	60.2
CIT	2 days to ≤ 3 days	6.4	7.3	10.4	12.6	15.3	22.0	25.2	7.1	7.8	10.8	13.0	15.8	23.3	28.1
	3 days to ≤ 4 days	6.9	7.6	10.5	12.6	15.2	22.5	27.5	7.2	7.9	10.9	13.0	15.8	23.6	28.9
	4 days to ≤ 5 days	6.8	7.4	10.5	12.7	15.5	22.8	27.6	7.0	7.7	10.7	13.1	15.9	23.4	29.5
	5 days to ≤ 6 days	6.8	7.5	10.7	13.1	15.7	23.7	27.2	7.1	7.8	10.9	13.1	16.0	23.4	27.6
	6 days to ≤ 7 days	6.9	7.6	10.8	13.0	15.9	23.8	28.2	7.0	7.8	11.1	13.5	16.4	23.9	31.5
	7 days to ≤ 8 days	6.9	7.8	10.9	13.4	16.4	24.7	30.7	7.1	7.8	11.3	13.8	17.3	26.0	31.4
	8 days to ≤ 9 days	7.0	7.6	11.4	13.8	16.9	24.9	29.0	7.4	8.1	11.8	14.3	17.6	26.8	34.0
	9 days to ≤ 10 days	7.1	8.0	11.6	14.3	17.2	26.5	30.9	7.7	8.2	11.8	14.4	17.7	25.8	30.5
	10 days to ≤ 11 days	7.4	8.4	12.0	14.6	18.0	26.6	30.6	8.0	8.9	12.5	15.4	18.2	26.2	30.9
	11 days to ≤ 12 days	7.8	8.7	12.4	15.1	18.2	26.3	31.0	7.5	8.6	12.3	15.0	17.9	26.4	28.8
	12 days to ≤ 13 days	7.7	8.8	12.7	15.8	18.9	26.6	33.2	7.8	8.6	12.6	15.3	18.8	29.6	33.2
	13 days to ≤ 14 days	8.1	9.0	12.7	15.5	19.3	28.6	32.2	7.6	8.6	13.2	15.9	19.8	26.0	29.7
GLY	2 days to ≤ 3 days	262.1	296.9	412.8	496.0	602.3	892.6	1046.7	274.6	309.5	427.5	511.3	616.3	909.5	1053.4
	3 days to ≤ 4 days	252.9	282.7	394.4	475.5	579.3	865.5	1026.7	267.9	299.8	410.5	492.6	593.4	881.9	1055.0
	4 days to ≤ 5 days	224.2	251.1	347.4	420.9	519.9	809.1	962.4	226.2	260.6	363.2	436.7	537.6	814.0	952.3

	5 days to ≤ 6 days	210.2	235.9	324.7	393.2	486.9	780.3	935.6	213.2	247.8	335.3	402.9	498.4	793.3	1000.3
	6 days to ≤ 7 days	207.0	223.5	312.1	378.2	465.3	748.6	932.9	216.2	241.4	324.2	391.9	482.5	772.7	932.9
	7 days to ≤ 8 days	207.1	226.2	310.5	372.5	449.6	716.1	850.3	221.3	242.7	323.2	382.1	464.7	748.2	987.6
	8 days to ≤ 9 days	201.3	224.4	303.6	365.9	449.5	694.9	841.7	225.6	246.7	327.5	386.6	467.7	744.2	903.7
	9 days to ≤ 10 days	198.1	220.3	304.1	365.8	441.1	740.3	856.7	203.0	233.0	311.0	377.1	464.0	706.4	854.0
	10 days to ≤ 11 days	212.0	226.2	309.8	362.0	428.7	666.5	761.2	214.5	238.3	321.9	380.4	452.2	669.9	763.3
	11 days to ≤ 12 days	210.8	227.1	304.7	365.6	455.4	714.5	915.6	217.4	244.0	314.3	374.8	459.6	734.2	818.9
	12 days to ≤ 13 days	206.4	226.5	299.6	358.9	438.2	686.0	813.9	214.2	234.2	317.3	373.9	453.3	773.9	872.8
	13 days to ≤ 14 days	204.8	222.8	296.7	352.8	431.9	681.3	813.1	206.9	226.9	309.5	356.6	437.1	702.1	751.4
LEU/ILE/ALLO-ILE/PRO-OH	2 days to ≤ 3 days	77.8	86.9	120.9	143.6	170.8	238.8	273.7	80.2	89.9	124.2	147.8	175.9	244.7	280.5
	3 days to ≤ 4 days	83.9	94.1	129.2	153.1	181.9	252.8	291.2	87.2	97.1	132.7	156.8	185.6	258.3	295.7
	4 days to ≤ 5 days	84.2	93.2	133.0	159.2	189.9	268.2	310.9	84.7	94.5	134.6	160.0	191.5	269.4	311.1
	5 days to ≤ 6 days	85.5	93.6	132.8	161.1	194.0	274.0	324.5	85.7	96.9	135.8	164.5	195.0	276.2	303.8
	6 days to ≤ 7 days	85.6	94.8	134.2	161.6	195.4	281.5	329.2	88.3	98.4	137.2	164.7	195.0	283.3	322.5
	7 days to ≤ 8 days	86.4	97.8	136.4	163.4	192.7	269.4	300.3	87.6	99.0	139.5	165.5	196.7	274.2	323.3
	8 days to ≤ 9 days	86.5	94.9	136.4	162.9	192.2	263.9	300.6	86.3	94.0	139.4	167.8	201.4	282.4	330.9
	9 days to ≤ 10 days	84.0	95.0	134.1	160.8	191.9	262.9	287.2	94.8	105.1	140.5	165.7	193.1	263.5	297.3
	10 days to ≤ 11 days	92.9	100.5	136.4	161.7	190.9	256.3	300.0	93.3	103.8	138.8	164.0	193.7	270.8	303.7
	11 days to ≤ 12 days	90.4	101.5	136.1	159.0	191.2	254.7	278.0	98.5	103.5	140.9	165.2	194.4	276.4	327.5
	12 days to ≤ 13 days	85.2	96.3	133.8	161.2	191.0	266.4	300.1	92.0	99.9	137.8	162.9	191.6	262.4	299.7
	13 days to ≤ 14 days	82.5	92.5	132.5	158.5	189.3	256.2	284.9	90.6	99.8	137.6	163.5	192.8	270.0	302.8
MET	2 days to ≤ 3 days	8.3	9.6	14.7	18.6	23.3	35.2	42.4	8.6	10.0	15.4	19.4	24.2	36.4	43.8
	3 days to ≤ 4 days	8.3	9.7	14.7	18.7	23.7	36.3	44.4	8.9	10.2	15.4	19.4	24.6	37.6	44.7
	4 days to ≤ 5 days	8.4	9.7	15.1	19.3	24.5	38.4	47.4	8.8	10.2	15.3	19.6	24.7	38.1	47.1
	5 days to ≤ 6 days	8.7	9.7	15.1	19.2	24.1	37.8	47.2	8.4	9.9	15.3	19.5	24.3	38.1	46.5
	6 days to ≤ 7 days	8.8	10.0	15.1	19.2	24.1	40.1	48.0	8.4	10.0	15.6	19.6	24.3	36.6	43.3
	7 days to ≤ 8 days	8.5	10.0	15.2	19.1	24.0	38.1	45.8	8.8	10.4	15.9	19.8	24.6	35.7	41.1
	8 days to ≤ 9 days	8.8	10.2	15.8	19.6	24.6	37.1	43.6	9.2	10.8	16.3	20.7	25.3	38.9	47.6
	9 days to ≤ 10 days	8.7	10.0	15.9	19.5	24.4	36.1	41.5	8.5	10.7	16.2	20.4	25.4	35.7	40.7
	10 days to ≤ 11 days	8.8	10.1	15.8	19.7	24.8	37.6	41.0	8.0	9.9	16.6	20.6	25.4	37.1	42.2
	11 days to ≤ 12 days	8.9	10.4	15.8	19.9	24.5	35.3	42.1	9.5	11.1	16.6	20.1	25.8	37.0	46.6
	12 days to ≤ 13 days	9.6	11.2	16.3	19.9	25.1	37.2	43.6	9.4	11.4	16.6	20.5	25.4	36.9	43.3
	13 days to ≤ 14 days	8.7	9.9	15.6	19.6	24.9	36.2	45.6	10.1	11.3	17.2	20.6	25.1	37.1	39.3
ORN	2 days to ≤ 3 days	45.8	53.2	81.9	106.0	138.8	248.0	325.9	46.7	53.2	83.2	106.6	138.2	244.2	320.2
	3 days to ≤ 4 days	50.5	58.3	89.6	114.4	149.0	268.0	344.0	53.0	60.2	91.5	116.1	149.2	253.1	326.9
	4 days to ≤ 5 days	49.3	58.7	90.6	117.3	153.4	262.7	342.4	52.8	61.1	91.9	117.5	149.8	252.4	310.1
	5 days to ≤ 6 days	48.5	55.9	89.5	116.4	150.9	270.6	346.1	49.6	60.4	88.4	114.3	148.4	261.7	337.7
	6 days to ≤ 7 days	49.2	57.5	88.2	112.5	148.8	259.6	311.0	49.9	58.1	90.6	114.1	148.5	257.4	333.5
	7 days to ≤ 8 days	51.2	59.1	89.3	113.1	146.0	255.6	331.7	52.5	58.2	89.6	113.6	145.2	269.7	370.8
	8 days to ≤ 9 days	50.1	59.2	90.5	114.9	150.0	254.2	318.1	54.3	62.8	92.7	114.9	148.0	257.4	317.0
	9 days to ≤ 10 days	56.1	62.0	91.6	112.8	141.5	266.3	323.3	55.1	62.0	93.7	115.1	146.6	238.8	301.1
	10 days to ≤ 11 days	58.7	64.3	93.6	116.3	146.9	244.7	305.6	61.7	66.9	96.7	117.3	144.1	233.6	269.2
	11 days to ≤ 12 days	56.5	65.2	94.8	117.9	149.4	252.8	304.4	56.3	64.2	94.8	115.3	146.4	274.4	294.0
	12 days to ≤ 13 days	56.4	66.4	95.2	119.5	149.9	253.8	291.5	59.5	63.9	95.4	118.7	149.4	267.5	322.8

	13 days to ≤ 14 days	59.5	66.1	94.7	118.3	148.3	266.8	321.0	61.8	67.8	98.6	120.1	146.4	247.4	287.0
PHE	2 days to ≤ 3 days	34.4	37.8	48.9	56.4	65.5	88.5	101.3	34.6	38.0	49.2	56.9	65.9	89.0	101.9
	3 days to ≤ 4 days	34.7	37.7	48.7	56.2	65.5	89.7	102.8	34.8	37.8	48.8	56.4	65.5	90.1	103.7
	4 days to ≤ 5 days	32.7	35.8	46.6	54.8	65.0	93.9	112.6	32.1	35.2	46.6	54.2	64.0	89.5	104.4
	5 days to ≤ 6 days	30.7	34.4	45.9	53.8	63.8	89.5	102.0	30.8	34.2	44.8	52.5	61.9	89.4	103.0
	6 days to ≤ 7 days	29.8	33.0	43.7	51.9	61.8	89.9	104.4	30.5	32.8	44.1	51.7	61.0	88.4	99.5
	7 days to ≤ 8 days	28.6	31.9	43.4	50.9	60.2	83.3	96.2	27.5	31.1	43.1	50.6	60.5	81.1	92.4
	8 days to ≤ 9 days	25.7	29.4	41.4	49.1	58.4	79.4	89.3	28.1	31.8	42.7	50.4	60.2	83.2	94.3
	9 days to ≤ 10 days	28.3	31.0	41.1	48.5	56.2	79.4	89.4	27.7	30.4	41.6	48.7	57.6	79.0	88.7
	10 days to ≤ 11 days	27.8	30.9	40.1	46.9	55.8	77.3	88.5	28.9	31.1	40.7	47.0	55.1	78.8	89.9
	11 days to ≤ 12 days	27.4	30.5	40.4	47.1	55.7	78.7	89.9	28.7	31.2	41.1	47.3	55.3	78.0	86.8
	12 days to ≤ 13 days	26.9	29.1	38.6	45.6	54.0	78.8	91.1	29.0	32.1	40.0	47.2	55.6	75.2	89.1
	13 days to ≤ 14 days	27.6	29.9	38.9	45.2	52.8	75.6	87.2	29.3	31.5	40.4	46.1	53.5	77.9	84.7
PRO	2 days to ≤ 3 days	99.2	110.3	150.8	178.8	213.5	303.3	352.3	101.1	113.7	154.0	182.7	216.6	307.3	353.8
	3 days to ≤ 4 days	102.9	114.0	156.3	185.1	219.4	312.1	362.6	106.2	118.1	159.8	188.0	224.1	318.6	373.4
	4 days to ≤ 5 days	98.1	108.0	151.3	180.3	215.9	308.9	359.5	104.3	114.6	154.6	184.2	217.2	309.5	359.4
	5 days to ≤ 6 days	100.3	109.1	152.2	180.7	215.2	310.0	357.5	100.1	110.5	153.5	182.6	218.3	312.1	362.8
	6 days to ≤ 7 days	98.3	109.7	150.3	178.5	214.5	302.1	357.9	106.7	117.6	157.3	182.7	218.0	316.2	367.1
	7 days to ≤ 8 days	98.8	109.5	150.1	179.1	215.6	305.3	354.0	100.1	111.6	155.3	183.8	216.4	291.2	343.9
	8 days to ≤ 9 days	100.3	110.1	150.9	178.6	214.6	299.7	342.3	100.6	112.9	154.6	184.1	216.2	299.7	332.4
	9 days to ≤ 10 days	100.5	110.2	148.4	177.5	210.5	296.8	328.3	101.6	113.2	152.2	175.4	209.8	297.1	322.2
	10 days to ≤ 11 days	96.8	106.0	147.9	177.3	212.1	300.6	361.1	105.1	117.3	152.4	183.0	214.0	306.0	328.2
	11 days to ≤ 12 days	100.9	112.9	150.7	177.2	207.1	286.4	323.5	108.6	116.1	155.9	182.3	216.5	341.9	375.4
	12 days to ≤ 13 days	102.2	110.5	149.4	177.4	211.9	295.9	345.6	103.6	113.6	152.6	179.2	210.9	292.3	313.3
	13 days to ≤ 14 days	98.8	106.7	146.7	171.3	208.7	280.5	342.6	105.8	114.8	152.4	179.4	210.7	274.8	332.9
TYR	2 days to ≤ 3 days	49.2	55.8	88.1	115.1	152.6	280.7	383.6	49.0	56.6	88.9	116.5	154.4	277.0	382.0
	3 days to ≤ 4 days	52.4	60.0	95.2	124.4	164.5	300.0	403.4	53.5	61.6	97.5	127.2	167.7	295.1	393.8
	4 days to ≤ 5 days	48.7	55.6	90.3	119.5	158.0	292.7	397.2	48.5	57.0	91.4	121.7	160.6	285.5	378.3
	5 days to ≤ 6 days	46.7	53.9	86.8	114.0	149.9	284.5	391.2	47.5	55.4	90.8	118.7	156.6	277.0	341.5
	6 days to ≤ 7 days	45.8	51.3	83.5	110.6	144.8	248.9	331.5	47.0	55.3	88.6	113.7	148.9	245.6	311.9
	7 days to ≤ 8 days	44.0	51.1	81.4	106.3	138.9	235.5	324.0	45.3	52.0	83.6	107.6	142.6	248.3	317.0
	8 days to ≤ 9 days	44.2	51.4	80.2	103.5	136.4	224.1	296.1	45.3	51.3	84.4	107.9	140.9	228.2	280.2
	9 days to ≤ 10 days	41.1	49.8	75.4	97.8	128.3	205.6	242.3	42.5	49.3	79.5	103.0	130.9	198.8	257.7
	10 days to ≤ 11 days	42.1	49.2	75.5	97.2	125.5	209.2	273.0	45.1	52.2	84.1	106.2	135.7	203.7	246.8
	11 days to ≤ 12 days	41.1	46.8	75.6	97.6	121.1	189.0	249.0	46.4	52.3	76.6	97.6	124.6	211.1	266.2
	12 days to ≤ 13 days	43.7	49.1	76.0	95.9	118.5	185.7	234.2	45.0	49.6	79.4	99.0	125.9	197.3	231.1
	13 days to ≤ 14 days	39.5	44.1	71.7	91.5	115.6	179.1	229.6	41.1	47.3	74.9	96.2	126.1	196.6	232.4
VAL	2 days to ≤ 3 days	57.0	70.3	103.0	124.0	148.6	204.6	224.6	58.9	72.6	106.9	128.9	155.2	213.3	232.8
	3 days to ≤ 4 days	63.5	77.5	110.9	132.0	157.0	211.4	227.5	66.3	81.1	115.0	136.8	161.7	216.2	232.4
	4 days to ≤ 5 days	61.3	75.6	111.9	134.7	161.1	223.9	241.1	68.3	81.3	114.8	137.4	163.2	222.9	239.7
	5 days to ≤ 6 days	61.0	75.1	111.6	135.4	162.2	225.6	241.6	66.1	77.6	114.9	137.8	166.1	227.8	246.6
	6 days to ≤ 7 days	60.7	74.1	109.1	133.0	161.2	223.5	247.7	68.3	78.2	114.9	137.4	163.1	226.9	239.3
	7 days to ≤ 8 days	61.5	72.8	109.3	132.0	159.1	215.8	237.8	63.5	76.3	115.2	136.0	162.0	219.1	235.0
	8 days to ≤ 9 days	56.8	71.3	106.9	129.8	155.0	209.2	230.4	68.3	77.0	115.0	136.8	164.9	225.5	238.8
	9 days to ≤ 10 days	65.3	73.1	106.0	127.9	156.2	215.2	235.7	60.9	76.4	111.6	134.6	157.0	209.6	219.8
	10 days to ≤ 11 days	63.0	74.7	106.4	126.7	150.8	201.8	216.2	67.9	81.9	112.5	130.1	157.1	212.6	225.7
	11 days to ≤ 12 days	69.1	76.2	107.0	126.6	151.1	201.5	214.3	73.8	81.8	111.9	131.8	159.5	216.0	232.2

	12 days to ≤ 13 days	66.3	74.5	105.9	125.8	149.3	199.3	221.0	70.7	82.2	112.0	131.1	152.3	198.2	210.4
	13 days to ≤ 14 days	65.2	73.1	103.2	123.7	147.3	196.6	214.5	0.7	75.0	107.4	128.5	153.0	203.5	214.8
		Acylcarnitnes													
C0	2 days to ≤ 3 days	11.21	12.76	19.11	24.01	30.29	48.15	60.49	10.50	11.83	17.49	21.92	27.34	43.36	53.08
	3 days to ≤ 4 days	11.74	13.17	19.67	24.61	30.72	48.17	59.95	11.06	12.31	18.16	22.57	27.94	42.86	53.39
	4 days to ≤ 5 days	11.54	13.35	19.50	24.51	30.66	48.34	59.74	11.71	12.84	18.81	22.96	28.51	44.03	53.28
	5 days to ≤ 6 days	11.87	13.14	19.74	24.82	31.10	48.15	62.30	10.48	12.23	18.32	22.93	28.91	42.96	50.11
	6 days to ≤ 7 days	11.37	12.85	18.98	24.72	30.54	49.02	62.24	10.48	11.94	17.91	22.93	28.59	44.19	52.40
	7 days to ≤ 8 days	11.00	12.58	19.13	23.96	30.40	47.78	60.93	11.09	12.24	18.41	23.17	29.04	44.10	52.30
	8 days to ≤ 9 days	10.87	12.40	19.02	23.99	30.02	45.80	59.14	10.99	12.00	18.49	22.78	28.90	44.05	59.22
	9 days to ≤ 10 days	10.84	12.23	18.60	23.55	29.86	45.46	52.92	10.72	11.72	18.39	22.62	28.27	42.81	49.69
	10 days to ≤ 11 days	10.81	12.66	18.65	23.84	30.06	45.09	56.78	10.66	12.06	18.43	23.05	28.48	42.62	51.21
	11 days to ≤ 12 days	11.12	12.19	18.98	24.02	29.86	46.14	55.08	9.98	11.56	18.05	23.06	28.70	42.25	53.57
	12 days to ≤ 13 days	11.47	12.84	18.87	23.59	29.59	46.38	54.94	11.64	13.36	18.89	23.50	29.46	43.46	58.87
	13 days to ≤ 14 days	11.35	13.15	19.22	24.01	30.40	46.00	56.21	10.28	12.10	19.21	23.80	29.38	43.52	48.44
C2	2 days to ≤ 3 days	8.86	10.41	16.80	21.24	26.61	40.57	47.91	8.04	9.53	15.30	19.23	24.11	36.48	43.99
	3 days to ≤ 4 days	8.12	9.67	15.56	19.61	24.60	37.47	44.17	7.49	8.91	14.29	18.00	22.48	34.13	40.13
	4 days to ≤ 5 days	7.08	8.28	13.25	16.88	21.21	32.77	39.57	6.73	7.81	12.31	15.53	19.70	30.30	38.56
	5 days to ≤ 6 days	6.30	7.34	11.72	14.75	18.72	29.55	36.76	5.97	6.88	10.97	13.89	17.73	27.04	34.42
	6 days to ≤ 7 days	5.50	6.46	10.32	13.17	16.78	28.05	34.25	5.40	6.17	9.76	12.43	15.73	25.35	30.27
	7 days to ≤ 8 days	4.90	5.70	9.20	11.79	14.94	25.75	30.33	4.89	5.70	8.90	11.14	14.28	23.85	29.77
	8 days to ≤ 9 days	4.96	5.60	8.98	11.05	14.11	24.22	29.96	5.01	5.57	8.65	10.75	13.72	22.00	25.83
	9 days to ≤ 10 days	4.38	5.07	8.17	10.47	13.69	21.57	28.95	4.73	5.24	7.92	10.19	12.71	21.51	26.37
	10 days to ≤ 11 days	4.61	5.27	8.27	10.33	13.28	22.29	28.93	4.76	5.10	7.95	9.87	12.96	23.66	29.19
	11 days to ≤ 12 days	4.72	5.66	8.29	10.40	12.98	22.48	28.00	4.64	5.11	7.85	9.72	12.53	22.14	26.15
	12 days to ≤ 13 days	5.18	5.93	8.48	10.32	13.09	23.60	27.53	4.47	5.21	7.76	9.97	12.64	22.26	31.93
	13 days to ≤ 14 days	4.68	5.30	8.01	10.02	12.77	21.65	29.71	4.41	4.89	7.64	9.86	12.47	21.66	25.41
C3	2 days to ≤ 3 days	0.79	0.95	1.53	2.00	2.64	4.41	5.34	0.76	0.90	1.45	1.90	2.50	4.12	5.05
	3 days to ≤ 4 days	0.76	0.90	1.45	1.89	2.49	4.15	5.03	0.74	0.86	1.38	1.81	2.37	3.98	4.94
	4 days to ≤ 5 days	0.66	0.79	1.27	1.66	2.21	3.73	4.59	0.66	0.75	1.18	1.54	2.06	3.56	4.61
	5 days to ≤ 6 days	0.55	0.63	1.04	1.36	1.84	3.28	4.34	0.51	0.59	0.99	1.32	1.76	3.09	3.82
	6 days to ≤ 7 days	0.45	0.53	0.86	1.16	1.55	2.78	3.77	0.44	0.50	0.83	1.11	1.48	2.55	3.52
	7 days to ≤ 8 days	0.39	0.46	0.75	0.99	1.34	2.44	3.31	0.41	0.45	0.73	0.98	1.29	2.43	3.31
	8 days to ≤ 9 days	0.36	0.42	0.68	0.90	1.19	2.33	3.53	0.36	0.43	0.68	0.91	1.20	2.33	2.78
	9 days to ≤ 10 days	0.35	0.40	0.63	0.84	1.16	2.11	2.78	0.37	0.40	0.65	0.86	1.10	2.00	3.24
	10 days to ≤ 11 days	0.32	0.37	0.61	0.83	1.14	2.11	3.23	0.34	0.39	0.61	0.79	1.09	2.02	3.22
	11 days to ≤ 12 days	0.32	0.37	0.61	0.81	1.12	2.24	2.86	0.34	0.37	0.61	0.80	1.08	2.01	2.69
	12 days to ≤ 13 days	0.35	0.41	0.61	0.80	1.11	2.17	3.25	0.33	0.36	0.58	0.80	1.13	2.67	3.33
	13 days to ≤ 14 days	0.30	0.35	0.57	0.77	1.08	2.19	3.00	0.27	0.33	0.58	0.78	1.08	1.74	2.32
C3-DC+ C4-OH	2 days to ≤ 3 days	0.04	0.05	0.09	0.13	0.20	0.36	0.46	0.04	0.05	0.09	0.13	0.19	0.35	0.44
	3 days to ≤ 4 days	0.04	0.05	0.08	0.12	0.18	0.34	0.44	0.04	0.05	0.08	0.12	0.17	0.33	0.42
	4 days to ≤ 5 days	0.04	0.04	0.07	0.09	0.13	0.27	0.36	0.04	0.04	0.07	0.09	0.13	0.26	0.35
	5 days to ≤ 6 days	0.04	0.04	0.06	0.09	0.12	0.24	0.32	0.04	0.04	0.06	0.08	0.11	0.25	0.32
	6 days to ≤ 7 days	0.04	0.04	0.06	0.08	0.11	0.22	0.32	0.04	0.04	0.06	0.08	0.11	0.22	0.29
	7 days to ≤ 8 days	0.04	0.04	0.06	0.08	0.10	0.19	0.25	0.04	0.04	0.06	0.08	0.10	0.20	0.29
	8 days to ≤ 9 days	0.04	0.04	0.06	0.07	0.09	0.17	0.21	0.04	0.04	0.06	0.08	0.10	0.20	0.26

	9 days to ≤ 10 days	0.03	0.03	0.05	0.07	0.09	0.19	0.25	0.03	0.04	0.05	0.07	0.09	0.22	0.30
	10 days to ≤ 11 days	0.03	0.03	0.05	0.07	0.09	0.20	0.27	0.03	0.04	0.05	0.07	0.09	0.21	0.30
	11 days to ≤ 12 days	0.03	0.03	0.05	0.07	0.09	0.20	0.28	0.03	0.03	0.05	0.07	0.09	0.20	0.31
	12 days to ≤ 13 days	0.03	0.03	0.05	0.07	0.09	0.20	0.30	0.03	0.03	0.05	0.07	0.09	0.22	0.28
	13 days to ≤ 14 days	0.04	0.04	0.05	0.06	0.08	0.17	0.21	0.04	0.04	0.05	0.07	0.08	0.21	0.25
C4	2 days to ≤ 3 days	0.10	0.12	0.19	0.23	0.28	0.42	0.50	0.11	0.13	0.20	0.24	0.29	0.44	0.52
	3 days to ≤ 4 days	0.10	0.12	0.18	0.22	0.27	0.39	0.46	0.11	0.13	0.19	0.23	0.28	0.41	0.47
	4 days to ≤ 5 days	0.10	0.11	0.17	0.20	0.25	0.36	0.41	0.09	0.11	0.18	0.21	0.26	0.38	0.44
	5 days to ≤ 6 days	0.10	0.11	0.16	0.19	0.23	0.33	0.38	0.09	0.11	0.17	0.20	0.24	0.34	0.38
	6 days to ≤ 7 days	0.09	0.11	0.16	0.19	0.22	0.32	0.36	0.09	0.11	0.16	0.20	0.23	0.32	0.35
	7 days to ≤ 8 days	0.09	0.10	0.15	0.18	0.21	0.29	0.32	0.09	0.10	0.15	0.18	0.22	0.30	0.35
	8 days to ≤ 9 days	0.08	0.10	0.14	0.17	0.21	0.31	0.35	0.08	0.10	0.15	0.18	0.22	0.31	0.35
	9 days to ≤ 10 days	0.08	0.09	0.14	0.17	0.20	0.28	0.31	0.07	0.09	0.14	0.18	0.21	0.30	0.34
	10 days to ≤ 11 days	0.09	0.10	0.14	0.17	0.20	0.28	0.32	0.08	0.10	0.14	0.17	0.21	0.30	0.36
	11 days to ≤ 12 days	0.09	0.10	0.14	0.16	0.20	0.29	0.33	0.08	0.10	0.14	0.17	0.20	0.29	0.31
	12 days to ≤ 13 days	0.09	0.09	0.14	0.16	0.19	0.26	0.30	0.09	0.10	0.14	0.17	0.21	0.30	0.35
	13 days to ≤ 14 days	0.08	0.09	0.14	0.16	0.19	0.28	0.32	0.08	0.10	0.14	0.17	0.20	0.29	0.30
C4-DC+ C5-OH	2 days to ≤ 3 days	0.09	0.10	0.15	0.19	0.23	0.34	0.40	0.09	0.10	0.15	0.18	0.21	0.32	0.38
	3 days to ≤ 4 days	0.10	0.11	0.15	0.19	0.22	0.33	0.39	0.09	0.10	0.15	0.17	0.21	0.31	0.37
	4 days to ≤ 5 days	0.10	0.11	0.15	0.18	0.22	0.31	0.38	0.10	0.11	0.14	0.17	0.20	0.30	0.36
	5 days to ≤ 6 days	0.10	0.11	0.15	0.18	0.21	0.30	0.36	0.10	0.10	0.14	0.17	0.20	0.29	0.36
	6 days to ≤ 7 days	0.09	0.10	0.14	0.17	0.21	0.32	0.41	0.10	0.10	0.14	0.17	0.20	0.29	0.35
	7 days to ≤ 8 days	0.09	0.10	0.14	0.17	0.21	0.32	0.39	0.09	0.10	0.14	0.16	0.20	0.28	0.35
	8 days to ≤ 9 days	0.10	0.10	0.14	0.17	0.21	0.29	0.39	0.10	0.11	0.14	0.16	0.19	0.28	0.35
	9 days to ≤ 10 days	0.10	0.10	0.14	0.17	0.20	0.29	0.35	0.09	0.10	0.13	0.16	0.19	0.28	0.33
	10 days to ≤ 11 days	0.09	0.10	0.14	0.17	0.20	0.30	0.34	0.09	0.10	0.13	0.16	0.19	0.29	0.36
	11 days to ≤ 12 days	0.09	0.10	0.14	0.17	0.20	0.28	0.32	0.09	0.10	0.14	0.16	0.19	0.26	0.30
	12 days to ≤ 13 days	0.09	0.10	0.14	0.17	0.20	0.28	0.32	0.09	0.10	0.14	0.16	0.19	0.29	0.34
	13 days to ≤ 14 days	0.09	0.10	0.14	0.17	0.20	0.29	0.33	0.09	0.10	0.13	0.16	0.19	0.27	0.31
C5	2 days to ≤ 3 days	0.05	0.06	0.09	0.11	0.14	0.21	0.24	0.06	0.07	0.10	0.13	0.16	0.24	0.27
	3 days to ≤ 4 days	0.05	0.06	0.10	0.12	0.15	0.24	0.27	0.06	0.07	0.11	0.13	0.17	0.27	0.31
	4 days to ≤ 5 days	0.05	0.07	0.10	0.13	0.17	0.28	0.32	0.06	0.07	0.11	0.14	0.18	0.29	0.33
	5 days to ≤ 6 days	0.05	0.07	0.11	0.14	0.18	0.31	0.38	0.06	0.07	0.11	0.15	0.19	0.33	0.39
	6 days to ≤ 7 days	0.06	0.07	0.11	0.14	0.18	0.31	0.34	0.06	0.07	0.11	0.15	0.19	0.32	0.36
	7 days to ≤ 8 days	0.05	0.07	0.11	0.15	0.20	0.34	0.41	0.05	0.07	0.12	0.16	0.21	0.34	0.42
	8 days to ≤ 9 days	0.05	0.07	0.11	0.15	0.19	0.33	0.39	0.06	0.08	0.12	0.15	0.20	0.33	0.36
	9 days to ≤ 10 days	0.06	0.07	0.11	0.15	0.20	0.34	0.41	0.06	0.08	0.12	0.16	0.21	0.35	0.38
	10 days to ≤ 11 days	0.05	0.07	0.11	0.15	0.19	0.33	0.40	0.06	0.07	0.12	0.16	0.20	0.33	0.37
	11 days to ≤ 12 days	0.06	0.07	0.12	0.15	0.19	0.33	0.37	0.06	0.07	0.12	0.15	0.20	0.32	0.40
	12 days to ≤ 13 days	0.05	0.07	0.11	0.14	0.18	0.29	0.33	0.06	0.07	0.12	0.16	0.20	0.33	0.39
	13 days to ≤ 14 days	0.06	0.07	0.11	0.15	0.20	0.34	0.40	0.06	0.08	0.12	0.16	0.21	0.34	0.40
C5-DC+ C6-OH	2 days to ≤ 3 days	0.06	0.07	0.10	0.13	0.16	0.24	0.29	0.06	0.07	0.10	0.12	0.16	0.24	0.28
	3 days to ≤ 4 days	0.05	0.06	0.09	0.12	0.15	0.23	0.28	0.05	0.06	0.09	0.12	0.15	0.22	0.27
	4 days to ≤ 5 days	0.05	0.06	0.08	0.10	0.13	0.21	0.26	0.05	0.05	0.08	0.10	0.13	0.20	0.24
	5 days to ≤ 6 days	0.05	0.05	0.08	0.10	0.12	0.19	0.23	0.05	0.05	0.08	0.09	0.12	0.20	0.24

	6 days to ≤ 7 days	0.04	0.05	0.07	0.09	0.12	0.20	0.26	0.04	0.05	0.07	0.09	0.12	0.19	0.24
	7 days to ≤ 8 days	0.05	0.05	0.07	0.09	0.11	0.19	0.23	0.05	0.05	0.07	0.09	0.11	0.18	0.22
	8 days to ≤ 9 days	0.05	0.05	0.07	0.09	0.11	0.19	0.23	0.05	0.05	0.07	0.09	0.12	0.18	0.22
	9 days to ≤ 10 days	0.05	0.05	0.07	0.09	0.11	0.18	0.23	0.05	0.05	0.07	0.08	0.11	0.18	0.23
	10 days to ≤ 11 days	0.04	0.04	0.07	0.08	0.11	0.19	0.23	0.04	0.04	0.06	0.08	0.11	0.19	0.24
	11 days to ≤ 12 days	0.04	0.04	0.07	0.08	0.11	0.18	0.24	0.04	0.04	0.06	0.08	0.10	0.17	0.21
	12 days to ≤ 13 days	0.04	0.05	0.06	0.08	0.10	0.18	0.24	0.04	0.04	0.06	0.08	0.11	0.18	0.21
	13 days to ≤ 14 days	0.04	0.04	0.06	0.08	0.10	0.17	0.21	0.04	0.04	0.06	0.08	0.10	0.18	0.25
C6	2 days to ≤ 3 days	0.03	0.03	0.04	0.05	0.06	0.09	0.11	0.02	0.02	0.03	0.04	0.06	0.09	0.11
	3 days to ≤ 4 days	0.02	0.02	0.03	0.04	0.05	0.08	0.10	0.02	0.02	0.03	0.04	0.05	0.08	0.10
	4 days to ≤ 5 days	0.02	0.02	0.03	0.04	0.05	0.07	0.09	0.02	0.02	0.03	0.04	0.05	0.07	0.09
	5 days to ≤ 6 days	0.03	0.03	0.03	0.04	0.04	0.06	0.06	0.02	0.02	0.03	0.03	0.04	0.06	0.06
	6 days to ≤ 7 days	0.03	0.03	0.03	0.04	0.04	0.06	0.06	0.03	0.03	0.03	0.04	0.04	0.06	0.06
	7 days to ≤ 8 days	0.02	0.02	0.03	0.03	0.04	0.07	0.09	0.01	0.01	0.02	0.03	0.04	0.07	0.09
	8 days to ≤ 9 days	0.02	0.02	0.03	0.03	0.04	0.06	0.06	0.02	0.02	0.03	0.03	0.04	0.06	0.06
	9 days to ≤ 10 days	0.02	0.02	0.03	0.03	0.04	0.06	0.06	0.03	0.03	0.03	0.04	0.04	0.06	0.06
	10 days to ≤ 11 days	0.02	0.02	0.03	0.03	0.04	0.06	0.06	0.02	0.02	0.03	0.03	0.04	0.06	0.06
	11 days to ≤ 12 days	0.02	0.02	0.03	0.04	0.04	0.07	0.08	0.02	0.03	0.03	0.04	0.05	0.06	0.06
	12 days to ≤ 13 days	0.02	0.02	0.03	0.03	0.04	0.06	0.06	0.02	0.02	0.03	0.04	0.04	0.06	0.07
	13 days to ≤ 14 days	0.02	0.02	0.03	0.04	0.04	0.06	0.06	0.02	0.02	0.03	0.03	0.04	0.06	0.06
C6-DC	2 days to ≤ 3 days	0.04	0.04	0.07	0.10	0.14	0.23	0.29	0.03	0.04	0.07	0.10	0.14	0.24	0.31
	3 days to ≤ 4 days	0.04	0.04	0.07	0.09	0.13	0.22	0.28	0.04	0.04	0.07	0.09	0.13	0.23	0.29
	4 days to ≤ 5 days	0.04	0.04	0.06	0.08	0.11	0.20	0.26	0.03	0.04	0.06	0.08	0.11	0.20	0.29
	5 days to ≤ 6 days	0.03	0.03	0.05	0.07	0.11	0.19	0.27	0.03	0.03	0.05	0.07	0.11	0.22	0.30
	6 days to ≤ 7 days	0.03	0.03	0.05	0.07	0.10	0.19	0.26	0.03	0.03	0.05	0.07	0.11	0.20	0.27
	7 days to ≤ 8 days	0.03	0.03	0.05	0.07	0.10	0.20	0.26	0.03	0.03	0.05	0.07	0.11	0.20	0.26
	8 days to ≤ 9 days	0.03	0.03	0.05	0.07	0.11	0.20	0.27	0.04	0.04	0.06	0.08	0.11	0.20	0.28
	9 days to ≤ 10 days	0.03	0.03	0.06	0.07	0.11	0.18	0.26	0.03	0.03	0.05	0.07	0.11	0.20	0.24
	10 days to ≤ 11 days	0.04	0.04	0.06	0.08	0.11	0.21	0.29	0.03	0.04	0.06	0.08	0.11	0.22	0.27
	11 days to ≤ 12 days	0.04	0.04	0.06	0.08	0.11	0.21	0.28	0.03	0.03	0.05	0.07	0.11	0.21	0.27
	12 days to ≤ 13 days	0.04	0.04	0.06	0.08	0.11	0.20	0.23	0.04	0.04	0.06	0.08	0.11	0.22	0.26
	13 days to ≤ 14 days	0.04	0.04	0.06	0.07	0.10	0.19	0.24	0.04	0.04	0.06	0.08	0.10	0.20	0.26
C8	2 days to ≤ 3 days	0.03	0.03	0.05	0.06	0.08	0.13	0.15	0.02	0.03	0.04	0.06	0.08	0.12	0.16
	3 days to ≤ 4 days	0.02	0.03	0.04	0.06	0.07	0.12	0.16	0.03	0.03	0.04	0.05	0.07	0.12	0.16
	4 days to ≤ 5 days	0.03	0.03	0.04	0.05	0.06	0.10	0.12	0.03	0.03	0.04	0.05	0.06	0.10	0.11
	5 days to ≤ 6 days	0.03	0.03	0.04	0.05	0.06	0.10	0.12	0.02	0.02	0.03	0.04	0.06	0.10	0.14
	6 days to ≤ 7 days	0.03	0.03	0.04	0.05	0.06	0.10	0.11	0.02	0.02	0.03	0.04	0.06	0.10	0.14
	7 days to ≤ 8 days	0.02	0.02	0.04	0.05	0.06	0.10	0.12	0.02	0.02	0.03	0.04	0.06	0.10	0.14
	8 days to ≤ 9 days	0.03	0.03	0.04	0.05	0.06	0.10	0.11	0.03	0.03	0.04	0.05	0.06	0.09	0.12
	9 days to ≤ 10 days	0.02	0.02	0.03	0.05	0.06	0.10	0.13	0.02	0.02	0.03	0.04	0.06	0.09	0.13
	10 days to ≤ 11 days	0.02	0.02	0.03	0.04	0.06	0.11	0.14	0.02	0.02	0.03	0.04	0.06	0.11	0.12
	11 days to ≤ 12 days	0.03	0.03	0.04	0.05	0.06	0.10	0.11	0.02	0.02	0.03	0.04	0.06	0.09	0.12
	12 days to ≤ 13 days	0.02	0.02	0.03	0.04	0.06	0.09	0.13	0.02	0.02	0.03	0.04	0.06	0.10	0.12
	13 days to ≤ 14 days	0.02	0.02	0.04	0.04	0.06	0.10	0.11	0.02	0.02	0.03	0.04	0.05	0.09	0.11
C8:1	2 days to ≤ 3 days	0.06	0.06	0.10	0.13	0.17	0.27	0.32	0.05	0.06	0.10	0.13	0.16	0.26	0.30
	3 days to ≤ 4 days	0.05	0.06	0.10	0.13	0.17	0.28	0.33	0.06	0.06	0.10	0.13	0.17	0.28	0.36
	4 days to ≤ 5 days	0.05	0.06	0.09	0.12	0.16	0.27	0.32	0.05	0.05	0.09	0.12	0.16	0.26	0.33

	5 days to ≤ 6 days	0.05	0.05	0.09	0.12	0.15	0.26	0.31	0.04	0.05	0.08	0.11	0.15	0.26	0.32
	6 days to ≤ 7 days	0.04	0.05	0.08	0.11	0.14	0.25	0.30	0.04	0.05	0.08	0.11	0.15	0.25	0.32
	7 days to ≤ 8 days	0.04	0.05	0.08	0.11	0.15	0.26	0.34	0.04	0.05	0.08	0.11	0.15	0.24	0.33
	8 days to ≤ 9 days	0.05	0.05	0.09	0.11	0.15	0.26	0.32	0.04	0.05	0.08	0.11	0.15	0.27	0.34
	9 days to ≤ 10 days	0.05	0.05	0.09	0.11	0.16	0.26	0.35	0.05	0.05	0.09	0.12	0.15	0.26	0.35
	10 days to ≤ 11 days	0.05	0.05	0.09	0.12	0.16	0.28	0.35	0.04	0.05	0.08	0.12	0.15	0.26	0.33
	11 days to ≤ 12 days	0.05	0.05	0.09	0.12	0.16	0.26	0.36	0.05	0.05	0.09	0.12	0.16	0.26	0.33
	12 days to ≤ 13 days	0.05	0.05	0.09	0.12	0.16	0.29	0.36	0.04	0.05	0.08	0.11	0.15	0.28	0.35
	13 days to ≤ 14 days	0.05	0.05	0.09	0.12	0.16	0.28	0.36	0.05	0.06	0.09	0.12	0.16	0.27	0.32
C10	2 days to ≤ 3 days	0.03	0.03	0.06	0.08	0.11	0.21	0.27	0.03	0.03	0.06	0.08	0.11	0.20	0.27
	3 days to ≤ 4 days	0.04	0.04	0.06	0.08	0.10	0.19	0.23	0.03	0.03	0.05	0.07	0.10	0.19	0.27
	4 days to ≤ 5 days	0.03	0.03	0.05	0.06	0.08	0.14	0.18	0.02	0.03	0.04	0.06	0.08	0.14	0.22
	5 days to ≤ 6 days	0.03	0.03	0.05	0.06	0.08	0.13	0.18	0.03	0.03	0.04	0.06	0.07	0.13	0.17
	6 days to ≤ 7 days	0.02	0.03	0.04	0.06	0.07	0.12	0.16	0.03	0.03	0.04	0.06	0.07	0.12	0.16
	7 days to ≤ 8 days	0.02	0.03	0.04	0.06	0.07	0.13	0.15	0.02	0.03	0.04	0.05	0.07	0.12	0.15
	8 days to ≤ 9 days	0.02	0.03	0.04	0.06	0.07	0.12	0.15	0.02	0.03	0.04	0.06	0.07	0.13	0.17
	9 days to ≤ 10 days	0.02	0.03	0.04	0.06	0.07	0.13	0.16	0.02	0.02	0.04	0.05	0.07	0.11	0.14
	10 days to ≤ 11 days	0.03	0.03	0.04	0.06	0.07	0.13	0.15	0.03	0.03	0.04	0.05	0.07	0.13	0.17
	11 days to ≤ 12 days	0.02	0.03	0.04	0.06	0.07	0.13	0.16	0.03	0.03	0.04	0.05	0.07	0.12	0.15
	12 days to ≤ 13 days	0.03	0.03	0.04	0.05	0.07	0.13	0.16	0.03	0.03	0.04	0.05	0.07	0.13	0.19
	13 days to ≤ 14 days	0.03	0.03	0.04	0.05	0.07	0.12	0.14	0.03	0.03	0.04	0.05	0.07	0.11	0.14
C10:1	2 days to ≤ 3 days	0.03	0.03	0.06	0.08	0.10	0.16	0.19	0.04	0.04	0.06	0.07	0.09	0.15	0.16
	3 days to ≤ 4 days	0.03	0.03	0.06	0.08	0.10	0.16	0.19	0.02	0.03	0.05	0.07	0.10	0.16	0.20
	4 days to ≤ 5 days	0.03	0.03	0.05	0.07	0.09	0.15	0.19	0.03	0.03	0.05	0.06	0.08	0.13	0.16
	5 days to ≤ 6 days	0.02	0.02	0.04	0.06	0.08	0.14	0.18	0.02	0.02	0.04	0.06	0.08	0.13	0.17
	6 days to ≤ 7 days	0.02	0.02	0.04	0.05	0.07	0.12	0.15	0.02	0.02	0.04	0.06	0.08	0.13	0.16
	7 days to ≤ 8 days	0.02	0.02	0.04	0.06	0.07	0.13	0.16	0.02	0.02	0.04	0.05	0.07	0.12	0.15
	8 days to ≤ 9 days	0.02	0.03	0.04	0.06	0.07	0.12	0.15	0.02	0.02	0.04	0.06	0.07	0.13	0.15
	9 days to ≤ 10 days	0.02	0.02	0.04	0.06	0.07	0.13	0.15	0.02	0.02	0.04	0.06	0.07	0.12	0.15
	10 days to ≤ 11 days	0.02	0.02	0.04	0.06	0.08	0.14	0.18	0.02	0.03	0.04	0.05	0.07	0.12	0.14
	11 days to ≤ 12 days	0.02	0.02	0.04	0.06	0.07	0.12	0.16	0.02	0.02	0.04	0.06	0.07	0.12	0.14
	12 days to ≤ 13 days	0.02	0.03	0.04	0.06	0.07	0.13	0.16	0.02	0.03	0.04	0.06	0.08	0.12	0.16
	13 days to ≤ 14 days	0.02	0.03	0.04	0.06	0.07	0.12	0.15	0.03	0.03	0.04	0.06	0.07	0.12	0.15
C12	2 days to ≤ 3 days	0.04	0.04	0.07	0.09	0.13	0.27	0.35	0.03	0.04	0.06	0.09	0.12	0.25	0.34
	3 days to ≤ 4 days	0.03	0.03	0.06	0.08	0.11	0.22	0.28	0.04	0.04	0.06	0.08	0.10	0.20	0.24
	4 days to ≤ 5 days	0.03	0.03	0.05	0.06	0.08	0.13	0.16	0.03	0.03	0.05	0.06	0.08	0.13	0.16
	5 days to ≤ 6 days	0.02	0.02	0.04	0.06	0.07	0.12	0.14	0.02	0.02	0.04	0.05	0.07	0.12	0.15
	6 days to ≤ 7 days	0.02	0.02	0.04	0.05	0.07	0.11	0.14	0.02	0.02	0.04	0.05	0.07	0.11	0.14
	7 days to ≤ 8 days	0.02	0.02	0.04	0.05	0.07	0.11	0.13	0.03	0.03	0.04	0.05	0.07	0.10	0.11
	8 days to ≤ 9 days	0.02	0.02	0.04	0.05	0.07	0.10	0.13	0.02	0.02	0.04	0.05	0.07	0.11	0.13
	9 days to ≤ 10 days	0.02	0.02	0.04	0.05	0.07	0.11	0.15	0.03	0.03	0.04	0.05	0.06	0.10	0.11
	10 days to ≤ 11 days	0.02	0.02	0.04	0.05	0.07	0.11	0.14	0.02	0.02	0.04	0.05	0.07	0.12	0.16
	11 days to ≤ 12 days	0.02	0.02	0.04	0.05	0.07	0.11	0.12	0.03	0.03	0.04	0.05	0.06	0.10	0.11
	12 days to ≤ 13 days	0.03	0.03	0.04	0.05	0.06	0.10	0.11	0.03	0.03	0.04	0.05	0.07	0.11	0.12
	13 days to ≤ 14 days	0.03	0.03	0.04	0.05	0.06	0.09	0.10	0.03	0.03	0.04	0.05	0.06	0.09	0.09
C12:1	2 days to ≤ 3 days	0.02	0.02	0.04	0.07	0.10	0.21	0.29	0.02	0.02	0.04	0.06	0.10	0.21	0.28
	3 days to ≤ 4 days	0.02	0.02	0.04	0.06	0.08	0.17	0.21	0.02	0.02	0.04	0.05	0.08	0.17	0.20

	4 days to ≤ 5 days	0.02	0.02	0.03	0.04	0.06	0.13	0.19	0.02	0.02	0.03	0.04	0.06	0.13	0.17
	5 days to ≤ 6 days	0.01	0.01	0.02	0.03	0.05	0.12	0.16	0.01	0.01	0.02	0.03	0.05	0.11	0.16
	6 days to ≤ 7 days	0.01	0.01	0.02	0.03	0.05	0.10	0.13	0.01	0.01	0.02	0.03	0.05	0.09	0.12
	7 days to ≤ 8 days	0.01	0.01	0.02	0.03	0.05	0.10	0.14	0.01	0.01	0.02	0.03	0.05	0.09	0.12
	8 days to ≤ 9 days	0.01	0.01	0.02	0.03	0.04	0.09	0.11	0.01	0.01	0.02	0.03	0.05	0.10	0.16
	9 days to ≤ 10 days	0.01	0.01	0.02	0.03	0.04	0.09	0.11	0.01	0.01	0.02	0.03	0.04	0.09	0.10
	10 days to ≤ 11 days	0.01	0.01	0.02	0.03	0.04	0.09	0.11	0.01	0.01	0.02	0.03	0.04	0.10	0.15
	11 days to ≤ 12 days	0.01	0.01	0.02	0.03	0.05	0.09	0.11	0.01	0.01	0.02	0.03	0.05	0.09	0.11
	12 days to ≤ 13 days	0.01	0.01	0.02	0.03	0.04	0.09	0.12	0.01	0.01	0.02	0.03	0.05	0.10	0.14
	13 days to ≤ 14 days	0.01	0.01	0.02	0.03	0.04	0.08	0.10	0.01	0.01	0.02	0.03	0.04	0.08	0.09
C14	2 days to ≤ 3 days	0.09	0.11	0.17	0.21	0.27	0.41	0.49	0.09	0.10	0.15	0.19	0.24	0.38	0.45
	3 days to ≤ 4 days	0.09	0.10	0.16	0.19	0.24	0.36	0.42	0.08	0.09	0.14	0.17	0.22	0.33	0.40
	4 days to ≤ 5 days	0.07	0.08	0.13	0.17	0.21	0.32	0.38	0.07	0.08	0.12	0.15	0.19	0.29	0.34
	5 days to ≤ 6 days	0.06	0.07	0.12	0.16	0.20	0.30	0.37	0.06	0.07	0.11	0.14	0.18	0.27	0.33
	6 days to ≤ 7 days	0.06	0.07	0.11	0.15	0.19	0.30	0.35	0.06	0.07	0.10	0.13	0.16	0.25	0.30
	7 days to ≤ 8 days	0.05	0.06	0.10	0.14	0.18	0.28	0.36	0.05	0.06	0.09	0.12	0.15	0.24	0.31
	8 days to ≤ 9 days	0.05	0.05	0.10	0.13	0.17	0.27	0.32	0.05	0.05	0.09	0.12	0.15	0.24	0.29
	9 days to ≤ 10 days	0.05	0.05	0.09	0.12	0.16	0.26	0.34	0.04	0.05	0.08	0.11	0.14	0.23	0.27
	10 days to ≤ 11 days	0.05	0.05	0.09	0.12	0.15	0.25	0.30	0.05	0.06	0.09	0.11	0.14	0.23	0.30
	11 days to ≤ 12 days	0.05	0.05	0.09	0.11	0.14	0.23	0.29	0.05	0.05	0.08	0.10	0.13	0.22	0.28
	12 days to ≤ 13 days	0.05	0.05	0.08	0.11	0.14	0.23	0.30	0.05	0.05	0.08	0.10	0.13	0.22	0.31
	13 days to ≤ 14 days	0.04	0.05	0.08	0.10	0.13	0.21	0.25	0.04	0.05	0.07	0.09	0.12	0.20	0.25
C14:1	2 days to ≤ 3 days	0.03	0.04	0.07	0.10	0.14	0.26	0.33	0.03	0.04	0.07	0.09	0.13	0.24	0.29
	3 days to ≤ 4 days	0.03	0.04	0.06	0.08	0.11	0.20	0.25	0.03	0.04	0.06	0.08	0.11	0.20	0.25
	4 days to ≤ 5 days	0.03	0.03	0.05	0.06	0.08	0.13	0.15	0.03	0.03	0.05	0.06	0.08	0.13	0.15
	5 days to ≤ 6 days	0.02	0.03	0.04	0.05	0.07	0.12	0.14	0.02	0.03	0.04	0.05	0.07	0.11	0.14
	6 days to ≤ 7 days	0.03	0.03	0.04	0.05	0.06	0.09	0.10	0.03	0.03	0.04	0.05	0.06	0.09	0.10
	7 days to ≤ 8 days	0.03	0.03	0.04	0.05	0.06	0.09	0.10	0.02	0.02	0.04	0.05	0.06	0.09	0.10
	8 days to ≤ 9 days	0.03	0.03	0.04	0.05	0.06	0.09	0.10	0.03	0.03	0.04	0.05	0.06	0.09	0.10
	9 days to ≤ 10 days	0.03	0.03	0.04	0.05	0.06	0.09	0.11	0.02	0.02	0.04	0.05	0.06	0.09	0.11
	10 days to ≤ 11 days	0.03	0.03	0.04	0.05	0.06	0.09	0.10	0.02	0.02	0.03	0.05	0.06	0.11	0.14
	11 days to ≤ 12 days	0.03	0.03	0.04	0.05	0.06	0.09	0.10	0.03	0.03	0.04	0.05	0.06	0.09	0.10
	12 days to ≤ 13 days	0.03	0.03	0.04	0.05	0.06	0.10	0.12	0.03	0.03	0.04	0.05	0.06	0.10	0.11
	13 days to ≤ 14 days	0.01	0.02	0.03	0.04	0.06	0.09	0.11	0.01	0.02	0.03	0.04	0.06	0.09	0.10
C16	2 days to ≤ 3 days	1.22	1.48	2.52	3.27	4.16	6.26	7.33	1.14	1.37	2.34	3.03	3.85	5.86	6.91
	3 days to ≤ 4 days	1.05	1.28	2.26	3.01	3.88	5.98	7.15	1.00	1.21	2.09	2.78	3.58	5.50	6.81
	4 days to ≤ 5 days	0.91	1.07	1.76	2.35	3.16	5.27	6.51	0.83	0.97	1.63	2.19	2.91	4.83	6.18
	5 days to ≤ 6 days	0.82	0.94	1.55	2.04	2.68	4.63	5.67	0.78	0.90	1.48	1.91	2.56	4.28	5.07
	6 days to ≤ 7 days	0.73	0.87	1.38	1.86	2.45	4.17	5.48	0.69	0.78	1.28	1.70	2.28	3.82	4.98
	7 days to ≤ 8 days	0.67	0.76	1.25	1.68	2.23	3.89	4.75	0.61	0.71	1.16	1.56	2.05	3.75	4.65
	8 days to ≤ 9 days	0.64	0.73	1.16	1.53	2.02	3.80	4.54	0.63	0.71	1.10	1.47	1.94	3.42	4.62
	9 days to ≤ 10 days	0.59	0.65	1.05	1.38	1.85	3.26	4.45	0.54	0.60	0.97	1.27	1.72	3.40	4.30
	10 days to ≤ 11 days	0.54	0.60	1.00	1.31	1.74	3.20	4.12	0.53	0.60	0.90	1.22	1.67	3.36	4.65
	11 days to ≤ 12 days	0.51	0.59	0.94	1.22	1.61	3.22	4.24	0.49	0.55	0.87	1.11	1.51	3.15	3.97
	12 days to ≤ 13 days	0.49	0.56	0.88	1.15	1.56	3.16	4.62	0.49	0.57	0.82	1.04	1.45	3.86	4.61
	13 days to ≤ 14 days	0.47	0.52	0.82	1.08	1.42	2.75	3.93	0.44	0.48	0.75	0.99	1.29	2.70	3.38
C16:1	2 days to ≤ 3 days	0.05	0.07	0.14	0.20	0.27	0.43	0.51	0.05	0.07	0.13	0.18	0.25	0.40	0.47

	3 days to ≤ 4 days	0.04	0.06	0.12	0.17	0.24	0.40	0.48	0.04	0.06	0.11	0.16	0.22	0.37	0.45
	4 days to ≤ 5 days	0.03	0.05	0.08	0.12	0.17	0.32	0.42	0.03	0.04	0.08	0.11	0.16	0.30	0.38
	5 days to ≤ 6 days	0.03	0.04	0.07	0.10	0.14	0.27	0.33	0.03	0.04	0.07	0.09	0.13	0.25	0.30
	6 days to ≤ 7 days	0.03	0.03	0.06	0.09	0.12	0.24	0.30	0.03	0.03	0.06	0.08	0.12	0.22	0.28
	7 days to ≤ 8 days	0.02	0.03	0.05	0.08	0.11	0.22	0.31	0.02	0.03	0.05	0.07	0.10	0.21	0.27
	8 days to ≤ 9 days	0.03	0.03	0.05	0.07	0.10	0.22	0.32	0.03	0.03	0.05	0.07	0.09	0.19	0.22
	9 days to ≤ 10 days	0.03	0.03	0.05	0.06	0.09	0.19	0.24	0.02	0.02	0.04	0.06	0.08	0.19	0.22
	10 days to ≤ 11 days	0.02	0.03	0.04	0.06	0.09	0.18	0.29	0.02	0.03	0.04	0.06	0.08	0.17	0.20
	11 days to ≤ 12 days	0.02	0.03	0.04	0.06	0.08	0.18	0.25	0.02	0.02	0.04	0.05	0.07	0.14	0.15
	12 days to ≤ 13 days	0.02	0.02	0.04	0.05	0.08	0.17	0.27	0.02	0.02	0.04	0.05	0.08	0.20	0.29
	13 days to ≤ 14 days	0.02	0.02	0.04	0.05	0.07	0.14	0.17	0.03	0.03	0.04	0.05	0.06	0.10	0.12
C16:1-OH	2 days to ≤ 3 days	0.03	0.03	0.03	0.04	0.04	0.06	0.06	0.03	0.03	0.03	0.03	0.04	0.06	0.06
	3 days to ≤ 4 days	0.02	0.02	0.03	0.03	0.04	0.06	0.06	0.01	0.02	0.02	0.03	0.04	0.06	0.10
	4 days to ≤ 5 days	0.01	0.01	0.02	0.03	0.04	0.06	0.11	0.02	0.02	0.02	0.03	0.03	0.05	0.06
	5 days to ≤ 6 days	0.01	0.01	0.02	0.03	0.03	0.05	0.07	0.02	0.02	0.02	0.03	0.03	0.05	0.06
	6 days to ≤ 7 days	0.02	0.02	0.02	0.03	0.03	0.05	0.06	0.02	0.02	0.02	0.03	0.03	0.05	0.06
	7 days to ≤ 8 days	0.02	0.02	0.02	0.03	0.03	0.05	0.05	0.02	0.02	0.02	0.02	0.03	0.05	0.06
	8 days to ≤ 9 days	0.02	0.02	0.02	0.02	0.03	0.05	0.05	0.02	0.02	0.02	0.02	0.03	0.05	0.06
	9 days to ≤ 10 days	0.02	0.02	0.02	0.02	0.03	0.05	0.06	0.02	0.02	0.02	0.02	0.03	0.04	0.05
	10 days to ≤ 11 days	0.02	0.02	0.02	0.02	0.03	0.04	0.05	0.02	0.02	0.02	0.02	0.03	0.04	0.05
	11 days to ≤ 12 days	0.01	0.02	0.02	0.02	0.03	0.04	0.05	0.02	0.02	0.02	0.02	0.03	0.05	0.05
	12 days to ≤ 13 days	0.01	0.02	0.02	0.02	0.03	0.05	0.05	0.01	0.02	0.02	0.02	0.03	0.04	0.05
	13 days to ≤ 14 days	0.01	0.02	0.02	0.02	0.03	0.04	0.05	0.01	0.02	0.02	0.02	0.03	0.04	0.05
C18	2 days to ≤ 3 days	0.43	0.49	0.73	0.91	1.12	1.65	1.95	0.40	0.46	0.71	0.88	1.09	1.63	1.91
	3 days to ≤ 4 days	0.38	0.45	0.68	0.85	1.06	1.59	1.90	0.38	0.44	0.66	0.83	1.03	1.55	1.83
	4 days to ≤ 5 days	0.32	0.38	0.58	0.73	0.92	1.42	1.68	0.32	0.37	0.57	0.72	0.91	1.38	1.73
	5 days to ≤ 6 days	0.31	0.36	0.54	0.68	0.84	1.33	1.62	0.31	0.35	0.53	0.67	0.84	1.27	1.56
	6 days to ≤ 7 days	0.29	0.33	0.49	0.64	0.80	1.26	1.53	0.28	0.33	0.49	0.63	0.80	1.27	1.51
	7 days to ≤ 8 days	0.27	0.31	0.47	0.59	0.75	1.18	1.41	0.27	0.31	0.46	0.57	0.73	1.21	1.50
	8 days to ≤ 9 days	0.26	0.29	0.44	0.56	0.71	1.16	1.51	0.27	0.31	0.46	0.58	0.72	1.11	1.34
	9 days to ≤ 10 days	0.24	0.27	0.41	0.53	0.68	1.10	1.29	0.22	0.27	0.40	0.52	0.68	1.13	1.44
	10 days to ≤ 11 days	0.25	0.26	0.41	0.52	0.67	1.04	1.24	0.24	0.28	0.40	0.51	0.67	1.10	1.45
	11 days to ≤ 12 days	0.22	0.24	0.39	0.48	0.63	1.03	1.33	0.23	0.25	0.39	0.51	0.64	1.07	1.37
	12 days to ≤ 13 days	0.22	0.25	0.38	0.48	0.61	1.09	1.42	0.24	0.27	0.39	0.48	0.61	1.12	1.36
	13 days to ≤ 14 days	0.21	0.23	0.35	0.46	0.59	1.05	1.28	0.21	0.23	0.36	0.45	0.58	1.05	1.39
C18:1	2 days to ≤ 3 days	0.69	0.86	1.28	1.55	1.87	2.55	2.76	0.64	0.80	1.19	1.45	1.75	2.38	2.59
	3 days to ≤ 4 days	0.66	0.85	1.28	1.55	1.87	2.58	2.79	0.65	0.80	1.20	1.44	1.73	2.37	2.56
	4 days to ≤ 5 days	0.62	0.79	1.21	1.48	1.79	2.52	2.73	0.61	0.75	1.13	1.39	1.70	2.38	2.59
	5 days to ≤ 6 days	0.55	0.72	1.16	1.43	1.74	2.43	2.66	0.55	0.71	1.08	1.33	1.61	2.20	2.38
	6 days to ≤ 7 days	0.58	0.71	1.08	1.33	1.62	2.32	2.53	0.50	0.64	1.01	1.24	1.51	2.10	2.33
	7 days to ≤ 8 days	0.55	0.66	0.99	1.25	1.55	2.23	2.51	0.43	0.59	0.92	1.14	1.43	2.04	2.23
	8 days to ≤ 9 days	0.43	0.58	0.92	1.17	1.46	2.15	2.51	0.45	0.57	0.87	1.09	1.38	1.97	2.20
	9 days to ≤ 10 days	0.43	0.53	0.84	1.07	1.35	1.98	2.18	0.42	0.50	0.75	0.97	1.23	1.84	2.04
	10 days to ≤ 11 days	0.41	0.51	0.79	1.00	1.26	1.91	2.11	0.35	0.48	0.72	0.91	1.20	1.82	2.21
	11 days to ≤ 12 days	0.39	0.48	0.72	0.93	1.20	1.75	2.05	0.42	0.47	0.66	0.87	1.10	1.69	1.85
	12 days to ≤ 13 days	0.37	0.47	0.71	0.89	1.13	1.76	1.89	0.37	0.45	0.66	0.84	1.06	1.58	1.78
	13 days to ≤ 14 days	0.36	0.43	0.66	0.81	1.03	1.60	1.82	0.29	0.39	0.62	0.78	0.99	1.49	1.71

C18:2	2 days to ≤ 3 days	0.07	0.09	0.16	0.22	0.31	0.54	0.69	0.06	0.08	0.14	0.20	0.28	0.48	0.61
	3 days to ≤ 4 days	0.08	0.10	0.19	0.26	0.36	0.59	0.72	0.07	0.09	0.17	0.23	0.32	0.53	0.66
	4 days to ≤ 5 days	0.10	0.12	0.24	0.33	0.42	0.67	0.77	0.09	0.11	0.21	0.29	0.38	0.61	0.73
	5 days to ≤ 6 days	0.10	0.13	0.26	0.35	0.45	0.69	0.80	0.09	0.12	0.23	0.31	0.39	0.61	0.71
	6 days to ≤ 7 days	0.10	0.13	0.25	0.33	0.43	0.67	0.77	0.09	0.12	0.23	0.30	0.38	0.58	0.65
	7 days to ≤ 8 days	0.10	0.14	0.24	0.32	0.42	0.64	0.75	0.09	0.12	0.23	0.29	0.37	0.56	0.61
	8 days to ≤ 9 days	0.10	0.14	0.24	0.31	0.39	0.60	0.71	0.07	0.10	0.21	0.28	0.35	0.53	0.63
	9 days to ≤ 10 days	0.10	0.12	0.21	0.28	0.36	0.60	0.68	0.09	0.12	0.19	0.26	0.33	0.49	0.57
	10 days to ≤ 11 days	0.10	0.12	0.21	0.27	0.36	0.56	0.66	0.08	0.10	0.19	0.24	0.31	0.48	0.53
	11 days to ≤ 12 days	0.09	0.12	0.19	0.25	0.34	0.55	0.66	0.10	0.11	0.18	0.22	0.30	0.46	0.56
	12 days to ≤ 13 days	0.10	0.12	0.19	0.25	0.33	0.50	0.59	0.08	0.10	0.18	0.23	0.30	0.46	0.52
	13 days to ≤ 14 days	0.09	0.11	0.19	0.24	0.32	0.49	0.60	0.09	0.10	0.17	0.22	0.28	0.42	0.48

Abbreviation: ALA, alanine; ARG, arginine; CIT, citrulline; GLY, glycine; LEU, leucine; ILE, isoleucine; ALLO-ILE, alloisoleucine; PRO-OH, hydroxyproline; MET, methionine; ORN, ornithine; PHE, phenylalanine; PRO, proline; TYR, Tyrosine; VAL, valine; C0, free carnitine; C2, acetylcarnitine; C3, propionylcarnitine; C3-DC+C4-OH, malonylcarnitine+3-hydroxybutyrylcarnitine; C4, butyrylcarnitine+isobutyrylcarnitine; C4-DC+C5-OH, methylmalonylcarnitine+3-hydroxyisovalerylcarnitine; C5, isovalerylcarnitine+methylbutyrylcarnitine; C5-DC+C6-OH, glutarylcarnitine+3-hydroxyhexanoylcarnitine; C6, hexanoylcarnitine; C6-DC, methylglutarylcarnitine; C8, octanoylcarnitine; C8:1, octenoylcarnitine; C10, decanoylcarnitine; C10:1, decenoylcarnitine; C12, dodecanoylcarnitine; C12:1, dodecenoylcarnitine; C14, tetradecanoylcarnitine; C14:1, tetradecenoylcarnitine; C16, palmitoylcarnitine; C16:1, palmitoleylcarnitine; C16:1-OH, 3-hydroxypalmitoleylcarnitine; C18, stearoylcarnitine; C18:1, oleoylcarnitine; C18:2, linoleoylcarnitine.

Figure S1. Dynamic change of acylcarnitines whose levels were irrelevant to birth weight. Data of VLBW, LBW and NBW are shown in red, green and blue boxes with whiskers, respectively. The boxes extend from the 25th to the 75th percentile, with whiskers extending to the 2.5th or 97.5th percentile. Medians are shown as white circles in the body of boxes, and are linked with red (VLBW), green (LBW) or blue (NBW) line to shown the dynamics. Abbreviations are listed in the legend of Table 1.

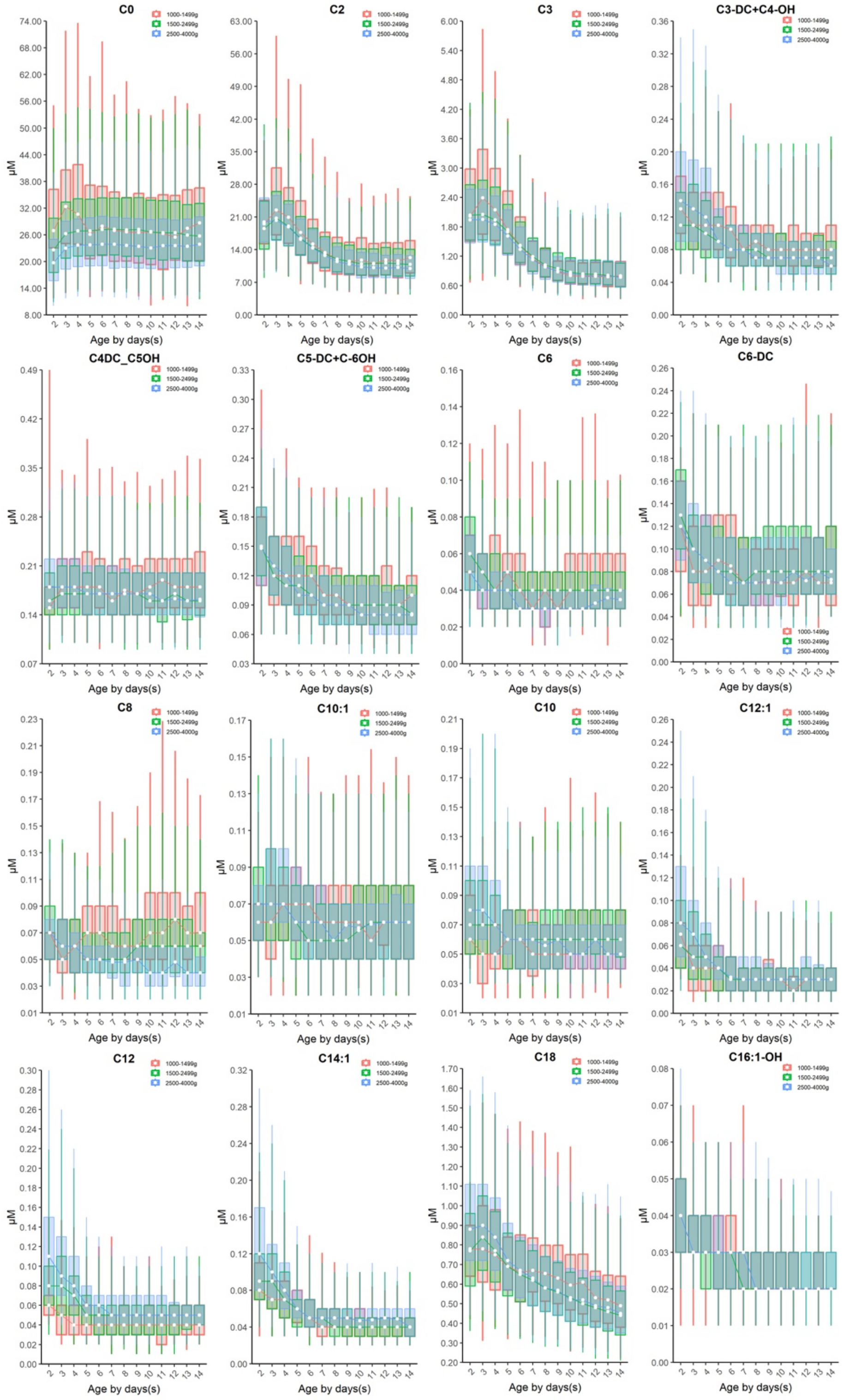

Supplement: Supplementary file 1 — Supplementary Material 1 [file 12887_2024_4865_MOESM1_ESM.pdf]
